# BRAF^Δβ3-αC^ in-frame deletion mutants differ in their dimerization propensity, HSP90 dependence, and druggability

**DOI:** 10.1126/sciadv.ade7486

**Published:** 2023-09-01

**Authors:** Manuel Lauinger, Daniel Christen, Rhena F. U. Klar, Carole Roubaty, Christoph E. Heilig, Michael Stumpe, Jennifer J. Knox, Nikolina Radulovich, Laura Tamblyn, Irene Y. Xie, Peter Horak, Andrea Forschner, Michael Bitzer, Uwe A. Wittel, Melanie Boerries, Claudia R. Ball, Christoph Heining, Hanno Glimm, Martina Fröhlich, Daniel Hübschmann, Steven Gallinger, Ralph Fritsch, Stefan Fröhling, Grainne M. O’Kane, Jörn Dengjel, Tilman Brummer

**Affiliations:** ^1^ Institute of Molecular Medicine, ZBMZ, Faculty of Medicine, University of Freiburg, 79104 Freiburg, Germany.; ^2^ Faculty of Biology, University of Freiburg, Freiburg, Germany.; ^3^ German Cancer Consortium (DKTK), partner site Freiburg and German Cancer Research Center (DKFZ), 69120 Heidelberg, Germany.; ^4^ Freeze-O Organoid Bank, University Medical Center, Faculty of Medicine, University of Freiburg, Freiburg, Germany.; ^5^ Department of Internal Medicine I (Hematology, Oncology, and Stem Cell Transplantation), University Hospital of Freiburg, Freiburg, Germany.; ^6^ Institute of Medical Bioinformatics and Systems Medicine (IBSM), Freiburg University Medical Center, Faculty of Medicine, University of Freiburg, Freiburg, Germany.; ^7^ Department of Biology, University of Fribourg, 1700 Fribourg, Switzerland.; ^8^ Division of Translational Medical Oncology, National Center for Tumor Diseases (NCT) Heidelberg and German Cancer Research Center (DKFZ), 69120 Heidelberg, Germany.; ^9^ German Cancer Consortium (DKTK), Heidelberg, Germany.; ^10^ PanCuRx Translational Research Initiative, Ontario Institute for Cancer Research, Toronto, Ontario, Canada.; ^11^ Princess Margaret Cancer Centre, University Health Network, Toronto, Ontario, Canada.; ^12^ Department of Dermatology, University Hospital of Tübingen, Tübingen, Germany.; ^13^ German Cancer Consortium (DKTK), DKFZ partner site Tübingen, Eberhard Karls University, Tübingen, Germany.; ^14^ Center for Personalized Medicine Tübingen, Eberhard Karls University, Tübingen, Germany.; ^15^ Department of Internal Medicine I, Eberhard-Karls University, Tübingen, Germany.; ^16^ Department of General and Visceral Surgery, University of Freiburg Medical Center, Faculty of Medicine, 79106 Freiburg, Germany.; ^17^ Comprehensive Cancer Center Freiburg (CCCF), Medical Center, Faculty of Medicine, University of Freiburg, 79106 Freiburg, Germany.; ^18^ Department for Translational Medical Oncology, National Center for Tumor Diseases (NCT/UCC), Dresden, Germany.; ^19^ German Cancer Research Center (DKFZ), Heidelberg, Germany.; ^20^ Faculty of Medicine and University Hospital Carl Gustav Carus, Technische Universität Dresden, Dresden, Germany.; ^21^ Helmholtz-Zentrum Dresden–Rossendorf (HZDR), Dresden, Germany.; ^22^ Translational Medical Oncology, Faculty of Medicine and University Hospital Carl Gustav Carus, Technische Universität Dresden, Dresden, Germany.; ^23^ German Cancer Consortium (DKTK), Dresden, Germany.; ^24^ Technische Universität Dresden, Faculty of Biology, Technische Universität Dresden, Dresden, Germany.; ^25^ Translational Functional Cancer Genomics, National Center for Tumor Diseases (NCT) and German Cancer Research Center (DKFZ), Heidelberg, Germany.; ^26^ Computational Oncology Group, Molecular Precision Oncology Program, National Center for Tumor Diseases (NCT) Heidelberg and German Cancer Research Center (DKFZ), Heidelberg, Germany.; ^27^ Pattern Recognition and Digital Medicine Group, Heidelberg Institute for Stem Cell Technology and Experimental Medicine (HI-STEM), Heidelberg, Germany.; ^28^ Department of Medical Oncology and Haematology, University Hospital of Zurich, Zurich, Switzerland.; ^29^ Center for Biological Signalling Studies BIOSS, University of Freiburg, 79104 Freiburg, Germany.

## Abstract

In-frame *BRAF* exon 12 deletions are increasingly identified in various tumor types. The resultant BRAF^Δβ3-αC^ oncoproteins usually lack five amino acids in the β3-αC helix linker and sometimes contain de novo insertions. The dimerization status of BRAF^Δβ3-αC^ oncoproteins, their precise pathomechanism, and their direct druggability by RAF inhibitors (RAFi) has been under debate. Here, we functionally characterize BRAF^ΔLNVTAP>F^ and two novel mutants, BRAF^delinsFS^ and BRAF^ΔLNVT>F^, and compare them with other BRAF^Δβ3-αC^ oncoproteins. We show that BRAF^Δβ3-αC^ oncoproteins not only form stable homodimers and large multiprotein complexes but also require dimerization. Nevertheless, details matter as aromatic amino acids at the deletion junction of some BRAF^Δβ3-αC^ oncoproteins, e.g., BRAF^ΔLNVTAP>F^, increase their stability and dimerization propensity while conferring resistance to monomer-favoring RAFi such as dabrafenib or HSP 90/CDC37 inhibition. In contrast, dimer-favoring inhibitors such as naporafenib inhibit all BRAF^Δβ3-αC^ mutants in cell lines and patient-derived organoids, suggesting that tumors driven by such oncoproteins are vulnerable to these compounds.

## Teaser

Aromatic amino acid insertions define the properties of BRAF^Δβ3-αC^ mutants.

## INTRODUCTION

**Fig. 1.**
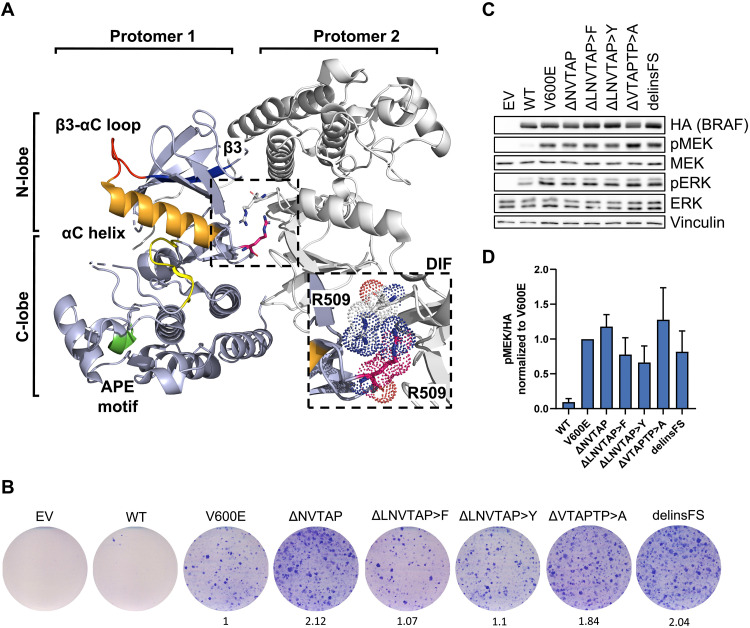
The recently identified Δβ3-αC mutation BRAF^delinsFS^ is activating and confers transforming ability. (**A**) BRAF elements that are essential in this study are highlighted in protomer 1 of a representative BRAF dimer. Orange, αC helix; red, αC-β3 loop; blue, β3 sheet; pink, R509; green, APE motif; yellow, D^594^FGLATV^600^KS motif of the AS. The ribbon diagram, which is based on a crystal structure of dimerized human BRAF kinase domains bound to 14-3-3 proteins [PDB: 6XAG, (*113*)], was created using the PyMol Molecular Graphics System (version 2.5.2, Schrödinger, LLC). The 14-3-3 dimer was excluded for simplicity. (**B**) Focus formation assay. MEFs were infected with retroviral vectors encoding the indicated BRAF proteins, cultured for 14 days, and stained with Giemsa reagent. To quantify focus formation, the integrated pixel density was determined using ImageJ and normalized to BRAF^V600E^. (**C**) Immunoblot of HEK293T cells expressing the indicated BRAF proteins. (**D**) Immunoblots were quantified using ImageJ. The bar graph shows pMEK/hemagglutinin (HA) levels (means + SD, *n* = 3) normalized to BRAF^V600E^. Images are representative of at least three independent experiments.

The serine/threonine kinases of the RAF family comprise the ARAF, BRAF, and RAF1 isoforms and represent critical signaling elements in the RAS/RAF/mitogen-activated protein kinase (MAPK) kinase (MEK)/extracellular signal–regulated kinase (ERK) pathway. RAFs, in particular the frequently mutated BRAF isoform, emerged as major drug targets in oncology (*1*). RAF becomes activated by RAS-mediated membrane recruitment, which in turn promotes the transition from a closed autoinhibited to an open conformation in which the exposed kinase domains are activated by dimerization-induced allosteric transactivation (*2*, *3*). The mechanisms leading to physiological and oncogenic RAF activation are best understood from a structural perspective (*4*–*6*). RAFs share three conserved regions (CRs): CR1 and CR2 mediate RAS and 14-3-3 binding, respectively, thereby controlling membrane recruitment and the degree of autoinhibition (*4*, *7*). Among other features, the CR3 encompasses the kinase domain, which displays the typical organization of an N- and C-lobe. The kinase domain contains a dimer interface (DIF), which comprises several noncontiguous residues in both lobes (*8*). Of these, R509, which is located in the conserved R^506^KTR^509^HV motif at the C-terminal end of the αC helix, not only plays a key role in the formation and stabilization of RAF dimers (Fig. 1A) but also is essential for the allosteric transactivation of a still inactive receiver protomer by an already activated RAF protein (*9*–*12*). The binding of 14-3-3 proteins to the C-terminal end of CR3 also contributes to dimerization (*13*).

RAS recruits BRAF to the membrane and assists in its full activation by relieving the kinase domain from 14-3-3–mediated autoinhibition (*14*). The exposed kinase domain engages in homo- or heterodimerization with other RAF protomers, which in turn induces their conformational changes (*3*, *12*, *15*) that are probably accompanied by phosphorylation of the D^594^FGLAT^599^V^600^KS^602^ motif within the activation segment (AS) (*16*). While AS phosphorylation has not been observed in recent mass spectrometry (MS) experiments (*6*), it is supported by structural and genetic approaches (*12*, *17*, *18*). For example, T599 phosphorylation is mimicked by the most common oncogenic BRAF mutation, V600E, which generates a mutation-specific salt bridge between E600 and K507 in the αC helix of the N-lobe, which in turn shifts the αC helix and AS into the active “αC helix-IN/DFG-IN/R506-IN” conformation (*17*, *19*, *20*). Consequently, this salt bridge locks BRAF^V600E^ in the active conformation and exempts it from multiple regulatory requirements, including an intact RAS-binding domain, DIF, AS, and the C-terminal 14-3-3–binding site (*9*, *11*, *18*, *21*). The fact that BRAF^V600E^ can still provide high and transforming ERK activity in the presence of artificial DIF and/or C-terminal 14-3-3–binding site mutations suggests that it signals as a monomer, and indeed, it could be shown that it is only receptive to V600E-selective type I^1/2^ RAF inhibitors (RAFi) in its monomeric state due to the phenomenon of negative allostery (*20*, *22*). However, these findings do not indicate that BRAF^V600E^ always exists as a monomer in living cells. We and others showed that BRAF^V600E^ displays a higher dimerization propensity than wild-type (WT) BRAF (BRAF^WT^) and is more effective in phosphorylating MEK in its dimeric state and that a large fraction of this oncoprotein resides in large protein complexes that are sensitive to DIF mutations (*9*, *17*, *23*, *24*).

The phosphorylation- and dimerization-induced conformational changes within the kinase domain also promote its transition from an inactive to an active conformation, involving the realignment of conserved hydrophobic regulatory residues. If they are aligned in the active conformation, they will constitute the so-called R-spine that is essential for catalysis. The spine residues provide critical contact points for RAFi, and hence, their orientation, along with that of the αC helix and the AS, decides about drug binding and efficacy (*12*, *25*). For example, the clinically irrelevant type I inhibitors stabilize the RAF kinase domain in its active αC helix-in/DFG-in/R506in conformation, while type II compounds, such as the approved sorafenib and the clinical phase 2 trialed naporafenib, stabilize the αC helix-in/DFG-out/R506in conformation. The clinically used BRAF^V600E^-selective drugs vemurafenib, dabrafenib, and encorafenib represent the aforementioned type I^1/2^ inhibitors, inducing an αC-helix-out/DFG-in/R506in conformation (*20*).

The spectrum of *BRAF* alterations is still expanding because of the increasing sequence coverage of tumor genomes. Oncogenic mutations are subdivided into single-nucleotide/amino acid substitutions (e.g., V600E), small in-frame insertions/deletions resulting in full-length BRAF proteins with altered kinase activity, and gene fusions (*26*). Their complexity is increased by the fact that BRAF oncoproteins differ in their enzymatic activity and drive MEK/ERK hyperactivation by various mechanisms (*27*). These differences have practical implications for targeted therapies and stimulated the classification of BRAF oncoproteins (*26*). Class I mutants are confined to V600 substitutions and can still unfold their high intrinsic enzymatic activity and oncogenic signaling potential if deprived of the aforementioned dimer-promoting features. In contrast, class III mutants represent the other end of the spectrum as they display lower intrinsic kinase activity than BRAF^WT^ or lack kinase activity at all. They cooperate with activated RAS and induce paradoxical MEK hyperactivation by dimerizing with catalytically competent RAF protomers and promoting their transactivation (*9*, *28*, *29*). Class II contains a wide spectrum of BRAF oncoproteins with varying degrees of intermediate activity (*30*). They rely on dimerization but can signal independent of RAS (*21*).

The so-called BRAF^Δβ3-αC^ mutants represent still relatively underexplored but potentially highly active oncoproteins found in various tumor entities, especially in *KRAS* WT pancreatic neoplasia (*31*–*34*). According to the Catalogue Of Somatic Mutations In Cancer (COSMIC) database, 0.005% of its curated pan-cancer samples encode BRAF^Δβ3-αC^ mutants. As the responsible mutations map to exon 12, which is ignored by most diagnostic procedures that only address exons 11 and 15, their frequency is probably underestimated, in particular for “*RAS/BRAF* WT” tumors of typically RAS/ERK pathway–driven entities. At the protein level, BRAF^Δβ3-αC^ mutants are characterized by short in-frame deletions removing usually five amino acids in the loop linking the β3 strand with the αC helix (*35*–*37*). As this deletion affects the orientation of the αC helix (Fig. 1A), which in turn controls the exposure of the R^506^KTR^509^HV motif, BRAF^Δβ3-αC^ mutants might display an aberrant dimerization behavior. However, the first studies describing BRAF^Δβ3-αC^ mutants arrived at different conclusions whether they signal as dimers or autonomous monomers (*35*–*37*). However, defining the biochemical properties of BRAF^Δβ3-αC^ mutants, which determine RAFi efficacy, is of direct clinical relevance, as Molecular Tumor Boards (MTBs) increasingly discuss the druggability of these oncoproteins in clinical decision-making. Here, we provide an in-depth analysis of the signaling potential and dimerization state of various BRAF^Δβ3-αC^ oncoproteins, including the previously uncharacterized BRAF^ΔLNVTAP>F^ oncoprotein and the hitherto undescribed BRAF^delinsFS^ and BRAF^ΔLNVT>F^ mutants. By defining their druggability, we observed an unexpected variety in dabrafenib responsiveness, while sorafenib and the phase 2 trialed compound naporafenib inhibit all mutants tested. We also dissect the mechanism determining dabrafenib sensitivity and propose an algorithm for choosing the appropriate RAFi in the clinical setting.

## RESULTS

### Identification of the previously unidentified in-frame deletion mutant BRAF^delinsFS^

This study was prompted by a pancreatic ductal adenocarcinoma (PDAC) case analyzed within the Molecularly Aided Stratification for Tumor Eradication Research (MASTER) program of the National Center for Tumor Diseases (NCT) and the German Cancer Consortium (DKTK) (*38*) in which a BRAF exon 12 p.L485-P490delinsFS (BRAF^delinsFS^) was detected. The patient was diagnosed with poorly differentiated PDAC and hepatic metastases at the age of 58. Palliative chemotherapy with mFOLFIRINOX [oxaliplatin, leucovorin, irinotecan, and 5-fluorouracil (5-FU)] resulted in an objective response and was deescalated to 5-FU after 7 months. At disease progression 4 months later, treatment was changed to irinotecan/5-FU [time to progression (TTP), 6 months]. Further treatment lines were nab-paclitaxel/gemcitabine (TTP, 7 months), nal-irinotecan (TTP, 9 months), and FOLFOX4 (TTP, 2 months). The patient was then enrolled in NCT/DKTK MASTER, and treatment was switched to gemcitabine/erlotinib (TTP, 3 months), which was continued beyond progression due to reduced tumor growth compared to previous regimens and a lack of therapeutic alternatives. On the basis of the BRAF^delinsFS^ mutation detected, MEK inhibition ± RAFi was recommended by the MTB. Unfortunately, no suitable clinical trial was available at that time, and the patient died 3 months later at the age of 62.

BRAF^delinsFS^ lacks six of the original amino acids of the β3-αC helix loop but carries two de novo–introduced residues, a phenylalanine and a serine, in this segment (Fig. 1A). As this represents a net deletion of four amino acids, as compared to the previously published Δβ3-αC mutants, and because deletion length influences signaling activity (*36*, *37*), we analyzed the properties of BRAF^delinsFS^. First, we compared the transformation potential of the previously uncharacterized BRAF^delinsFS^ mutant with that of other Δβ3-αC mutants (described in fig. S1) and BRAF^V600E^ in immortalized murine embryonic fibroblasts (MEFs) (Fig. 1B). BRAF^delinsFS^ induces foci to a similar extent as the other Δβ3-αC mutants, including the previously described but functionally uncharacterized BRAF^ΔLNVTAP>F^ (*39*). Commensurate with their transformation potential, all mutants activated the ERK pathway in human embryonic kidney (HEK) 293T cells (Fig. 1, C and D). The MEK/ERK phosphorylation potential of BRAF^Δβ3-αC^ mutants was not affected by the AVKA mutation replacing T599 and S602 by alanine residues (fig. S1B). This is reminiscent of BRAF^V600E^, which, unlike other BRAF oncoproteins, signals independent of an intact T^599^V^600^KS^602^ motif (*18*, *40*, *41*), indicating that AS-induced conformational changes are also dispensable for BRAF^Δβ3-αC^ oncoproteins.

### BRAF^Δβ3-αC^ mutants require dimerization for oncogenic signaling and stability

**Fig. 2.**
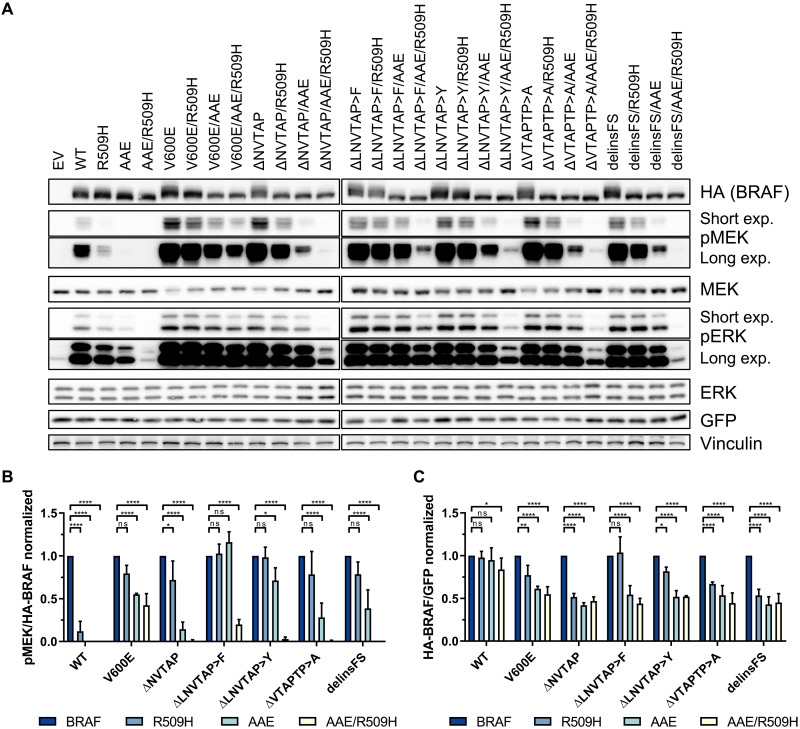
Dimerization is essential for the activity and stability of BRAF^Δβ3-αC^ mutants. (**A**) HEK293T cells were transiently transfected with the indicated HA-BRAF plasmids. Total cell lysates (TCLs) were analyzed by immunoblot using the indicated antibodies. Vinculin detection serves as a loading control. Images are representative of three independent experiments. (**B** and **C**) Immunoblots were quantified using ImageJ. Data were normalized to the corresponding BRAF protein without the additional dimerization-impairing mutations R509H and/or AAE. Statistical analysis: means + SD, *n* = 3, two-way analysis of variance (ANOVA) with Dunnett‘s test for multiple comparisons, *P ≤ 0.05, **P ≤ 0.01, ***P ≤ 0.001, ****P ≤ 0.0001. ns, not significant.

The initial studies disagreed whether BRAF^Δβ3-αC^ act as mono- or dimers and whether they require an intact DIF to unfold their oncogenic potential (*24*, *35*, *36*). This discrepancy could be explained by the fact that these laboratories studied different BRAF^Δβ3-αC^ mutants (fig. S1A). Therefore, we assessed the dimerization capacity of BRAF^delinsFS^, which formed heterodimers with RAF1 and displayed increased homodimerization potential with coexpressed BRAF^WT^ and even more pronounced with itself (fig. S1C). This finding and the aforementioned controversy about the requirements of BRAF^Δβ3-αC^ mutants for an intact DIF prompted us to systematically analyze the effects of the R509H and AAE mutations, either singly or in combination, on the signaling potential of these oncoproteins (Fig. 2). The typical DIF mutation, R509H, impairs BRAF homo- and, albeit to a lesser extent, heterodimerization (*9*). The AAE mutation was inspired by the noncanonical APE motif (AAE) at the C-terminal end of the ARAF AS that indirectly decreases the dimerization propensity of RAF kinases (*24*). We included BRAF^V600E^ as a reference for a BRAF oncoprotein that can signal and transform independent of an intact DIF (*9*, *11*, *24*, *42*). Commensurate with previous findings (*9*, *24*), the R509H and AAE substitutions had a strong and severe impact on the MEK phosphorylation potential of BRAF^WT^, respectively. In contrast, BRAF^V600E^ was less affected and only the simultaneous introduction of the R509H and AAE mutations reduced the MEK phosphorylation potential by more than 50% (Fig. 2B). Likewise, the BRAF^Δβ3-αC^ mutants resembled BRAF^V600E^ as they remained highly and moderately active in the presence of the R509H and AAE alterations, respectively. Only their combination reduced the MEK phosphorylation potential of BRAF^V600E^ by more than 70%. Unexpectedly, the BRAF^Δβ3-αC^ mutants differed in their sensitivity toward the R509H and AAE mutations, with BRAF^ΔLNVTAP>F^ and BRAF^ΔLNVTAP>Y^ being most resistant (Fig. 2, A and B).

We also assessed the transformation potential of the BRAF^Δβ3-αC^ mutants in simian virus 40 large T antigen immortalized murine fibroblasts (MEFs) and compared it with that of BRAF^V600E^ and BRAF^F595L^, another oncoprotein with lower intrinsic kinase activity than BRAF^V600E^ (*43*) but potent focus formation potential (*44*). These MEFs display a stringent contact inhibition response that is only overridden by oncogenic but not WT BRAF (*18*, *40*, *44*). As seen in Fig. 1B and fig. S2A, all BRAF^Δβ3-αC^ mutants caused focus formation to a similar extent as the high-intensity BRAF^V600E^ mutant, and hence, their transforming activity correlates with their MEK phosphorylation potential. However, introducing the R509H and AAE mutations, either singly or in combination, increased the focus formation of MEFs transformed by BRAF^Δβ3-αC^ mutants, albeit this effect was influenced by the individual in-frame deletion. At first glance, this observation appears counterintuitive but ties in with our previous observation that BRAF oncoproteins with an intermediate activity, e.g., BRAF^F595L^, are more effective in driving the proliferation of these MEFs than BRAF^V600E^ (*18*, *44*). Thus, although all cells expressing BRAF^V600E^ and the BRAF^Δβ3-αC^ mutants displayed a transformed morphology (fig. S2B), the correlation between MEK/ERK phosphorylation and focus proliferation follows a bell-shape curve (fig. S2C).

The R509H and AAE mutations increased the electrophoretic mobility of all BRAF proteins, which probably reflects their decreased phosphorylation status due to reduced feedback and transphosphorylation events (*45*). On closer inspection of the BRAF bands on well-resolved Western blots (Fig. 2A), we noticed that R509H and, in particular, AAE reduced the amount of the BRAF proteins. This apparent reduction was not caused by differences in phosphorylation status that might interfere with protein transfer or detection, as dephosphorylation of BRAF^ΔNVTAP^ did not increase its abundance (fig. S3). To distinguish between an effect on BRAF stability and abundance differences caused by distinct transfection efficiencies or transcript production/stability, we exploited the bi-cistronic design of the hemagglutinin (HA)–BRAF–internal ribosomal entry site (IRES)–green fluorescent protein (GFP) cassette of the pMIG vectors from which HA-BRAF and GFP are coexpressed (*9*). The quantitative assessment of the HA-BRAF/GFP ratio confirmed that BRAF^WT^ levels were hardly affected by dimerization-impairing mutations (Fig. 2C). R509H had little to no effects on the abundance of the high-activity BRAF^V600E^ class I mutant, the intermediate-activity class II mutant BRAF^F595L^ (*44*), BRAF^ΔLNVTAP>F^, and BRAF^ΔLNVTAP>Y^, while that of the other BRAF^Δβ3-αC^ mutants was reduced by 30 to 50% (Fig. 2, A and C, and fig. S4, A and C). Linear regression between GFP-normalized HA-BRAF expression and HA-BRAF–normalized pMEK levels upon R509H introduction revealed that, in contrast to the class II mutant BRAF^F595L^, the stability of BRAF^Δβ3-αC^ mutants, as reflected by their abundance, correlates with their MEK phosphorylation potential (fig. S4, B to D). It should be noted that the effects of the R509H and AAE mutations on the stability of BRAF^Δβ3-αC^ mutants were neither quantified nor remarked in the initial publications (*24*, *35*, *36*). Upon densitometry of the Western blot bands in these three publications, however, we noted that all three studies showed that the R509H mutation reduced the abundance of all BRAF^Δβ3-αC^ mutants to a similar extent (fig. S4E).

### BRAF^Δβ3-αC^ mutants display high dimerization propensity and form large multiprotein complexes containing heat shock protein 90

**Fig. 3.**
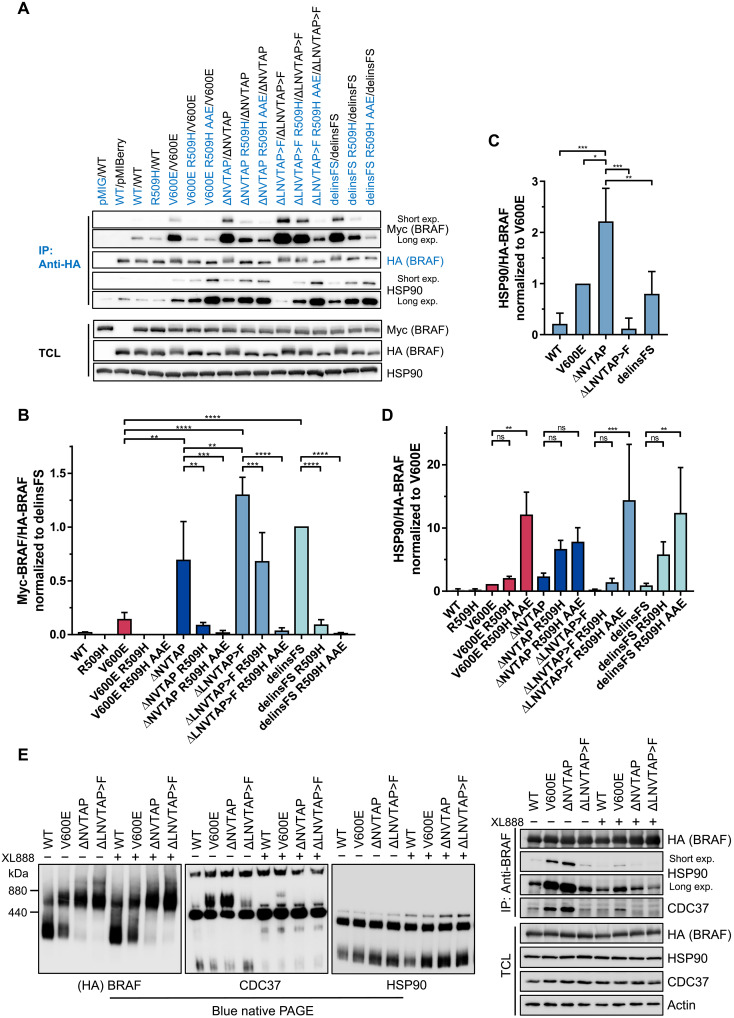
BRAF^Δβ3-αC^ mutants form exceptionally stable dimers, with varying sensitivities to the R509H mutation and affinity toward each other and heat shock protein 90. (**A**) The indicated HA- or Myc-tagged BRAF proteins were coexpressed in HEK293T cells. BRAF complexes were precipitated with an anti-HA antibody. Immunoprecipitates and TCLs were analyzed by immunoblot using the indicated antibodies. TCLs confirm the expression of heat shock protein 90 (HSP90) and the BRAF proteins in question for all coimmunoprecipitations. Images are representative of three independent experiments. IP, immunoprecipitation. (**B** to **D**) Immunoblots were quantified using ImageJ. Bar graphs show copurified Myc-BRAF (B) or HSP90 [(C) and (D)] per precipitated HA-BRAF. Statistical analysis: means + SD, *n* = 3, one-way (C) or two-way [(B) and (D)] ANOVA with Tukey’s [(B) and (C)] or Dunnett’s (D) test for multiple comparisons, *P ≤ 0.05, **P ≤ 0.01, ***P ≤ 0.001, ****P ≤ 0.0001. (**E**) Analysis of BRAF complexes by BN-PAGE. HEK293T cells expressing the indicated BRAF proteins were cultured in the presence of the HSP90 inhibitor XL888 (1 μM) or vehicle control for 4 hours, followed by lysis. TCLs were analyzed by Western blotting following BN-PAGE (left), using the indicated antibodies. To confirm the inhibition of HSP90 binding, BRAF complexes were precipitated using an anti-HA antibody, followed by SDS-PAGE and Western blotting. TCLs confirm comparable expression levels of CDC37, HSP90, and the BRAF proteins in question for coimmunoprecipitation and BN-PAGE. Representative images for two biological replicates are shown. See also fig. S5B.

Given the profound effect of dimer impairing mutations on the activity and stability of BRAF^Δβ3-αC^ mutants, we next analyzed their homodimerization potential (Fig. 3, A and B). This experimental setup in which the BRAF dimers are purified by anti-HA immunoprecipitation reveals stable dimers and can discriminate the various affinities displayed by BRAF mutants. Using this assay, we, and subsequently others applying different methods, demonstrated that BRAF^V600E^, despite its ability to signal as a monomer under artificial circumstances, has a higher homodimerization propensity than BRAF^WT^ (*9*, *17*, *46*). Unexpectedly, all BRAF^Δβ3-αC^ mutants displayed an even higher and significantly elevated homodimerization potential compared to BRAF^V600E^ (Fig. 3, A and B). Nevertheless, homodimerization was reduced but not abolished by the R509H mutation, whereas combination of R509H and AAE mutations abrogated the homodimerization potential of all analyzed mutants. The BRAF^ΔLNVTAP>F^ oncoprotein, whose MEK/ERK phosphorylation potential was the least affected by the R509H substitution, still retained more than fivefold homodimerization capacity over BRAF^V600E^ after introducing this DIF mutation.

Almost all high-activity BRAF point mutants, except for a few variants such as the highly dimerization-proficient BRAF^E585K^ oncoprotein, require the heat shock protein 90 (HSP90)/CDC37 chaperone complex for their activity (*47*, *48*). In addition, BRAF^V600E^ forms large multiprotein complexes with this chaperone (*23*). As the requirement and affinity of BRAF^Δβ3-αC^ mutants for HSP90 remains unknown, we assessed the HSP90 levels in immunoprecipitates and observed increased HSP90 binding compared to BRAF^WT^, albeit to a different extent (Fig. 3, A, C, and D). The only exception is BRAF^ΔLNVTAP>F^, which exhibits no significant difference in HSP90 recruitment compared to BRAF^WT^. The dimerization impairing R509H and AAE mutations further increased the interaction between HSP90 and the various BRAF^Δβ3-αC^ mutants. Nonlinear regression of precipitated BRAF^Δβ3-αC^ mutants and HSP90 revealed a negative correlation among BRAF^Δβ3-αC^ mutants between homodimerization and HSP90 binding (fig. S5A), suggesting that dimerization and HSP90 binding cooperate and potentially compensate each other in stabilizing BRAF^Δβ3-αC^ mutants. Using blue native polyacrylamide gel electrophoresis (BN-PAGE) and size exclusion chromatography–based proteomics, we demonstrated previously that hyperactive and dimeric BRAF^V600E^ predominantly occurs in a large multiprotein complex enriched with HSP90 and its co-chaperone CDC37, while BRAF^WT^ is mostly confined to a small complex (*9*, *23*). Therefore, we applied BN-PAGE to compare the sizes of multiprotein complexes containing either BRAF^WT^, BRAF^V600E^, BRAF^ΔNVTAP^, or BRAF^ΔLNVTAP>F^. This analysis revealed that the propensity of BRAF^V600E^ to form a large multiprotein complex was even further enhanced in both BRAF^Δβ3-αC^ mutants, as the small complex almost completely disappeared in these samples (Fig. 3E and fig. S5B). This effect was most pronounced in lysates from cells expressing BRAF^ΔLNVTAP>F^, which displayed a particularly large complex of >880 kDa. Albeit to a lesser extent, this complex was also observed in lysates from BRAF^ΔNVTAP^ but not detected in those expressing BRAF^V600E^ or BRAF^WT^. The increasing abundance in large BRAF complexes and the emergence of the >880 kDa complex correlate with the strongly increased dimerization potential of both BRAF^Δβ3-αC^ mutants compared to BRAF^V600E^ (Fig. 3B). Given the unexpected finding that BRAF^ΔLNVTAP>F^, unlike the other BRAF^Δβ3-αC^ mutants, did not differ from BRAF^WT^ in terms of HSP90 recruitment (Fig. 3, A, C, and D), we analyzed the colocalization between BRAF, CDC37, and HSP90 in BN-PAGE experiments and the association of the three proteins by coimmunoprecipitation (Fig. 3E). CDC37, which recruits kinases to HSP90, was enriched in large complexes comigrating with that of BRAF^V600E^ and BRAF^ΔNVTAP^, and this colocalization was almost abolished by the clinically tested HSP90 inhibitor XL888 (*49*). As expected from the coimmunoprecipitation experiments shown in Fig. 3 (A, C, and D), CDC37 was less abundant in large complexes comigrating with those organized by BRAF^ΔLNVTAP>F^. Unfortunately, we could not identify an HSP90 complex that comigrated with the large BRAF-containing complexes in our BN-PAGE experiments. We assume that the epitope for the anti-HSP90 antibody is not accessible in native complexes because HSP90 and CDC37 were readily detected as XL888-sensitive interactors in SDS-PAGE–resolved and, hence, denatured BRAF^V600E^ and BRAF^ΔNVTAP^ coimmunoprecipitates from this experimental setup (Fig. 3E).

Together, our BN-PAGE and coimmunoprecipitation experiments indicate that the CDC37/HSP90 complex is present in the large molecular mass complexes typically formed by BRAF^V600E^ and BRAF^∆NVTAP^ but not BRAF^∆LNVTAP>F^. Our data also show that BRAF oncoproteins do not form one but multiple high molecular mass complexes and that the >880 kDa complex observed predominantly in BRAF^∆LNVTAP>F^-expressing cells predicts a multiprotein assembly independent of HSP90/CDC37.

### Vulnerability of BRAF^Δβ3-αC^ mutants toward HSP90 inhibition correlates with their dimerization propensity

The reduced abundance of BRAF^Δβ3-αC^ mutants could be linked to an inherent instability that is compensated by increased homodimerization and/or HSP90 binding. To address these hypotheses, we generated *Braf*-deficient MEFs harboring tetracycline (tet)–regulated expression vectors for the BRAF proteins in question to monitor their longevity following tet washout (fig. S6). BRAF^WT^, its R509H/AAE counterpart, and BRAF^ΔLNVTAP>F^ displayed longer half-lives than BRAF^ΔNVTAP^ and BRAF^V600E^ (fig. S6, A to C). Our calculated half-life of BRAF^V600E^ in MEFs was in a similar range as reported for HEK293T cells (*50*). In agreement with the reduced BRAF levels shown in Fig. 2, impairing the dimerization potential of BRAF^ΔLNVTAP>F^ by the R509H/AAE mutations reduced its half-life into the range of BRAF^ΔNVTAP^ and BRAF^V600E^ (fig. S6C).

**Fig. 4.**
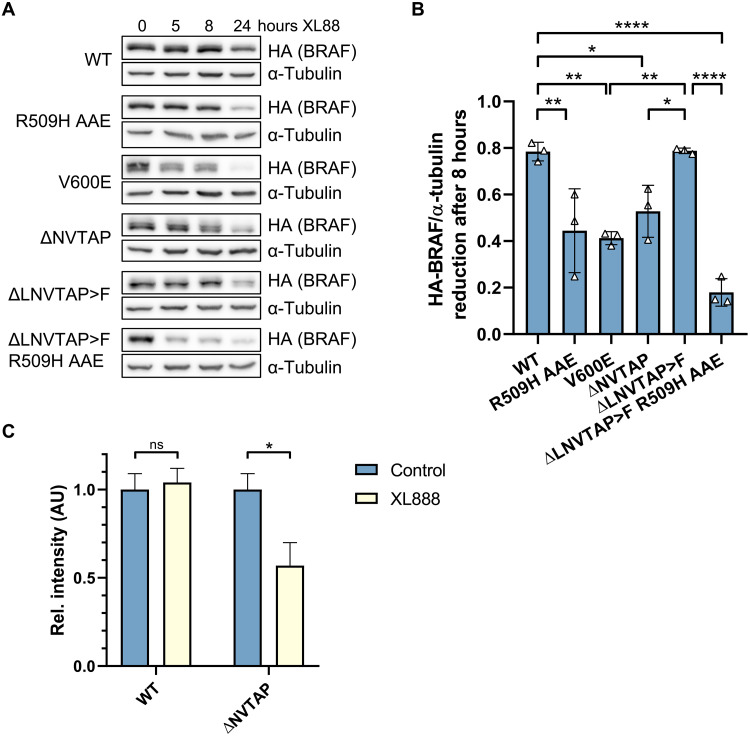
BRAF^Δβ3-αC^ mutants show enhanced susceptibility to HSP90 inhibition. (**A**) Expression of the indicated BRAF proteins in lentivirally transduced MEF lines was induced by tet addition for 72 hours, followed by addition of XL888 (1 μM). Cells were lysed after the indicated XL888 treatment periods, and HA-BRAF levels were quantified by Western blot. (**B**) BRAF levels were normalized to α-tubulin. Bar graph shows the reduction of BRAF levels after 8 hours. Statistical analysis: means + SD, *n* = 3, one-way ANOVA with Tukey’s test for multiple comparisons, *P ≤ 0.05, **P ≤ 0.01, ***P ≤ 0.001, ****P ≤ 0.0001. (**C**) Following cultivation of OV-90 cells in the presence of 1 μM XL888 or control [dimethyl sulfoxide (DMSO)] for 24 hours, endogenous BRAF was purified and digested with trypsin before MS. Abundances of the BRAF^WT^ (MLNVTAPTPQQLQAFK)– and corresponding BRAF^ΔNVTAP^ (MLTPQQLQAFK)–derived peptides were compared between control (DMSO) and XL888-treated cells. Peptide abundance for both proteins purified from DMSO-treated cells was set to 1 and was measured in technical triplicates. Statistical analysis: means + SEM, unpaired, two-tailed *t* tests, *P ≤ 0.05, **P ≤ 0.01, ***P ≤ 0.001, ****P ≤ 0.0001. AU, arbitrary units.

Next, we investigated whether XL888 would affect the stability of BRAF^Δβ3-αC^ oncoproteins (Fig. 4, A and B, and fig. S7). XL888 caused a noticeable depletion of BRAF^ΔNVTAP^ down to 50%. In contrast, BRAF^ΔLNVTAP>F^ levels were only mildly reduced at 8 hours and comparable to those of BRAF^WT^. In line with Fig. 3, increased HSP90 binding induced by reduction of dimerization potential (R509H AAE) sensitized BRAF^ΔLNVTAP>F^ and BRAF^WT^ for HSP90 inhibition. This suggests that BRAF^Δβ3-αC^ mutants are less stable and are stabilized to a different extent by increased dimerization or HSP90 binding.

To confirm the decreased stability and enhanced XL888 sensitivity of an endogenously expressed BRAF^Δβ3-αC^ mutant, we established an approach using the human ovarian carcinoma cell line OV-90 in which we can monitor the coexpression, as suggested by genomic polymerase chain reaction (PCR) (fig. S13A), and abundance of BRAF^WT^ and BRAF^ΔNVTAP^ side by side. By looking at the distribution of trypsin cleavage sites in BRAF, we reasoned not only that MS would allow us to detect a peptide specific for the ΔNVTAP deletion but also that HSP90 inhibition should trigger its depletion. The BRAF^ΔNVTAP^-derived peptide was reduced by 50% upon XL888 treatment, whereas the abundance of the BRAF^WT^ peptide was unaffected (Fig. 4C). Our MS approach might be also of diagnostic interest as it could be useful to confirm the endogenous expression of similar oncoproteins generated by short in-frame deletions/insertions, e.g., epidermal growth factor receptor (EGFR) and HER2 (*36*), which cannot easily be distinguished from their WT counterparts by Western blotting or by immunohistochemistry.

### BRAF^Δβ3-αC^ mutants differ in their sensitivity toward type I^1/2^ inhibitors but are all blocked by type II compounds

However, how could tumors with BRAF^Δβ3-αC^ mutants be treated with targeted therapy? MEK inhibitors (MEKi) would be an obvious choice as trametinib blocked ERK pathway activation by all BRAF^Δβ3-αC^ oncoproteins (fig. S8A). We also searched for a strategy directly inhibiting BRAF^Δβ3-αC^ mutants as such a RAFi could be very useful, either in a monotherapy setting or as a component of a vertical pathway inhibition strategy (*51*). In the initial studies, however, the tested BRAF^Δβ3-αC^ mutants were not blocked by the type I^1/2^ inhibitor vemurafenib, while they remained sensitive toward the type I inhibitor GDC-0879 and the type II inhibitors LY3009120 and AZ-628 (*35*, *36*). We confirmed these findings for LY30009120 and vemurafenib and extended them to other BRAF^Δβ3-αC^ mutants (fig. S8, B and C).

As the phase 1 trial of LY3009120 was terminated because of inefficacy (*52*) and GDC-0879 as well as AZ-628 have not progressed beyond preclinical testing [(*19*) and our own research on https://clinicaltrials.gov], we first analyzed the sensitivity of the highly active and dimerizing BRAF^ΔLNVTAP>F^ oncoprotein toward other type II inhibitors, including the clinically applied sorafenib and currently trialed inhibitors such as belvarafenib (*53*) and naporafenib (LXH254) (*54*). We also tested the clinically available type I^1/2^ inhibitors dabrafenib and encorafenib for their activity against BRAF^ΔLNVTAP>F^. While few data are available for dabrafenib for BRAF^ΔNVTAP^ (*35*), the activity of encorafenib against BRAF^Δβ3-αC^ mutants is unknown. Both type I^1/2^ inhibitors were ineffective against BRAF^ΔLNVTAP>F^ (fig. S8D). In contrast, all type II inhibitors impaired MEK/ERK activation by BRAF^ΔLNVTAP>F^.

**Fig. 5.**
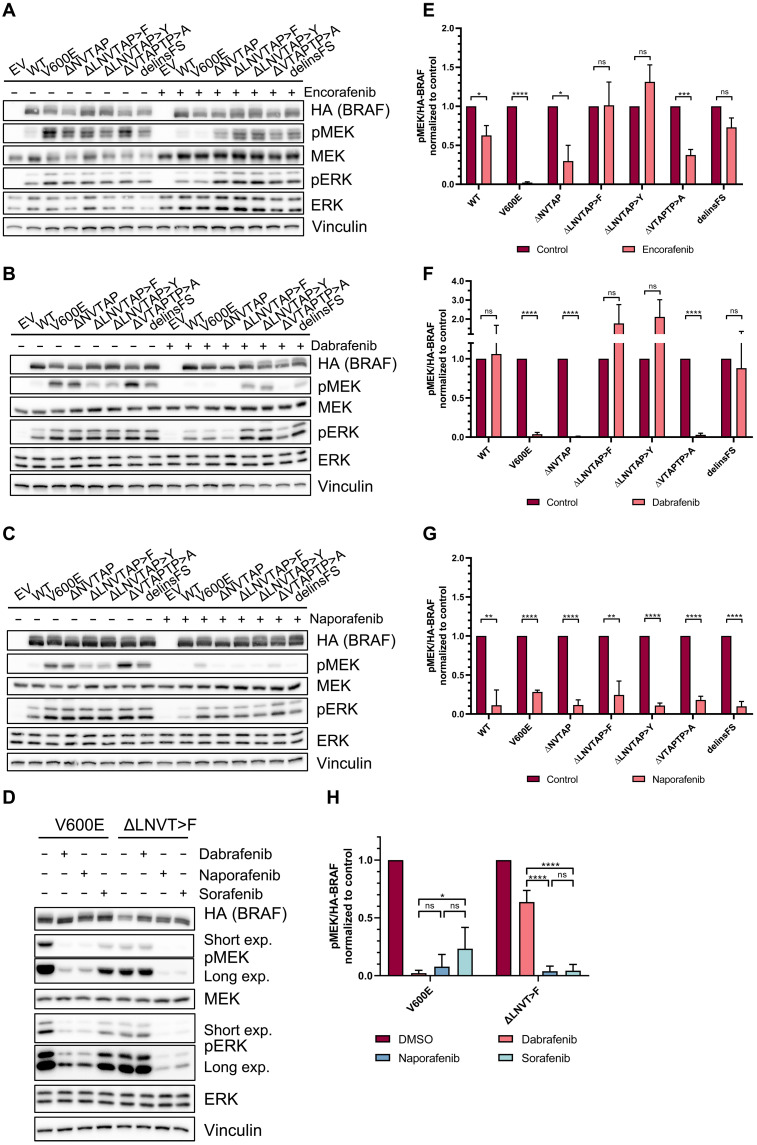
BRAF^Δβ3-αC^ mutants exhibit varying sensitivity to encorafenib and dabrafenib, but all of them are potently inhibited by dimer-targeting naporafenib. (**A** to **D**) The indicated HA-BRAF proteins were transiently expressed in HEK293T cells. Before lysis, cells were treated with encorafenib (0.5 μM), dabrafenib (1 μM), naporafenib (1 μM), sorafenib (10 μM), or vehicle control (DMSO) for 4 hours. The lysates were analyzed by immunoblot using the indicated antibodies. (**E** to **H**) Immunoblots were quantified using ImageJ. Bar graphs show calculated fold changes (inhibitor/control) of phospho-MEK levels normalized to HA-BRAF. The indicated inhibitors were compared to vehicle control (E) to (G) or each other (H) for each BRAF protein. BRAF^V600E^ served as a control. Statistical analysis: means + SD, *n* = 3, unpaired *t* tests with Holm-Šídák correction for multiple comparisons (E) to (G) or two-way ANOVA with Tukey’s test for multiple comparisons (H), *P ≤ 0.05, **P ≤ 0.01, ***P ≤ 0.001, ****P ≤ 0.0001. Quantified phospho-ERK levels are shown in fig. S9.

Given the poor sensitivity of BRAF^Δβ3-αC^ mutants toward vemurafenib (fig. S8C), the clinical availability of dabrafenib and encorafenib, and their distinct effects on kinase domain conformation (*19*), we compared the sensitivity of additional in-frame deletion mutants to these type I^1/2^ inhibitors and naporafenib. HEK293T cells expressing BRAF^V600E^ served as reference for successful inhibition by dabrafenib and encorafenib. While encorafenib was quite ineffective in reducing MEK phosphorylation triggered by all BRAF^Δβ3-αC^ mutants, dabrafenib inhibited BRAF^ΔNVTAP^ and BRAF^ΔVTAPTP>A^ but not BRAF^ΔLNVTAP>F^, BRAF^ΔLNVTAP>Y^, and BRAF^delinsFS^. Notably, we rather observed a trend for increased MEK phosphorylation in cells expressing BRAF^ΔLNVTAP>F^ and BRAF^ΔLNVTAP>Y^ treated with these type I^1/2^ inhibitors (Fig. 5, A, B, E, and F). In contrast, the type II inhibitor naporafenib was effective against all BRAF^Δβ3-αC^ mutants (Fig. 5, C to G). As often observed in these experiments and probably reflecting the multiple feedback loops and rheostasis mechanisms operating in the RAS/ERK pathway (*40*, *55*–*58*), the RAFi-mediated effects were more pronounced at the level of MEK than ERK phosphorylation. Nevertheless, pERK levels followed similar trends (fig. S9).

Given the contrasting efficacies of type I^1/2^ compounds against the various BRAF^Δβ3-αC^ mutants, we next assessed their affinity in a cellular thermal shift assay (CETSA) in which drugs stabilize their target against heat-induced denaturation in cellulo (*59*). Thermal stability of the dabrafenib-sensitive mutants BRAF^ΔNVTAP^ and BRAF^V600E^ (positive control) was increased by dabrafenib, whereas that of the insensitive BRAF^ΔLNVTAP>F^ oncoprotein remained unaffected, indicating inefficient drug accommodation (fig. S10, A to D). Thus, CETSA confirms the suspected variation in binding efficiency of dabrafenib to BRAF^Δβ3-αC^ oncoproteins. BRAF^ΔLNVTAP>F^ activity, monitored via phospho-MEK levels (fig. S10E), was eventually inhibited in the presence of 100 μM dabrafenib, an exceptionally high concentration not achievable in a therapeutic setting, suggesting that drug binding to BRAF^ΔLNVTAP>F^ is not completely prevented. This impaired drug binding could explain the observed paradoxical activation in BRAF^ΔLNVTAP>F^- and BRAF^ΔLNVTAP>Y^-expressing HEK293T cells treated with encorafenib or dabrafenib (Fig. 5, A, B, D, and E). In cells expressing dabrafenib-/encorafenib-receptive mutants like BRAF^ΔNVTAP^, the applied inhibitor concentration saturated most protomers. In contrast, the same concentration is only subsaturating in cells expressing variants like BRAF^ΔLNVTAP>F^ and BRAF^ΔLNVTAP>Y^ that display a reduced binding affinity to these type I^1/2^ compounds. The few drug-bound BRAF^ΔLNVTAP>F/Y^ protomers, however, that manage to take up these inhibitors might serve, because of their high dimerization propensity (Fig. 3B), as highly potent allosteric transactivators of drug-free RAF protomers, resulting in paradoxical MEK phosphorylation (*60*, *61*). This model is supported by Yuan *et al.* (*24*), showing that other BRAF^Δβ3-αC^ oncoproteins rendered kinase-inactive by mutation serve as allosteric transactivators. Alternatively, but not excluding the first model, it might be possible that BRAF^ΔLNVTAP>F^ and BRAF^ΔLNVTAP>Y^, which are less likely occupied by type I^1/2^ compounds, are further activated by drug-bound WT BRAF or RAF1, as both isoforms take up dabrafenib and encorafenib in the single-digit nanomolar range (*62*–*64*).

Next, we asked whether the high homodimerization propensity of BRAF^ΔLNVTAP>F^ could explain its dabrafenib resistance by negative allostery (*19*, *65*) and introduced the R509H mutation, either singly or in combination with the AAE substitution, into this oncoprotein. Unexpectedly, these alterations did not restore dabrafenib sensitivity, suggesting that other mechanisms modulate dabrafenib affinity of BRAF^Δβ3-αC^ mutants (fig. S11).

In search of an explanation for the varying properties of the analyzed BRAF^Δβ3-αC^ mutants, we noticed that BRAF^ΔNVTAP^, BRAF^ΔLNVTAP>F^, and BRAF^ΔLNVTAP>Y^ only differ in the amino acid residue at position 485 (fig. S1), with BRAF^ΔLNVTAP>F^ resembling the previously described point mutation L485F (*41*). Notably, while representing a smaller net deletion, BRAF^delinsFS^ also substitutes L485 by a phenylalanine residue, and the tyrosine introduced into BRAF^ΔLNVTAP>Y^ might entertain similar hydrophobic interactions. In BRAF^L485F^, F485 has been implicated to interact with F498, thereby creating a critical hydrophobic network that contributes to increased kinase activity and resistance to type I^1/2^ inhibitors, including dabrafenib (*41*, *66*). As suggested by structural models of BRAF^ΔNVTAP^ and BRAF^ΔLNVTAP>F^, this aromatic interaction could also be established in BRAF^Δβ3-αC^ variants exhibiting an aromatic amino acid residue at position 485 (fig. S12A). Therefore, we tested whether replacing F498 by an alanine residue could abrogate the differences between BRAF^Δβ3-αC^ mutants (fig. S12, B and C). Unexpectedly, the F498A substitution strongly reduced the activity of BRAF^ΔNVTAP^, although the proposed aromatic interaction of F498 cannot be established in this mutant as L485 remains preserved (fig. S12A). In addition, the moderate reduction of BRAF^V600E^ activity upon F498A introduction suggests a broader and hitherto unrecognized role of F498 in BRAF activity extending beyond the previously proposed interaction with L485F. In contrast to BRAF^ΔNVTAP^, pMEK levels of BRAF^ΔLNVTAP>F^ and BRAF^delinsFS^ were only mildly reduced, suggesting that the de novo–inserted aromatic amino acid residue of BRAF^ΔLNVTAP>F^ or BRAF^delinsFS^ could compensate for the loss of F498. In agreement with the model postulating an aromatic F485-F498 interaction (*41*, *66*), the F498A substitution reduced the intrinsic dabrafenib resistance of BRAF^ΔLNVTAP>F^, albeit by only 50% (fig. S12, D and E). While our manuscript was in initial review, we identified a previously unidentified exon 12 in-frame deletion mutant, BRAF^ΔLNVT>F^, in a melanoma case. This mutant provides an independent conformation for our hypothesis that aromatic amino acid residue substitutions of L485 play a central role in rendering BRAF^Δβ3-αC^ mutants resistant to type I^1/2^ inhibitors. BRAF^ΔLNVT>F^ differs from the previously characterized type^1/2^ inhibitor–resistant mutants by its shorter net deletion of three amino acids (fig. S1). However, L485 was also substituted with a phenylalanine residue. As predicted from our analyses on BRAF^Δβ3-αC^ variants with aromatic de novo amino acid insertions at position 485, BRAF^ΔLNVT>F^ was insensitive to dabrafenib. In contrast, naporafenib and sorafenib efficiently blocked the signaling output of BRAF^ΔLNVT>F^ (Fig. 5, D and H). In summary, all four BRAF^Δβ3-αC^ variants with aromatic de novo amino acid insertions show intrinsic dabrafenib resistance.

### Naporafenib blocks the proliferation of human cell lines expressing endogenous BRAF^Δβ3-αC^ oncoproteins

**Fig. 6.**
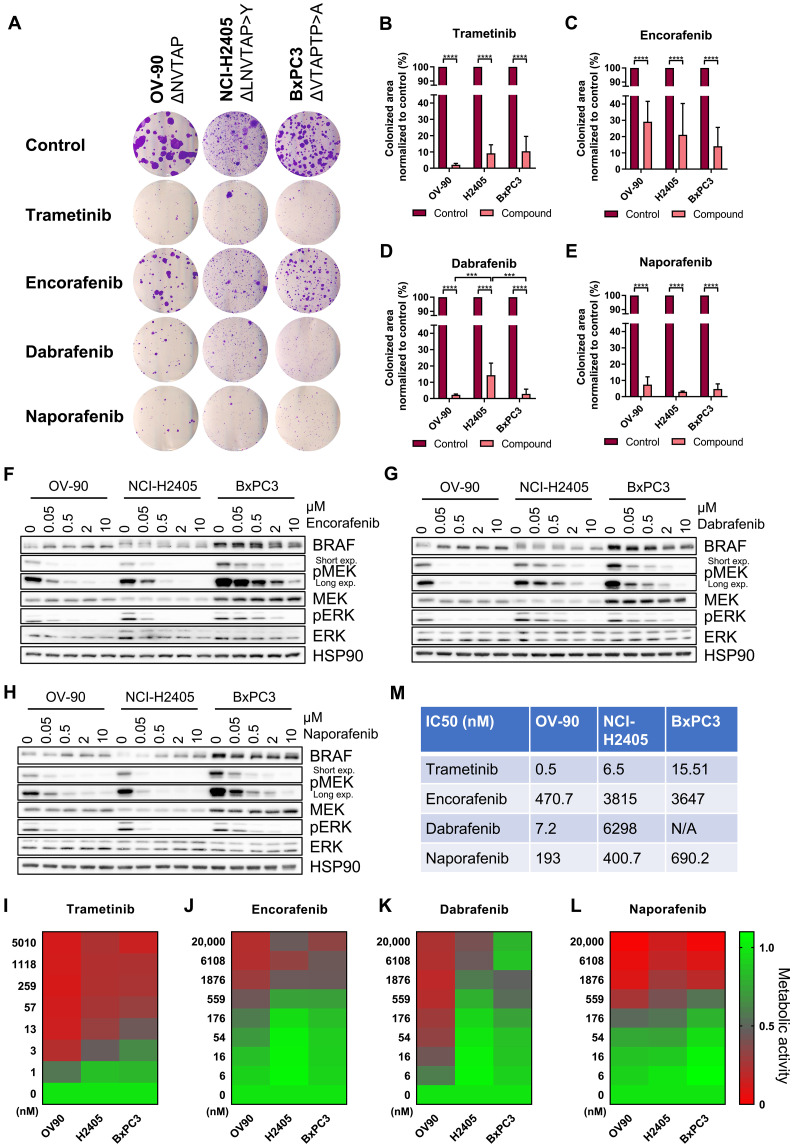
Encorafenib, dabrafenib, and naporafenib block the growth of BRAF^Δβ3-αC^ mutant–expressing cell lines with varying efficacy. (**A**) Cells were cultivated in the presence of trametinib (5 nM), encorafenib (0.5 μM), dabrafenib (1 μM), naporafenib (1 μM), or vehicle control (DMSO). OV-90, NCI-H2405, and BxPC3 cells were fixed and stained with crystal violet after 16, 21, or 18 days, respectively. (**B** to **E**) The colonized area was determined using ImageJ. Bar graphs show the colonized area of inhibitor-treated cells normalized to the area of those treated with vehicle control. Statistical analysis: means + SD, *n* = 3, two-way ANOVA with Tukey’s test for multiple comparisons, *P ≤ 0.05, **P ≤ 0.01, ***P ≤ 0.001, ****P ≤ 0.0001. (**F** to **H**) Western blot analysis showing the effect of the applied RAFis on MEK/ERK phosphorylation in the three cell lines. Detection of HSP90 serves as loading control. The corresponding quantification of pMEK levels is shown in fig. S15 (A to C). (**I** to **L**) Heatmaps showing the antiproliferative effect of trametinib, encorafenib, dabrafenib, and naporafenib on BRAF^Δβ3-αC^ mutant–expressing cancer cell lines. Following incubation with inhibitor or vehicle control for 96 hours at the indicated concentrations, the metabolic activity was measured by XTT assay and normalized to vehicle control (*n* = 3). (**M**) Antiproliferation IC_50_ values were calculated by nonlinear fitting using GraphPad Prism 9. Calculated fitted curves are shown in fig. S15 (D to G).

The well-defined heterologous HEK293T system provides a strong advantage when comparing BRAF oncoproteins for their signaling output and druggability as it allows the comparison of the various mutants in question without the interference by cell line–specific comutations—a problem that might arise when comparing multiple cell lines. A disadvantage of this approach, however, is the ectopic overexpression of the oncoprotein in question outside of its histological context. This is particularly important as the histological context, which is mainly defined by the ontogeny of the cancer cell and its tumor microenvironment, is responsible for the contrasting drug responsiveness of various BRAF^V600E^-driven tumor entities (*67*, *68*). Therefore, we assayed the drug responsiveness of three cell lines derived from ovarian (OV-90), non–small cell lung (NCI-H2405), and pancreatic (BxPC3) carcinoma that harbored three distinct endogenous BRAF^Δβ3-αC^ oncoproteins, as we confirmed ourselves (fig. S13, A to C). Again, naporafenib and, as expected from its action downstream of BRAF^Δβ3-αC^ oncoproteins, trametinib suppressed colony growth in all cell lines by more than 90% (Fig. 6, A to L). In contrast, encorafenib was less effective in all three cell lines, while the effects of dabrafenib on colony growth differed between the cell lines with the BRAF^ΔNVTAP^-expressing cell line OV-90 being the most sensitive. The high BRAF dependency of OV-90 is also reflected by the DepMap tool (https://depmap.org/portal/) that lists *BRAF* within the top 10 most essential genes for this but not the other two cell lines. Western blotting confirmed the successful but variable inhibition of the MEK/ERK pathway in all three cell lines, with the OV-90 cell line again responding best to encorafenib and dabrafenib (Fig. 6, F to H, and fig. S14, A to C). BxPC3 displayed the highest BRAF levels of these three cell lines, which agrees with the reported tetrasomy of the *BRAF*-containing chromosome 7 (*69*). As type I^1/2^ inhibitor efficacy is modulated by the expression level of *BRAF* (*70*) and the ratio between its WT and mutant versions differing in drug affinity (*63*, *64*), it should be also kept in mind that NCI-H2405 lacks a *BRAF^WT^* allele, while OV90 and BxPC3 contain *BRAF*^WT^ and *BRAF*^Δβ3-αC^ alleles (fig. S13, A to C).

As naporafenib is still awaiting clinical approval, we asked whether sorafenib, a clinically extensively used type II inhibitor that has been crystalized with BRAF^ΔNVTAP^ (*36*), would yield similar effects (fig. S13, D and E). Four and ten micromolar sorafenib significantly reduced colony growth in all cell lines with endogenous BRAF^Δβ3-αC^ mutations, and even 1 μM led to a slight but significant reduction in colony growth in OV-90 and NCI-H2405 cells. As these sorafenib concentrations are widely used in the field (*28*, *71*, *72*) and because peak plasma concentrations of up to 20 μM range were reported (*73*, *74*), our data suggest that this clinically available type II inhibitor could be further explored for the treatment of tumors driven by BRAF^Δβ3-αC^ oncoproteins.

Next, we performed metabolic 2,3-bis-(2-methoxy-4-nitro-5-sulfophenyl)-2*H*-tetrazolium-5-carboxanilid (XTT) assays to determine half-maximal inhibitory concentration (IC_50_) values for the three RAFi and trametinib in the three human cell lines with endogenously expressed BRAF^Δβ3-αC^ oncoproteins (Fig. 6, I to M, and fig. S14, D to I). Similar to the colony growth and Western blot assays, the calculated IC_50_ values and the heatmaps demonstrate the relatively uniform responses of all three human cell lines to trametinib and naporafenib, while those to the type I^1/2^ inhibitors encorafenib and dabrafenib varied considerably. Unexpectedly, we observed paradoxical metabolic activity in the pancreatic adenocarcinoma cell line BxPC3 at high dabrafenib concentrations, which precluded us from determining an IC_50_ for this cell line. As this phenomenon was not observed in the other cell lines, we exclude an artifact caused by chemical interference between dabrafenib and XTT. In addition to trametinib and the various RAFi, the ERK inhibitor ulixertinib (*75*, *76*) was similarly effective at clinically achievable concentrations in all three cell lines (fig. S14I).

Moreover, because BxPC3 expresses the BRAF^ΔVTAPTP>A^ variant, which was as efficiently inhibited as BRAF^ΔNVTAP^ by both type I^1/2^ inhibitors in the HEK293T system (Fig. 5), we expected that the antiproliferative effects on OV-90 and BxPC3 cells would be comparable. To further investigate why BxPC3 differed so drastically from OV-90, we analyzed the phosphorylation status of EGFR and AKT, as we suspected an up-regulation of metabolic processes by compensatory hyperactivation of these signaling elements, e.g., by relief from MEK/ERK-mediated negative feedbacks or cross-talk (*55*, *77*–*80*). These analyses revealed two interesting differences between the three cell lines (fig. S14J). First, OV-90 lacked the prominent expression and autophosphorylation of EGFR observed in NCI-H2405 and BxPC3 cells. Second, BxPC3 exhibited high levels of AKT phosphorylated at the activating mTORC2 phosphorylation site S473 (*81*), which was further augmented by dabrafenib or naporafenib. This up-regulation might reflect the negative cross-talk between the ERK and AKT pathways that has been described for various cell types, including BxPC3 cells (*80*, *82*). Thus, in addition to the aforementioned differences between the three BRAF^Δβ3-αC^ oncoproteins in terms of their RAFi sensitivity, differences in EGFR expression/activity and/or AKT activity could explain the increased sensitivity of OV-90 cells to the three RAFi and trametinib. Conversely, the unexpected mild-to-moderate effects of type I^1/2^ inhibitors on BxPC3 cells could be due to the high activity of the PI3K/AKT signaling axis and the relief of EGFR from negative feedback (*78*, *79*). Nevertheless, how naporafenib achieves substantial inhibition across the three cell lines (Fig. 6L), despite promoting phospho-AKT levels as well, requires further study. Collectively, these data, and, in particular, the phenotype of BxPC3 cells, demonstrate that the comparison of human cell lines with similar alterations in the pathway of interest is confounded by alterations such as co-mutations or chromosomal aberrations specific to each cell line and potentially cell-of-origin–related differences in gene expression.

**Fig. 7.**
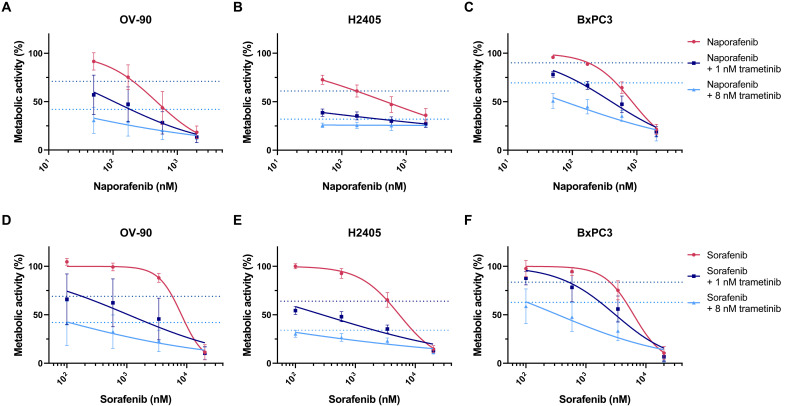
Trametinib enhances the efficacy of type II RAFi. BRAF^Δβ3-αC^ mutant–expressing cancer cell lines were incubated with naporafenib (**A** to **C**) or sorafenib (**D** to **F**) at the indicated concentrations, alone or combined with 1 or 8 nM trametinib, to explore the potential of vertical pathway inhibition. After 96 hours, the metabolic activity was measured by XTT assay and normalized to vehicle control. The metabolic activity in the absence of RAFis is indicated by dotted lines in the color of the respective trametinib concentration (dark blue, 1 nM trametinib; light blue, 8 nM trametinib). Graphs show the means + SD of three independent experiments. Nonlinear fitted curves were calculated using GraphPad Prism 9.

Given the efficacy of the type II compounds naporafenib and sorafenib across all BRAF^∆β3-αC^ oncoproteins (Figs. 5 and 6 and fig. S8D), we combined them with the MEKi trametinib, which further increased the efficacy of these RAFi at nanomolar concentrations (Fig. 7, A to F). Notably, the sorafenib/trametinib combination has already been applied in the context of BRAF class III mutations (*83*) and advanced hepatocellular carcinoma (*84*), while first clinical data on naporafenib/trametinib combinations have recently been published for NRAS-driven melanoma (*85*). We also tested whether the efficacy of naporafenib could be further improved by the HSP90i XL888, which shows clinical activity in combination with vemurafenib in melanoma (*49*, *86*). In all three cell lines, however, XL888 exhibited a narrow range between not being additive to naporafenib and too toxic by itself to discern additive/synergistic effects with this RAFi (fig. S14, K to M). In summary, our analyses support the concept that the responsiveness of human cell lines expressing BRAF^∆β3-αC^ oncoproteins toward dabrafenib and encorafenib is modulated by the details of the BRAF in-frame deletion and their cellular context. In contrast, type II inhibitors, the MEKi trametinib, and the ERKi ulixertinib all impair viability in a uniform manner. Moreover, because sorafenib and trametinib were approved more than 10 years ago and because naporafenib is currently in clinical phase 2 trials, our data highlight potential clinically realizable vertical combination therapies for BRAF^Δβ3-αC^-driven tumors.

### Confirmation of type II RAFi efficacy in patient-derived organoids

**Fig. 8.**
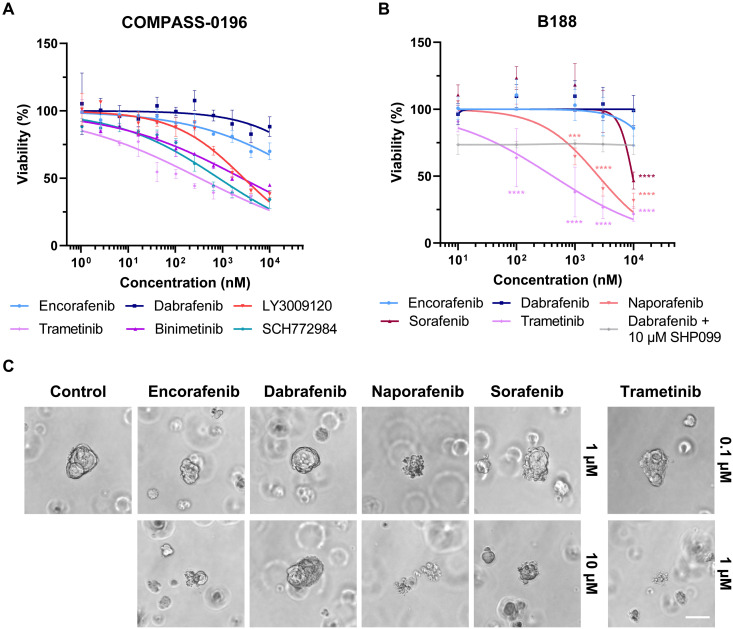
Type II RAFi like naporafenib suppresses the growth and viability of PDAC PDOs. (**A**) COMPASS-0196 PDOs were treated with the indicated kinase inhibitors for 7 days. The viability was determined by CellTiter-Glo 3D assay and normalized to vehicle control. The graph shows the means + SD and calculated fitted curves of three independent experiments. (**B**) The viability of B188 organoids grown in the presence of the indicated inhibitors for 72 hours was determined using the CellTiter-Glo 3D assay and normalized to vehicle control (DMSO). Nonlinear fitted curves were calculated using GraphPad Prism 9. Statistical analysis: means + SD, *n* = 4 (dabrafenib + SHP099: *n* = 3), two-way ANOVA with Dunnett’s test for multiple comparisons, *P ≤ 0.05, **P ≤ 0.01, ***P ≤ 0.001, ****P ≤ 0.0001. (**C**) Representative micrographs of inhibitor-treated B188 organoids right before performing the viability measurements shown in (B). Scale bar, 50 μM.

As the three cell lines investigated have been established more than two decades ago, we next screened patient-derived organoids (PDOs) or associated unpublished datasets available to us for *BRAF* exon 12 in-frame deletions. We identified two PDAC PDOs harboring *BRAF*^ΔNVTAP^ mutations and investigated their drug responsiveness. The first dataset was derived from the COMPASS-0196 (NCT-04469556) PDO that was already drug tested before we identified the efficacy of naporafenib on BRAF^Δβ3-αC^ mutants. In this PDO, the type II RAFi LY3009120, the two MEKi binimetinib and trametinib, and the ERKi SCH772984 were highly effective in suppressing PDO growth, while dabrafenib and encorafenib were only effective at very high concentrations (Fig. 8A). The underlying molecular mechanism remains unclear at present, but the presence of an ERBB3^G507R^ mutation, which is uncharacterized so far but is located in subdomain IV involved in dimerization control of this receptor tyrosine kinase (RTK), and a slight copy number variation (CNV) gain in *KRAS* (four copies) might have contributed to the paradoxical action of dabrafenib.

Given the aforementioned failure of LY3009120 in clinical trials and the data shown in Figs. 5 and 6, we generated another dataset of the B188 PDO using the type II RAFi naporafenib and sorafenib, the type I^1/2^ compounds dabrafenib and encorafenib, as well as the MEKi trametinib. Trametinib and also both type II RAFi potently suppressed the viability of the PDO at clinically achievable concentrations (Fig. 8, B and C). Similar to the COMPASS-0196 PDO, dabrafenib and also encorafenib only showed slight effects at very high concentrations. As this finding is in contrast to the dabrafenib sensitivity of BRAF^ΔNVTAP^ mutant OV-90 cells, we screened the next generation sequencing (NGS) data of the B188 PDO for potential resistance mechanisms. Notably, we identified a previously unidentified E138Q mutation in GNA13 (fig. S13C). Although this mutation represents a variant of unknown significance, RAS/ERK activation has been observed in cells overexpressing this heterotrimeric G protein subunit (*87*, *88*). Consequently, one could envisage a paradoxical action of type I^1/2^ inhibitors. Alternatively, but not excluding these possibilities, it is possible that, in contrast to the simple OV-90 culture medium, the organoid media enriched with EGF and fibroblast growth factor 10 (FGF10) could have contributed to paradoxical action of the type I^1/2^ compounds and therefore could have blunted drug responses in both PDO models. In that regard, we combined dabrafenib with an allosteric SHP2 inhibitor to lower physiological RAS signaling (Fig. 8B). Although this compound by itself lowered viability by 25%, we did not observe significant additive effects with dabrafenib. Thus, identifying these confounding factors of dabrafenib resistance represents a project beyond the current study. Nevertheless, our cell line and PDO data already show that the *BRAF* exon 12 genotype represents only one criterion for inhibitor choice. Even if a dabrafenib-sensitive mutant is encountered, the spectrum of co-mutations and/or the ground state of the signaling network, e.g., RTK expression levels, might critically modulate dabrafenib responses. This insight represents a strong encouragement for more comprehensive genomic profiling before therapy. Moreover, a switch to type II inhibitors should be considered if primary or secondary drug resistance phenomena are encountered during dabrafenib therapy (fig. S16).

## DISCUSSION

As comprehensive mutational screening of the entire *BRAF* coding sequence becomes diagnostic routine, more alterations outside of the classical hotspot exons 11 and 15 are discussed in MTBs. We noticed an increase in enquiries and literature concerning *BRAF* exon 12 alterations, most notably Δβ3-αC in-frame deletions (*31*, *32*, *34*, *39*, *89*). For example, 3.15% of *KRAS* WT PDACs analyzed in a multicenter study carried such alterations (*90*). Depending on the cohort, Chen *et al.* (*35*) reported a prevalence between 2.3 and 7.1% in *KRAS* WT pancreatic carcinoma. Considering the 458,918 new cases of pancreatic carcinoma reported in 2018 (*91*) and that ⁓5 to 10% of these lack *KRAS* alterations, we estimate that several thousand patients of this disease group alone will be potentially diagnosed with druggable BRAF^Δβ3-αC^ mutants annually. We expect that more exon 12 variants will be found in the future, and here, we extend their spectrum by identifying and characterizing two previously unidentified mutants, BRAF^delinsFS^ and BRAF^ΔLNVT>F^. The BRAF^Δβ3-αC^ mutants originally identified within human neoplasms have been recently observed in 19% of canine urothelial carcinoma (*92*), highlighting the possibility that studying their pathobiology and druggability will bridge between human and veterinarian oncology.

From our experience, two major questions are recurrently raised in MTBs regarding BRAF^Δβ3-αC^ mutants. The first concerns their general relevance as oncogenic drivers, in particular, as activity correlates with deletion length (*36*, *37*). So far, all BRAF^Δβ3-αC^ mutants turned out to be very potent oncogenic drivers and BRAF^delinsFS^ and BRAF^ΔLNVT>F^, despite their shorter deletion, are no exception. On the basis of our functional analyses (Fig. 2, A and B) and previous studies (*35*, *66*), we posit that all BRAF^Δβ3-αC^ oncoproteins owe their high signaling potential to their high dimerization propensity that stems from their αC helices locked in the IN position. In that regard, BRAF^Δβ3-αC^ oncoproteins imitate a conformation into which WT RAF proteins are transferred during dimerization. Recent structural modeling proposes that dimerization breaks protomer-internal hydrophobic interactions mediated by so-called π-π stacking and replaces them with intermolecular π-π bonds formed between aromatic amino acid residues in both protomers (*15*). These interactions reorientate the αC helix and the HRD motif, leading to kinase activation through R-spine formation (*41*, *93*). Consequently, the high dimerization propensity of BRAF^Δβ3-αC^ oncoproteins promotes full kinase activation and MEK phosphorylation, which is facilitated by dimeric RAF (*24*).

The second and even more pressing question is which targeted therapy compounds are the most appropriate for treating tumors carrying *BRAF* exon 12 in-frame deletions. Given the high activity of BRAF^Δβ3-αC^ mutants, tumors driven by these oncoproteins qualify for a treatment regimen involving MEKi, as also reflected by therapeutic responses of several PDAC cases (*31*, *34*) and a Langerhans cell histiocytosis (LCH) (*94*). In BRAF^V600E^-driven tumors, however, MEKi are usually combined with BRAF^V600E^-selective drugs to achieve more sustainable therapeutic outcomes (*95*). On the basis of pathway topology, it can be expected that this concept is also applicable to other cancers driven by highly active non-V600E BRAF mutants. Moreover, the emerging concept of low-dose vertical pathway inhibition (*51*, *96*) highlights the necessity to identify compounds effectively and directly inhibiting BRAF^Δβ3-αC^ oncoproteins for future treatment regimen. Here, we show that oncogenic signaling by all BRAF^Δβ3-αC^ mutants we investigated can be efficiently inhibited by the type II RAFi naporafenib and sorafenib. As these drugs are in clinical trials and use, respectively, our data might inform decisions concerning the inclusion of patients into clinical trials involving naporafenib and other emerging third-generation RAFis, or to recommend off-label and compassionate use of sorafenib. As suggested previously (*35*, *36*) and as extended by fig. S8D, other type II inhibitors in (pre)clinical development might represent attractive alternatives as well.

The varying efficacy of the type I^1/2^ inhibitors vemurafenib, dabrafenib, and encorafenib against BRAF^Δβ3-αC^ mutants represents an interesting aspect from both a clinical and a basic kinase biochemistry perspective. We confirm previous observations that vemurafenib is ineffective against BRAF^Δβ3-αC^ mutants (*35*, *36*), while encorafenib, an inhibitor not yet tested on these oncoproteins, shows limited and probably insufficient activity. In line with case reports demonstrating therapeutic responses of two BRAF^ΔNVTAP^-positive PDACs (*89*, *97*) and an LCH (*98*) to (initial) dabrafenib monotherapy, we [and (*35*)] show that dabrafenib strongly suppresses BRAF^ΔNVTAP^-driven MEK/ERK phosphorylation in OV-90 cells. While our manuscript was under review, another case report presented an at least 18-month-lasting partial response of a melanoma containing a similar BRAF^ΔNVTAP^ mutant to dabrafenib/trametinib combination therapy (*99*). As there is now more than a decade of clinical experience with dabrafenib, the efficacy of this compound against BRAF^Δβ3-αC^ oncoproteins is of particular interest. Chen *et al.* (*35*), however, observed that dabrafenib only had minimal effects on MEK/ERK phosphorylation in NCI-H2405 (BRAF^ΔLNVTAP>Y^) and BxPC3 (BRAF^ΔVTAPTP>A^) cells. We reproduced these findings (Fig. 6G) and decided to use our heterologous HEK293T model, which does not harbor the caveat of distinct genetic and histological backgrounds, as it is the case for cell lines, to clarify whether the difference in dabrafenib sensitivity is linked to the individual deletion type. Only BRAF^ΔNVTAP^ and BRAF^ΔVTAPTP>A^ displayed dabrafenib sensitivity (Fig. 5F). As BRAF^ΔLNVTAP>F^ showed a significantly higher propensity for homodimerization than BRAF^ΔNVTAP^ (Fig. 3, A and B), it is tempting to attribute the observed dabrafenib resistance of the former to increased dimerization and negative allostery (*19*). In that regard, Foster *et al.* (*36*) demonstrated that, in contrast to dabrafenib, vemurafenib induces and requires a greater αC helix shift during inhibitor accommodation that cannot be provided by BRAF^ΔNVTAP^ because of the sterical constraints imposed by the in-frame deletion. Therefore, the dabrafenib-resistant BRAF^ΔLNVTAP>F^, BRAF^ΔLNVTAP>Y^, BRAF^ΔLNVT>F^, and BRAF^delinsFS^ mutants (Fig. 5, B, F, D, and H), which have not yet been investigated for their impact on αC helix flexibility, might differ from BRAF^ΔNVTAP^ in such a way that their deletions, which are accompanied by insertions of a bulky aromatic residue, preclude dabrafenib binding by negative allostery. The aforementioned study by Zhang *et al.* (*15*), who describe the critical role of hydrophobic interactions occurring during RAF activation, proposes that dimerization tightens the adenosine triphosphate–binding pocket and thereby interferes with inhibitor accommodation by steric clashes in which minute differences between the various RAFi might have large effects.

Another recent study, however, showed that the binding affinities of the dimer-favoring type II inhibitor naporafenib and dabrafenib to chemically enforced BRAF^V600E^ dimers are comparable, demonstrating that increased dimerization is insufficient to confer resistance to type I^1/2^ inhibitors (*25*). Moreover, the inability of the R509H substitution, alone or combined with the AAE mutation, to improve dabrafenib sensitivity in BRAF^ΔLNVTAP>F^ suggests that other mechanisms decide drug accommodation (fig. S11). For example, the mechanism for dabrafenib resistance could be explained by the hydrophobic network that underlies the formation of the R-spine (*15*, *41*). In that respect, we noted that BRAF^ΔLNVTAP>F^, BRAF^ΔLNVTAP>Y^, BRAF^ΔLNVT>F^, and BRAF^delinsFS^ substitute L485 for a bulky aromatic residue that could restrict dabrafenib binding, while the in terms of deletion length similar BRAF^ΔVTAPTP>A^ mutant remained dabrafenib sensitive. This concept is supported by a study proposing that the introduced phenylalanine of the BRAF^L485F^ point mutant forms a hydrophobic network with F498, which in turn stabilizes the R-spine and precludes type I^1/2^ inhibitor binding (*66*). Consequently, loss of F498 should break dabrafenib resistance. The F498A substitution reduced the dabrafenib resistance of BRAF^ΔLNVTAP>F^ by 50% (fig. S12, D and E). This finding supports this model but also suggests that other mechanisms contribute to the dabrafenib resistance of BRAF^ΔLNVTAP>F^, BRAF^ΔLNVTAP>Y^, BRAF^ΔLNVT>F^, and BRAF^delinsFS^. Therefore, the insertion of de novo hydrophobic amino acids at the in-frame deletion junction might generate a distinct mutation-specific hydrophobic network or modify the already recognized ones (*15*) and thereby increase dimerization propensity, activity, and type I^1/2^ RAFi resistance. This represents an interesting area for future studies. Moreover, as the spectrum of tumor-associated BRAF^Δβ3-αC^ will probably expand in the near future, we will learn whether there is a selective pressure for maintaining or even replacing L485 with bulky hydrophobic residues to increase oncogenic potential and type I^1/2^ inhibitor resistance. By revisiting the deletion walking experiment by Foster and colleagues (*36*), we realized that their data also support the critical role of L485 as in-frame deletions omitting L485 hardly increased the MEK phosphorylation potential of BRAF. The analysis of BRAF^ΔLNVTAP>F^ crystal structures, which are not available to date, could potentially reveal the mechanisms conferring resistance against type I^1/2^ RAFis. Close inspection of the orientation and potential intramolecular interactions of F485 of BRAF^ΔLNVTAP>F^ compared to those of L485 of BRAF^ΔNVTAP^ will be key. Although the precise mechanism(s) of dabrafenib resistance need to be addressed in separate studies, our data already demonstrate that BRAF^Δβ3-αC^ oncoproteins significantly differ in their sensitivity toward this compound. Thus, extra caution must be applied when experiences from individual case reports with marked responses for one BRAF^Δβ3-αC^ mutant to dabrafenib are used as evidence to tailor a therapy for an oncoprotein with a seemingly highly similar but distinct alteration.

We also obtained previously unknown insights into the requirements of BRAF^Δβ3-αC^ mutants for oncogenic signaling. A major open question was whether BRAF^Δβ3-αC^ mutants signal as monomers or (constitutive) dimers (*24*, *35*–*37*). In hindsight, this controversy was partly caused by the fact that the various laboratories investigated distinct BRAF^Δβ3-αC^ mutants and used the R509H mutation as a tool to measure dimer dependency. In that regard, the R509H mutation reliably blocks allosteric transactivation, a process from which BRAF^V600E^ and potentially other high-activity mutants are largely exempted (*9*). However, while the R509H mutation strongly reduces homodimer affinity, it does not completely abrogate dimerization, and only the additional introduction of the AAE mutation really renders almost all BRAF molecules monomeric, at least according to coimmunoprecipitation experiments. Thus, the suitability of the R509H mutation to discriminate between dimeric and monomeric BRAF needs to be taken with caution, in particular, within the context of mutants with high dimerization propensity (*24*). Therefore, we revisited the dimerization potential of BRAF^Δβ3-αC^ mutants and demonstrate that they form very stable homodimers with a significantly higher efficiency than BRAF^V600E^. On the basis of these data and work by others on BRAF^ΔNVTAP^ and other in-frame deletion mutants (*24*, *35*), we conclude that BRAF^Δβ3-αC^ mutants signal as dimers because they lose their high MEK phosphorylation and dimerization potential upon the simultaneous introduction of the R509H and AAE mutations. We interpret the relative resistance of the BRAF^Δβ3-αC^ mutants toward the R509H mutation by the aforementioned structural peculiarities of these oncoproteins that, due to their special αC helix conformation, lock them in an active state. Thereby, they become independent of allosteric transactivation that is critical for other BRAF gain-of-function mutants (*3*). Like BRAF^V600E^, but unlike other BRAF oncoproteins (*18*, *40*), Δβ3-αC mutants signal independent of D^594^FGLATV^600^KS-motif phosphorylation, which further supports the notion that the in-frame deletion constitutively induces the active αC-helix-IN/DFG-IN/R506-IN conformation. In all these respects, BRAF^Δβ3-αC^ mutants resemble the canonical class I mutant BRAF^V600E^, although their varying sensitivity toward type I^1/2^ inhibitors argues against this categorization. Thus, these oncoproteins might constitute a class of their own. Another notable parallel to BRAF^V600E^ is the ability of BRAF^Δβ3-αC^ mutants to form large multiprotein complexes. We demonstrated previously that these large multiprotein complexes reflect BRAF activity as endogenous BRAF^V600E^ shifts to the small complex upon vemurafenib treatment (*23*). As we had demonstrated that the large BRAF^V600E^-containing complex is enriched with the HSP90/CDC37 complex and because BRAF^V600E^ shows a specific vulnerability toward HSP90 inhibition (*47*, *48*, *86*), we investigated the association of BRAF^Δβ3-αC^ mutants with HSP90. BRAF^delinsFS^ recruits HSP90 to a similar extent as BRAF^V600E^, while recruitment of HSP90 to BRAF^ΔNVTAP^ was even more pronounced. Unexpectedly, BRAF^ΔLNVTAP>F^ recruits less of this chaperone. In line with this result, BRAF^ΔNVTAP^ and BRAF^V600E^ become unstable in the presence of the clinically trialed HSP90 inhibitor XL888 and present with a significantly shorter half-life. Commensurate with its lower HSP90 recruitment propensity (like BRAF^WT^), BRAF^ΔLNVTAP>F^ displays higher overall stability in our tet-washout experiments and in the presence of XL888. It is tempting to speculate that the very high homodimer formation shown by BRAF^ΔLNVTAP>F^ stabilizes this oncoprotein and alleviates the need for being chaperoned by HSP90/CDC37.

Two recent studies might provide additional explanations for the contrasting HSP90 binding behavior of BRAF^Δβ3-αC^ mutants (*100*, *101*). Using cryo–electron microscopy (cryo-EM), it was shown that the HSP90/CDC37 complex binds to the C-lobe of the kinase domains of BRAF and RAF1, while the latter, which shows higher affinity to the chaperone complex, also binds to the N-lobe and in the vicinity of the Δβ3-αC segment (*101*). This interaction requires the unfolding of the N-lobe, which remains folded in the context of BRAF^WT^ but becomes unstructured and more RAF1-like in the context of the specific conformation of BRAF^V600E^ imposed by the aforementioned salt bridge linking N- and C-lobes. This explains why BRAF^V600E^ tightly interacts with this chaperone (*23*) and is exquisitely sensitive to HSP90 inhibition (*47*, *48*). Thus, deletion of Δβ3-αC segment might restructure the BRAF N-lobe in such a way that it becomes an interaction point for the HSP90/CDC37 complex. This concept would explain why several but not all BRAF^Δβ3-αC^ mutants tightly copurify with HSP90/CDC37 and how specific details of the in-frame deletion modulate this interaction. The BRAF^∆LNVTAP>F^ mutant and, potentially due to structural similarities, BRAF^ΔLNVTAP>Y^ represent a notable exception for the increased HSP90 binding observed for other BRAF^Δβ3-αC^ oncoproteins (Fig. 3, A, B, and D). On the basis of very recent insights from cryo-EM and deuterium exchange/MS experiments showing that the HSP90/CDC37 complex recognizes RAF molecules with N and C loop unfolded (*101*–*103*) and the notion that R-spine formation–induced conformational changes are a prerequisite for efficient N- and C-loop compaction and hence DIF-mediated dimerization (*15*, *17*, *93*), we posit that it is the high dimerization propensity of BRAF^∆LNVTAP>F^ that precludes its interaction with the chaperone complex. This hypothesis is supported by our experiment in which the R509H and AAE mutations increased HSP90 binding of BRAF^∆LNVTAP>F^ (Fig. 3D). Although this represents an area for future studies, our present data nevertheless suggest that inhibitors targeting specific HSP90/CDC37 complexes could be useful to treat tumors driven by other BRAF^Δβ3-αC^ mutants, e.g., to enhance the efficacy of RAFi. Therefore, our study highlights the so-far unrecognized functional diversity of BRAF^Δβ3-αC^ oncoproteins and recommends that precision and attention to molecular details must be applied when potentially effective but highly discriminating type I^1/2^ inhibitors like dabrafenib are considered. Encouraging responses with dabrafenib were observed in individual PDAC cases of BRAF^ΔNVTAP^-driven tumors (*89*, *97*) and in the OV-90 cell line containing the same in-frame deletion (Fig. 6, A, D, G, M, and K). However, two BRAF^ΔNVTAP^-containing PDAC PDOs hardly responded to clinically meaningful dabrafenib concentrations, possibly because of private co-mutations as discussed above. Likewise, the BxPC3 cell line containing the dabrafenib-sensitive BRAF^ΔVTAPTP>A^ mutant appeared less BRAF addicted, probably because of its high pAKT levels. Thus, our data suggest an algorithm for targeted therapy recommendations (fig. S16) based on structural differences predicting intrinsic dabrafenib sensitivity/resistance and based on private comutations and/or expression levels of signaling elements modulating dabrafenib responsiveness. Last, our data provide impetus for the clinical development of safe and effective pan-RAFis that block the activity of all BRAF^Δβ3-αC^ oncoproteins, irrespective of their intrinsic dabrafenib sensitivity or the private signaling network of the tumor cells.

## MATERIALS AND METHODS

### Patient

The patient had given written informed consent for molecular analysis (whole-genome/exome and RNA sequencing, DNA methylation profiling) within NCT/DKTK MASTER, a prospective observational and registry study approved by the Ethics Committee of Heidelberg University (protocol number S-206/ 2011) in which patients with advanced rare cancers and patients diagnosed with advanced common cancers at an unusually young age undergo a standardized precision oncology workflow, including clinical decision-making in a multi-institutional MTB (*38*, *104*).

### Cell lines and genomic DNA analysis

Plat-E cells were provided by T. Kitamura (University of Tokyo). HEK293T cells were provided in-house by A. Hecht. Plat-E, HEK293T cells, and pBABE-puro-CreER^T2^-transduced *Braf*^floxE12/floxE12^ MEF, which were generated in-house and are immortalized by simian virus 40 large T antigen expression (*9*), were cultivated in Dulbecco’s modified Eagle’s medium (DMEM) (4.5 g/liter glucose) supplemented with 10% fetal calf serum (FCS), 2 mM l-glutamine, 10 mM Hepes, penicillin (200 U/ml), and streptomycin (200 μg/ml). OV-90 and NCI-H2405 cells were purchased from American Type Culture Collection. BxPC3 cells were a gift of S. Diederichs (University Medical Center Freiburg). These three cell lines were maintained in RPMI 1640 supplemented with 10% FCS, 2 mM l-glutamine, 10 mM Hepes, penicillin (200 U/ml), and streptomycin (200 μg/ml) and were authenticated by genomic DNA (gDNA)–based PCR analysis confirming the presence of the *BRAF* mutations previously reported for these cell lines (*35*, *36*). To this end, gDNA was extracted using standard protocols and used as template for a PCR using Phusion polymerase (NEB) and oligonucleotides matching to introns 11 (5′- GGAGGATCCCCATGGAACAAACAAGGTTG-3′) and 12 (5′- GGAGAATTCCCACCTCTAAATGTATTCTG-3′) of *BRAF*. PCR amplicons were subcloned into pSC-A (Stratagene) for further analysis (fig. S12, A to C). Absence of mycoplasma was confirmed by PCR (Eurofins Genomics, Ebersberg, Germany).

### PDO establishment, culture, and drug tests

The COMP-196 PDO was identified within a cohort of PDAC PDOs established at the Princess Margaret Cancer Centre Living Biobank (https://pmlivingbiobank.uhnresearch.ca/) from patients enrolled in the COMPASS trial (*105*, *106*) and using procedures previously described in detail for xenograft-derived organoids (*107*). In brief, percutaneous core biopsy tissue from a liver metastasis was minced and dissociated in 1 ml of advanced DMEM (adDMEM)/F12 with 100 μl of Liberase TH (Sigma-Aldrich) and 10 μM Y-27632 at 37°C for 15 min. Cell pellets were washed with adDMEM/F12, counted, and plated in Matrigel with modified human organoid medium [adDMEM/F12, 20% (v/v) Wnt-3a conditioned media, 30% (v/v) R-Spondin1 conditioned media, 1× B27, 2 mM GlutaMAX, 10 mM Hepes, antibiotic-antimycotic (100 U/ml), 1 mM nicotinamide, 1.25 mM *N*-acetyl cysteine, 10 nM gastrin I, hNoggin (100 ng/ml), FGF10 (100 ng/ml), EGF (50 ng/ml), 0.5 μM A 83-01, 10 μM Y-27632, and 2.5 μM CHIR-99021]. For drug tests, domes were dissolved and passaged in TrypLE (Gibco) for 30 to 60 min and counted in trypan blue. Cells were seeded in 10 μl of Matrigel in a 384-well plate at 1000 cells per well overlain with 40 μl of human organoid media (day 1). After 24-hour recovery, drugs were added using a Tecan D300e dispenser (day 2). Viability was measured using Cell Titre Glo 3D after 1 week (day 8).

The B188 PDO was identified within a cohort of pancreatic carcinoma PDOs established at the University Medical Centre Freiburg, Germany. Informed consent was obtained from patients for the establishment and use of three-dimensional (3D) organoid cultures from human pancreatic cancer tissue samples. Sampling was approved by the local Ethics Committee of the University of Freiburg Medical Center (126/17; 28 March 2017). Surgery was performed at the Department of General and Visceral Surgery of the University Hospital Freiburg for proven or suspected pancreatic cancer. Organoid derivation and cultivation protocols were adapted from previous publications (*108*, *109*). In brief, tissue samples were minced into small fragments and digested in 3 ml of complete collagenase digestion buffer [1× human complete feeding medium (COM), Collagenase Crude Type XI (5 mg/ ml; Sigma-Aldrich), 10.5 μM Y-27632, and deoxyribonuclease (DNAse) (10 μg/ml)]. COM consists of 1× HuWa medium [1× adDMEM/ F-12, 10 mM Hepes (pH 7.2 to 7.5), 1× GlutaMAX supplement (all three from Gibco), and Primocin (100 μg/ml; InVivoGen)], 1× Wnt3a-conditioned medium or Afamin/Wnt3a-conditioned medium, 1× R-Spondin1–conditioned medium, 1× B27 supplement (Gibco), 10 mM nicotinamide, 1.25 mM *N*-acetylcysteine (both from Sigma-Aldrich), Plasmocin (2.5 μg/ml; InVivoGen), hEGF (50 ng/ml), hFGF10 (100 ng/ml), 10 nM hGastrin I (all three from PeproTech), 500 nM A 83-01 (TOCRIS), and 10.5 μM Y-27632 (Sigma-Aldrich).

In total, two incubation steps in a rotating incubator were performed at 37°C for 15 min. After each incubation, the digested tissue was manually triturated 10 to 20 times, and the supernatant of both fractions was centrifuged at 4°C, 200*g* for 5 min. The cells were resuspended in 2 ml of ACK lysing buffer (Gibco), incubated for 2 min, and spun again. Subsequently, the cells were washed once with HuWa medium containing 0.1% bovine serum albumin (BSA; Sigma-Aldrich). The cell pellet was resuspended in an adequate amount of Matrigel (8 mg/ml; Corning). New domes with 25 μl of Matrigel each were made and incubated at 37°C, 5% CO_2_ for 15 to 20 min. Thereafter, 500 μl of Complete Organoid Medium with Wnt (COM-Wnt) or Wnt-Afamin (COM-W/A) supplemented with 10.5 μM Y-27632 was added. PDOs were grown 6 to 12 days at 37°C, 5% CO_2_ and checked every third day. NGS sequencing leading to the identification of the *BRAF*^ΔNVTAP^ mutation was performed at University Spital Zürich, Molecular Pathology department (sequencing type, FoundationOne CDx).

To passage PDOs, two Matrigel domes were pooled and dissolved in 500 μl of ice-cold cell recovery solution (CRS). Subsequently, the suspension was incubated for 30 min on ice, inverting the tube every 10 min. The cells were pelleted, and the CRS was discarded. The cell pellet was resuspended in 2 ml of TrypLE Master mix [1.5 ml of 1× TrypLE Express Enzyme (Gibco), 0.5 ml of HuWawith, 0.1% BSA, 10.5 μM Y-27632, and DNase (10 μg/ml)]. The cells were incubated in a rotating incubator at 37°C and 180 rpm for 15 min. Cells were pelleted again, and the supernatant was aspirated. The cell pellet was resuspended 20 times in ice-cold HuWa medium with 0.1% BSA to mechanically dissociate the PDOs. Following a last centrifugation step, cells were resuspended in Matrigel, and new domes (25 μl of Matrigel each) were spotted into tissue culture wells incubated at 37°C, 5% CO_2_ for 15 to 20 min before being overlain with 500 μl of COM-Wnt or COM-W/A supplemented with 10.5 μM Y-27632. Occasionally, an aliquot of cells was used to isolate gDNA as described above to confirm the presence of driver mutations.

For drug tests, PDO-containing domes were dissolved as described above, and isolated cells were counted with a Bio-Rad TC20 Automated Cell Counter. Desired number of cells was seeded in a 96-well plate in 5 μl of Matrigel domes with 1000 cells per dome. After incubation at 37°C, 5% CO_2_ for 15 min, 100 μl of COM-W/A medium was added per well. In addition, all empty wells were filled with 120 μl of phosphate-buffered saline (PBS) to decrease medium evaporation. Following cultivation (5% CO_2_, 37°C) for 7 days, the medium was carefully aspirated, and the drugs diluted in either COM-W/A or HuWa were added and incubated for 3 days. Subsequently, 100 μl of Cell Titer Glo 3D (Promega) was added to the wells and resuspended 10 times. After incubation for 30 min in the dark at room temperature, the luminescence signal was measured with a Tecan infinite M200 plate reader (integration time, 100 ms).

### Generation of pCLXEBR-pTF1-HA-BRAF-IRES-GFP (pCLXEBR) MEFs

To generate *Braf*^floxE12/floxE12^ MEFs expressing HA-BRAF proteins upon Tet/Dox induction, recombination of *Braf*^floxE12/floxE12^ MEFs was induced by treatment with 4-hydroxytamoxifen (1 μM). Efficient recombination was confirmed by genomic PCR (*110*) and Western blot analyses (fig. S15). *Braf^−/−^* MEFs were infected with ecotropic lentiviral particles using the packaging plasmids psPAX2 and pCMV_Eco provided by I. Frew (*111*). Successfully infected cells were selected with blasticidine S (5 μg/ml).

### Plasmids

The generation of the bicistronic retroviral vectors pMIG and pMIBerry encoding N-terminally HA-tagged or C-terminally Myc-tagged human BRAF, respectively, as well as the point mutants V600E and F595L was described previously (*9*, *44*). Δβ3-αC mutations, the F498A, and the dimerization-impairing mutations R509H and 621APE-AAE were introduced via site-directed mutagenesis using the oligonucleotides specified in table S1.

To generate tet-inducible pCLXEBR-pTF1-HA-BRAF-IRES-GFP constructs, the HA-BRAF-IRES-GFP insert was excised from corresponding pMIG constructs using *BsrG*I. The tet-inducible pCLXEBR-pTF1-kRasV12 vector, which we obtained from Addgene (plasmid no. 114318; deposited and provided by P. Salmon), was digested with *BsrG*I, thereby removing the kRasV12 encoding insert, followed by ligation of HA-BRAF-IRES-GFP insert and pCLXEBR-pTF1 vector backbone.

### Antibodies and reagents

Antibodies used in this study were anti–B-RAF (D9T6S), anti-GFP, anti-HSP90, anti-p44/42 MAPK (ERK1/2), anti–phospho-p44/22 (ERK1/2) (Thr^202^/Tyr^204^), anti-MEK1/2, anti–phospho-MEK1/2 (Ser^217/212^), anti-AKT, anti–phospho-AKT (S473), anti-EGFR (D38B1), anti–phospho-EGFR (Tyr^1068^) (D7A5) (all from Cell Signaling Technology), anti–RAF-B (F-7), anti–α-tubulin (Santa Cruz Biotechnology), anti–glyceraldehyde-3-phosphate dehydrogenase (Abcam), and anti-HA (3F10) (Roche Diagnostics). Belvarafenib (HM95573), dabrafenib, encorafenib, GDC-0879, lifirafenib (BGB-283), LY3009120, MLN2480, naporafenib (LXH254), sorafenib, TAK-632, trametinib, vemurafenib, and XL888 were purchased from SelleckChem. All inhibitors were dissolved in dimethyl sulfoxide (DMSO).

### Western blotting and BN-PAGE

Western blotting was carried out as previously described (*9*). Briefly, cells were lysed in normal lysis buffer [NLB; 50 mM tris/HCl (pH 7.5), 1% Triton X-100, 137 mM sodium chloride, 1% glycerine, 1 mM sodium orthovanadate, 0.5 mM EDTA, leupeptin (0.01 mg/ml), aprotinin (0.1 mg/ml), and 1 mM 4-(2-Aminoethyl)benzenesulfonyl fluoride hydrochloride (AEBSF)], separated on SDS gels containing 10% polyacrylamide and transferred to polyvinylidene difluoride membranes. Blotted proteins were visualized using horseradish peroxidase–conjugated secondary antibodies (Thermo Fisher Scientific), SuperSignal West Femto Maximum Sensitivity Substrate (Thermo Fisher Scientific), and a Fusion Solo imaging system (Vilber). Signals were quantified using ImageJ.

For BN-PAGE, transiently transfected HEK293T cells were harvested 2 days after transfection. Before harvest, cell culture dishes were washed twice with ice-cold PBS. Ice-cold BN-PAGE lysis buffer [20 mM bis-tris, 20 mM NaCl, 2 mM EDTA, 10% glycerol, 0.1% Triton X-100, protease inhibitor cocktail (Roche, no. 11836145001), and PhoSTOP (Roche no. 04906837001) (pH 7)] was added directly to the plates, which were left on a rocking platform at 4°C for 30 min for cell lysis. Cells were scraped and lysates transferred into fresh reaction tubes and centrifuged at 4°C at 15,700*g* for 10 min, and supernatant was transferred into fresh reaction tubes for gel loading. BN-PAGE was performed according to instructions of the manufacturer [NativePAGE Novex 3 to 12% bis-tris protein gels, 1.0 mm, 10 well (Invitrogen, BN1001)] at 4°C. In brief, 50 μl of lysate were mixed with 150 μl of a glycerol-BN-PAGE lysis buffer solution (1:2). Wells were visualized for sample loading by flushing them two to three times with dark-blue cathode buffer (Invitrogen, BN2002), and the front part of the chamber was filled half with dark-blue cathode buffer. Twenty microliters of each sample was loaded into pockets. The front part of the chamber was completely filled with dark-blue cathode buffer, and afterward, the back part of the chamber was filled with transparent anode buffer (Invitrogen, BN2001). Proteins were separated at 100 V and 4°C for 60 min. Thereafter, the dark-blue cathode buffer was changed to light-blue cathode buffer (Invitrogen, BN2002), and the electrophoresis was continued at 200 V for additional 1 hour and 15 min, followed by Western blots. Signals were quantified using ImageJ.

### Immunoprecipitations and MS

For immunoprecipitations, HEK293T cells transiently coexpressing HA- and Myc-BRAF proteins that were grown to subconfluency on a 10-cm dish were lysed in 1 ml of NLB 48 hours after transfection. Next, 0.5 μg of anti-HA antibody was added to 900 μl of cleared total cell lysates, followed by 1 hour of incubation on ice. Fifty microliters of Protein G-Sepharose slurry was added, followed by incubation at 4°C overnight, rotating. Beads were washed eight times with 1 ml of NLB. Following resuspension in 100 μl of NLB, addition of Laemmli buffer and boiling for 5 min, samples were analyzed via Western blotting.

For MS analysis of HSP90i-treated OV-90 cells (*BRAF*^*WT/*Δ*NVTAP)*^), 40 15-cm dishes of subconfluent OV-90 cells were cultivated in the presence of XL888 (1 μM) or control (DMSO) for 24 hours before lysis in NLB (800 μl per dish). Cleared lysates were combined and incubated in the presence of an anti-BRAF antibody cocktail [150 μl of anti–RAF-B (F-7) and 100 μl of anti–B-RAF (D9T6S)] 1 hour on ice, followed by addition of 200 μl of Protein G-Sepharose slurry and incubation at 4°C overnight, rotating. Beads were washed five times and subjected to MS analysis.

For MS, samples were taken up in Laemmli sample buffer, reduced with 1 mM dithiothreitol for 10 min at 75°C, and alkylated using 5.5 mM iodoacetamide for 10 min at room temperature. The same amount of each sample was loaded on 4 to 12% gradients gels. The gel area corresponding to 80 to 100 kDa was excised and cut into small pieces, and proteins therein were in-gel digested with trypsin (Promega). Tryptic peptides were purified by STAGE tips before liquid chromatography tandem MS (LC-MS/MS) measurements. The LC-MS/MS measurements were performed on an Exploris 480 mass spectrometer coupled to an EasyLC 1200 nanoflow–high-performance liquid chromatography (HPLC). Peptides were separated on fused silica HPLC-column tip [inside diameter, 75 μm, New Objective, self-packed with reprosil-Pur 120 C18-AQ, 1.9 μm (Dr. Maisch) to a length of 20 cm] using a gradient of A (0.1% formic acid in water) and B (0.1% formic acid in 80% acetonitrile in water). A mass spectrometer was operated in the data-dependent mode; after each MS scan (mass range *m*/*z* = 370 to 1750; resolution, 120,000), a maximum of 20 MS/MS scans were performed using a normalized collision energy of 28%, a target value of 50%, and a resolution of 15,000. MS raw files were processed with MaxQuant software (version 2.0.1.0) using a Uniprot human database containing all BRAF variants and standard settings (*112*).

### Transfection, infection, and focus formation assays

Transient transfection of Plat-E and HEK293T cells was carried out as previously described (*9*). For Western blot analysis, cells were lysed 48 hours after transfection. Viral supernatants of Plat-E cells were harvested and used for infection after 48 hours as well. Infection of MEFs and subsequent foci formation assays were carried out as described previously (*40*, *44*).

### Colony formation assays

Cells were plated on six-well plates (1200 cells per well). Inhibitors were added the following day. Medium, supplemented with inhibitors, was changed every 2 to 3 days. Colonies of OV-90, BxPC3, and H2405 cells were stained with 0.1% crystal violet staining solution after 16, 18, or 21 days, respectively. Stained six-well plates were digitalized by scanning followed by quantification of colonized areas using ImageJ.

### XTT assay

Cells were seeded onto 96-well plates (OV-90 and NCI-H2405 4000, BxPC3 2000 cells per well) and incubated with inhibitor or vehicle control (DMSO) for 96 hours. Inhibitor titrations were performed with a Tecan D300e device. Subsequently, the metabolic activity was measured using the Cell Proliferation Kit II (Roche Diagnostics) according to the manufacturer’s protocol.

### BRAF stability (tet washout) and HSP90 inhibition assays

To determine BRAF stability, pCLXEBR MEFs were grown in the presence of tet (20 μg/ml), which is less stable than its analog doxycycline, for 30 hours to induce expression of HA-BRAF proteins. Following tet washout to stop transcription of the BRAF expression cassette and a waiting time of 26 hours to allow for depletion of residual tet and tet-induced mRNA, cells were grown for the indicated times. BRAF levels were determined by Western blot and normalized to α-tubulin. Protein half-lives were calculated using one-phase decay function. To analyze the effect of HSP90 inhibition on the stability of BRAF proteins, pCLXEBR MEFs were grown in the presence of doxycycline (50 ng/ml). After 72 hours, the HSP90 inhibitor XL888 (1 μM) was added. Cells were subject to Western blot analysis after 0, 5, 8, and 24 hours after HSP90i.

### Cellular thermal shift assay

HEK293T cells transiently expressing HA-BRAF proteins were detached by trypsin, suspended in DPBS (10 × 10^6^ cells/ml), and incubated in the presence of 100 μM dabrafenib or vehicle control (DMSO) for 4 hours, rotating at room temperature. Cells were divided into 100 μl aliquots and heated in a PCR machine at increasing temperatures (42° to 54°C) for 3 min. Subsequently, lysis was performed in NLB for 10 min. Denatured and precipitated BRAF protein was removed by centrifugation at 16,000*g*, 4°C for 15 min. Levels of residual native BRAF protein were analyzed by Western blotting.

### Statistical analysis

The number of individual experiments as well as the applied statistical tests were specified in the respective figure legend. Data are presented as means + SD, if not stated otherwise. Statistical analyses were performed using GraphPad Prism 9 (GraphPad Inc., CA).

## Supplementary Materials

### This PDF file includes:

Figs. S1 to S16 Table S1 References

## References

[1] M. L. Turski, S. J. Vidwans, F. Janku, I. Garrido-Laguna, J. Munoz, R. Schwab, V. Subbiah, J. Rodon, R. Kurzrock, Genomically driven tumors and actionability across histologies: BRAF-mutant cancers as a paradigm. Mol. Cancer Ther. 15, 533–547 (2016). 27009213 10.1158/1535-7163.MCT-15-0643

[2] F. A. Cook, S. J. Cook, Inhibition of RAF dimers: It takes two to tango. Biochem. Soc. Trans. 49, 237–251 (2021). 33367512 10.1042/BST20200485 PMC7924995

[3] T. Brummer, C. McInnes, RAF kinase dimerization: Implications for drug discovery and clinical outcomes. Oncogene 39, 4155–4169 (2020). 32269299 10.1038/s41388-020-1263-y

[4] J. A. Martinez Fiesco, D. E. Durrant, D. K. Morrison, P. Zhang, Structural insights into the BRAF monomer-to-dimer transition mediated by RAS binding. Nat. Commun. 13, 486 (2022). 35078985 10.1038/s41467-022-28084-3 PMC8789793

[5] Y. Kondo, J. Ognjenović, S. Banerjee, D. Karandur, A. Merk, K. Kulhanek, K. Wong, J. P. Roose, S. Subramaniam, J. Kuriyan, Cryo-EM structure of a dimeric B-Raf:14-3-3 complex reveals asymmetry in the active sites of B-Raf kinases. Science 366, 109–115 (2019). 31604311 10.1126/science.aay0543 PMC7007921

[6] E. Park, S. Rawson, K. Li, B. W. Kim, S. B. Ficarro, G. G. D. Pino, H. Sharif, J. A. Marto, H. Jeon, M. J. Eck, Architecture of autoinhibited and active BRAF–MEK1–14-3-3 complexes. Nature 575, 545–550 (2019). 31581174 10.1038/s41586-019-1660-y PMC7014971

[7] R. Röck, J. E. Mayrhofer, O. Torres-Quesada, F. Enzler, A. Raffeiner, P. Raffeiner, A. Feichtner, R. G. Huber, S. Koide, S. S. Taylor, J. Troppmair, E. Stefan, BRAF inhibitors promote intermediate BRAF(V600E) conformations and binary interactions with activated RAS. Sci. Adv. 5, eaav8463 (2019). 31453322 10.1126/sciadv.aav8463 PMC6693913

[8] T. Rajakulendran, M. Sahmi, M. Lefrancois, F. Sicheri, M. Therrien, A dimerization-dependent mechanism drives RAF catalytic activation. Nature 461, 542–545 (2009). 19727074 10.1038/nature08314

[9] M. Röring, R. Herr, G. J. Fiala, K. Heilmann, S. Braun, A. E. Eisenhardt, S. Halbach, D. Capper, A. von Deimling, W. W. Schamel, D. N. Saunders, T. Brummer, Distinct requirement for an intact dimer interface in wild-type, V600E and kinase-dead B-Raf signalling. EMBO J. 31, 2629–2647 (2012). 22510884 10.1038/emboj.2012.100 PMC3365413

[10] H. Lavoie, M. Therrien, Regulation of RAF protein kinases in ERK signalling. Nat. Rev. Mol. Cell Biol. 16, 281–298 (2015). 25907612 10.1038/nrm3979

[11] A. K. Freeman, D. A. Ritt, D. K. Morrison, Effects of Raf dimerization and its inhibition on normal and disease-associated Raf signaling. Mol. Cell 49, 751–758 (2013). 23352452 10.1016/j.molcel.2012.12.018 PMC3582845

[12] J. Hu, E. C. Stites, H. Yu, E. A. Germino, H. S. Meharena, P. J. S. Stork, A. P. Kornev, S. S. Taylor, A. S. Shaw, Allosteric activation of functionally asymmetric RAF kinase dimers. Cell 154, 1036–1046 (2013). 23993095 10.1016/j.cell.2013.07.046 PMC3844432

[13] Y. Kondo, J. W. Paul III, S. Subramaniam, J. Kuriyan, New insights into Raf regulation from structural analyses. Curr. Opin. Struct. Biol. 71, 223–231 (2021). 34454301 10.1016/j.sbi.2021.07.005

[14] H. R. Mott, D. Owen, SHOCing RAF into action. Nat. Struct. Mol. Biol. 29, 958–960 (2022). 36192652 10.1038/s41594-022-00843-2

[15] M. Zhang, R. Maloney, H. Jang, R. Nussinov, The mechanism of Raf activation through dimerization. Chem. Sci. 12, 15609–15619 (2021). 35003591 10.1039/d1sc03444h PMC8654025

[16] B. H. Zhang, K. L. Guan, Activation of B-Raf kinase requires phosphorylation of the conserved residues Thr598 and Ser601. EMBO J. 19, 5429–5439 (2000). 11032810 10.1093/emboj/19.20.5429 PMC314015

[17] N. Thevakumaran, H. Lavoie, D. A. Critton, A. Tebben, A. Marinier, F. Sicheri, M. Therrien, Crystal structure of a BRAF kinase domain monomer explains basis for allosteric regulation. Nat. Struct. Mol. Biol. 22, 37–43 (2015). 25437913 10.1038/nsmb.2924

[18] M. Köhler, M. Röring, B. Schorch, K. Heilmann, N. Stickel, G. J. Fiala, L. C. Schmitt, S. Braun, S. Ehrenfeld, F. M. Uhl, T. Kaltenbacher, F. Weinberg, S. Herzog, R. Zeiser, W. W. Schamel, H. Jumaa, T. Brummer, Activation loop phosphorylation regulates B-Raf in vivo and transformation by B-Raf mutants. EMBO J. 35, 143–161 (2016). 26657898 10.15252/embj.201592097 PMC4718462

[19] B. Agianian, E. Gavathiotis, Current insights of BRAF inhibitors in cancer. J. Med. Chem. 61, 5775–5793 (2018). 29461827 10.1021/acs.jmedchem.7b01306

[20] Z. Karoulia, E. Gavathiotis, P. I. Poulikakos, New perspectives for targeting RAF kinase in human cancer. Nat. Rev. Cancer 17, 676–691 (2017). 28984291 10.1038/nrc.2017.79 PMC6000833

[21] Z. Yao, N. M. Torres, A. Tao, Y. Gao, L. Luo, Q. Li, E. de Stanchina, O. Abdel-Wahab, D. B. Solit, P. I. Poulikakos, N. Rosen, BRAF Mutants Evade ERK-dependent feedback by different mechanisms that determine their sensitivity to pharmacologic inhibition. Cancer Cell 28, 370–383 (2015). 26343582 10.1016/j.ccell.2015.08.001 PMC4894664

[22] O. S. Rukhlenko, F. Khorsand, A. Krstic, J. Rozanc, L. G. Alexopoulos, N. Rauch, K. E. Erickson, W. S. Hlavacek, R. G. Posner, S. Gómez-Coca, E. Rosta, C. Fitzgibbon, D. Matallanas, J. Rauch, W. Kolch, B. N. Kholodenko, Dissecting RAF inhibitor resistance by structure-based modeling reveals ways to overcome oncogenic RAS signaling. Cell Syst. 7, 161–179.e14 (2018). 30007540 10.1016/j.cels.2018.06.002 PMC6149545

[23] B. Diedrich, K. T. G. Rigbolt, M. Röring, R. Herr, S. Kaeser-Pebernard, C. Gretzmeier, R. F. Murphy, T. Brummer, J. Dengjel, Discrete cytosolic macromolecular BRAF complexes exhibit distinct activities and composition. EMBO J. 36, 646–663 (2017). 28093501 10.15252/embj.201694732 PMC5331759

[24] J. Yuan, W. H. Ng, P. Y. P. Lam, Y. Wang, H. Xia, J. Yap, S. P. Guan, A. S. G. Lee, M. Wang, M. Baccarini, J. Hu, The dimer-dependent catalytic activity of RAF family kinases is revealed through characterizing their oncogenic mutants. Oncogene 37, 5719–5734 (2018). 29930381 10.1038/s41388-018-0365-2 PMC6202329

[25] C. Adamopoulos, T. A. Ahmed, M. R. Tucker, P. M. U. Ung, M. Xiao, Z. Karoulia, A. Amabile, X. Wu, S. A. Aaronson, C. Ang, V. W. Rebecca, B. D. Brown, A. Schlessinger, M. Herlyn, Q. Wang, D. E. Shaw, P. I. Poulikakos, Exploiting allosteric properties of RAF and MEK inhibitors to target therapy-resistant tumors driven by oncogenic BRAF signaling. Cancer Discov. 11, 1716–1735 (2021). 33568355 10.1158/2159-8290.CD-20-1351 PMC8295204

[26] M. Dankner, A. A. N. Rose, S. Rajkumar, P. M. Siegel, I. R. Watson, Classifying BRAF alterations in cancer: New rational therapeutic strategies for actionable mutations. Oncogene 37, 3183–3199 (2018). 29540830 10.1038/s41388-018-0171-x

[27] P. T. C. Wan, M. J. Garnett, S. M. Roe, S. Lee, D. Niculescu-Duvaz, V. M. Good, C. M. Jones, C. J. Marshall, C. J. Springer, D. Barford, R. Marais; Cancer Genome Project, Mechanism of activation of the RAF-ERK signaling pathway by oncogenic mutations of B-RAF. Cell 116, 855–867 (2004). 15035987 10.1016/s0092-8674(04)00215-6

[28] S. J. Heidorn, C. Milagre, S. Whittaker, A. Nourry, I. Niculescu-Duvas, N. Dhomen, J. Hussain, J. S. Reis-Filho, C. J. Springer, C. Pritchard, R. Marais, Kinase-dead BRAF and oncogenic RAS cooperate to drive tumor progression through CRAF. Cell 140, 209–221 (2010). 20141835 10.1016/j.cell.2009.12.040 PMC2872605

[29] P. Nieto, C. Ambrogio, L. Esteban-Burgos, G. Gómez-López, M. T. Blasco, Z. Yao, R. Marais, N. Rosen, R. Chiarle, D. G. Pisano, M. Barbacid, D. Santamaría, A Braf kinase-inactive mutant induces lung adenocarcinoma. Nature 548, 239–243 (2017). 28783725 10.1038/nature23297 PMC5648056

[30] M. Dankner, M. Lajoie, D. Moldoveanu, T. T. Nguyen, P. Savage, S. Rajkumar, X. Huang, M. Lvova, A. Protopopov, D. Vuzman, D. Hogg, M. Park, M. C. Guiot, K. Petrecca, C. Mihalcioiu, I. R. Watson, P. M. Siegel, A. A. N. Rose, Dual MAPK inhibition is an effective therapeutic strategy for a subset of class II BRAF mutant melanomas. Clin. Cancer Res. 24, 6483–6494 (2018). 29903896 10.1158/1078-0432.CCR-17-3384

[31] A. J. Aguirre, J. A. Nowak, N. D. Camarda, R. A. Moffitt, A. A. Ghazani, M. Hazar-Rethinam, S. Raghavan, J. Kim, L. K. Brais, D. Ragon, M. W. Welch, E. Reilly, D. McCabe, L. Marini, K. Anderka, K. Helvie, N. Oliver, A. Babic, A. da Silva, B. Nadres, E. E. van Seventer, H. A. Shahzade, J. P. St. Pierre, K. P. Burke, T. Clancy, J. M. Cleary, L. A. Doyle, K. Jajoo, N. J. McCleary, J. A. Meyerhardt, J. E. Murphy, K. Ng, A. K. Patel, K. Perez, M. H. Rosenthal, D. A. Rubinson, M. Ryou, G. I. Shapiro, E. Sicinska, S. G. Silverman, R. J. Nagy, R. B. Lanman, D. Knoerzer, D. J. Welsch, M. B. Yurgelun, C. S. Fuchs, L. A. Garraway, G. Getz, J. L. Hornick, B. E. Johnson, M. H. Kulke, R. J. Mayer, J. W. Miller, P. B. Shyn, D. A. Tuveson, N. Wagle, J. J. Yeh, W. C. Hahn, R. B. Corcoran, S. L. Carter, B. M. Wolpin, Real-time genomic characterization of advanced pancreatic cancer to enable precision medicine. Cancer Discov. 8, 1096–1111 (2018). 29903880 10.1158/2159-8290.CD-18-0275 PMC6192263

[32] P. A. Philip, I. Azar, J. Xiu, M. J. Hall, A. E. Hendifar, E. Lou, J. J. Hwang, J. Gong, R. Feldman, M. Ellis, P. Stafford, D. Spetzler, M. M. Khushman, D. Sohal, A. C. Lockhart, B. A. Weinberg, W. S. el-Deiry, J. Marshall, A. F. Shields, W. M. Korn, Molecular characterization of KRAS wild-type tumors in patients with pancreatic adenocarcinoma. Clin. Cancer Res. 28, 2704–2714 (2022). 35302596 10.1158/1078-0432.CCR-21-3581 PMC9541577

[33] R. Ren, S. G. Krishna, W. Chen, W. L. Frankel, R. Shen, W. Zhao, M. R. Avenarius, J. Garee, S. Caruthers, D. Jones, Activation of the RAS pathway through uncommon BRAF mutations in mucinous pancreatic cysts without KRAS mutation. Mod. Pathol. 34, 438–444 (2021). 32792597 10.1038/s41379-020-00647-z

[34] A. Hendifar, E. M. Blais, B. Wolpin, V. Subbiah, E. Collisson, I. Singh, T. Cannon, K. Shaw, E. F. Petricoin III, S. Klempner, E. Lyons, A. Wang-Gillam, M. J. Pishvaian, E. M. O'Reilly, Retrospective case series analysis of *RAF* family alterations in pancreatic cancer: Real-world outcomes from targeted and standard therapies. JCO Precis. Oncol. 5, PO.20.00494 (2021). 34476331 10.1200/PO.20.00494 PMC8407652

[35] S. H. Chen, Y. Zhang, R. D. van Horn, T. Yin, S. Buchanan, V. Yadav, I. Mochalkin, S. S. Wong, Y. G. Yue, L. Huber, I. Conti, J. R. Henry, J. J. Starling, G. D. Plowman, S. B. Peng, Oncogenic braf deletions that function as homodimers and are sensitive to inhibition by RAF dimer inhibitor LY3009120. Cancer Discov. 6, 300–315 (2016). 26732095 10.1158/2159-8290.CD-15-0896

[36] S. A. Foster, D. M. Whalen, A. Özen, M. J. Wongchenko, J. P. Yin, I. Yen, G. Schaefer, J. D. Mayfield, J. Chmielecki, P. J. Stephens, L. A. Albacker, Y. Yan, K. Song, G. Hatzivassiliou, C. Eigenbrot, C. Yu, A. S. Shaw, G. Manning, N. J. Skelton, S. G. Hymowitz, S. Malek, Activation mechanism of oncogenic deletion mutations in BRAF, EGFR, and HER2. Cancer Cell 29, 477–493 (2016). 26996308 10.1016/j.ccell.2016.02.010

[37] D. M. Freed, J. H. Park, R. Radhakrishnan, M. A. Lemmon, Deletion mutations keep kinase inhibitors in the loop. Cancer Cell 29, 423–425 (2016). 27070691 10.1016/j.ccell.2016.03.017 PMC5028821

[38] P. Horak, C. Heining, S. Kreutzfeldt, B. Hutter, A. Mock, J. Hüllein, M. Fröhlich, S. Uhrig, A. Jahn, A. Rump, L. Gieldon, L. Möhrmann, D. Hanf, V. Teleanu, C. E. Heilig, D. B. Lipka, M. Allgäuer, L. Ruhnke, A. Laßmann, V. Endris, O. Neumann, R. Penzel, K. Beck, D. Richter, U. Winter, S. Wolf, K. Pfütze, C. Geörg, B. Meißburger, I. Buchhalter, M. Augustin, W. E. Aulitzky, P. Hohenberger, M. Kroiss, P. Schirmacher, R. F. Schlenk, U. Keilholz, F. Klauschen, G. Folprecht, S. Bauer, J. T. Siveke, C. H. Brandts, T. Kindler, M. Boerries, A. L. Illert, N. von Bubnoff, P. J. Jost, K. Spiekermann, M. Bitzer, K. Schulze-Osthoff, C. von Kalle, B. Klink, B. Brors, A. Stenzinger, E. Schröck, D. Hübschmann, W. Weichert, H. Glimm, S. Fröhling, Comprehensive genomic and transcriptomic analysis for guiding therapeutic decisions in patients with rare cancers. Cancer Discov. 11, 2780–2795 (2021). 34112699 10.1158/2159-8290.CD-21-0126

[39] D. Pratt, S. Camelo-Piragua, K. McFadden, D. Leung, R. Mody, A. Chinnaiyan, C. Koschmann, S. Venneti, BRAF activating mutations involving the β3-αC loop in V600E-negative anaplastic pleomorphic xanthoastrocytoma. Acta Neuropathol. Commun. 6, 24 (2018). 29544532 10.1186/s40478-018-0525-1 PMC5855983

[40] F. Weinberg, R. Griffin, M. Fröhlich, C. Heining, S. Braun, C. Spohr, M. Iconomou, V. Hollek, M. Röring, P. Horak, S. Kreutzfeldt, G. Warsow, B. Hutter, S. Uhrig, O. Neumann, D. Reuss, D. H. Heiland, C. von Kalle, W. Weichert, A. Stenzinger, B. Brors, H. Glimm, S. Fröhling, T. Brummer, Identification and characterization of a BRAF fusion oncoprotein with retained autoinhibitory domains. Oncogene 39, 814–832 (2020). 31558800 10.1038/s41388-019-1021-1

[41] J. Hu, L. G. Ahuja, H. S. Meharena, N. Kannan, A. P. Kornev, S. S. Taylor, A. S. Shaw, Kinase regulation by hydrophobic spine assembly in cancer. Mol. Cell. Biol. 35, 264–276 (2015). 25348715 10.1128/MCB.00943-14 PMC4295384

[42] P. I. Poulikakos, Y. Persaud, M. Janakiraman, X. Kong, C. Ng, G. Moriceau, H. Shi, M. Atefi, B. Titz, M. T. Gabay, M. Salton, K. B. Dahlman, M. Tadi, J. A. Wargo, K. T. Flaherty, M. C. Kelley, T. Misteli, P. B. Chapman, J. A. Sosman, T. G. Graeber, A. Ribas, R. S. Lo, N. Rosen, D. B. Solit, RAF inhibitor resistance is mediated by dimerization of aberrantly spliced BRAF(V600E). Nature 480, 387–390 (2011). 22113612 10.1038/nature10662 PMC3266695

[43] T. Ikenoue, Y. Hikiba, F. Kanai, Y. Tanaka, J. Imamura, T. Imamura, M. Ohta, H. Ijichi, K. Tateishi, T. Kawakami, J. Aragaki, M. Matsumura, T. Kawabe, M. Omata, Functional analysis of mutations within the kinase activation segment of *B-Raf* in human colorectal tumors. Cancer Res. 63, 8132–8137 (2003). 14678966

[44] M. Kordes, M. Röring, C. Heining, S. Braun, B. Hutter, D. Richter, C. Geörg, C. Scholl, S. Gröschel, W. Roth, A. Rosenwald, E. Geissinger, C. von Kalle, D. Jäger, B. Brors, W. Weichert, C. Grüllich, H. Glimm, T. Brummer, S. Fröhling, Cooperation of BRAF(F595L) and mutant HRAS in histiocytic sarcoma provides new insights into oncogenic BRAF signaling. Leukemia 30, 937–946 (2016). 26582644 10.1038/leu.2015.319

[45] A. E. Eisenhardt, A. Sprenger, M. Röring, R. Herr, F. Weinberg, M. Köhler, S. Braun, J. Orth, B. Diedrich, U. Lanner, N. Tscherwinski, S. Schuster, N. Dumaz, E. Schmidt, R. Baumeister, A. Schlosser, J. Dengjel, T. Brummer, Phospho-proteomic analyses of B-Raf protein complexes reveal new regulatory principles. Oncotarget 7, 26628–26652 (2016). 27034005 10.18632/oncotarget.8427 PMC5042004

[46] K. Miyamoto, M. Sawa, Development of highly sensitive biosensors of RAF dimerization in cells. Sci. Rep. 9, 636 (2019). 30679688 10.1038/s41598-018-37213-2 PMC6345758

[47] S. da Rocha Dias, F. Friedlos, Y. Light, C. Springer, P. Workman, R. Marais, Activated B-RAF is an Hsp90 client protein that is targeted by the anticancer drug 17-allylamino-17-demethoxygeldanamycin. Cancer Res. 65, 10686–10691 (2005). 16322212 10.1158/0008-5472.CAN-05-2632

[48] O. M. Grbovic, A. D. Basso, A. Sawai, Q. Ye, P. Friedlander, D. Solit, N. Rosen, V600E B-Raf requires the Hsp90 chaperone for stability and is degraded in response to Hsp90 inhibitors. Proc. Natl. Acad. Sci. U.S.A. 103, 57–62 (2006). 16371460 10.1073/pnas.0609973103 PMC1325013

[49] Z. Eroglu, Y. A. Chen, G. T. Gibney, J. S. Weber, R. R. Kudchadkar, N. I. Khushalani, J. Markowitz, A. S. Brohl, L. F. Tetteh, H. Ramadan, G. Arnone, J. Li, X. Zhao, R. Sharma, L. N. F. Darville, B. Fang, I. Smalley, J. L. Messina, J. M. Koomen, V. K. Sondak, K. S. M. Smalley, Combined BRAF and HSP90 inhibition in patients with unresectable BRAF (V600E)-mutant melanoma. Clin. Cancer Res. 24, 5516–5524 (2018). 29674508 10.1158/1078-0432.CCR-18-0565 PMC6195480

[50] M. A. Hernandez, B. Patel, F. Hey, S. Giblett, H. Davis, C. Pritchard, Regulation of BRAF protein stability by a negative feedback loop involving the MEK-ERK pathway but not the FBXW7 tumour suppressor. Cell. Signal. 28, 561–571 (2016). 26898828 10.1016/j.cellsig.2016.02.009 PMC6399479

[51] J. M. Fernandes Neto, E. Nadal, E. Bosdriesz, S. N. Ooft, L. Farre, C. McLean, S. Klarenbeek, A. Jurgens, H. Hagen, L. Wang, E. Felip, A. Martinez-Marti, A. Vidal, E. Voest, L. F. A. Wessels, O. van Tellingen, A. Villanueva, R. Bernards, Multiple low dose therapy as an effective strategy to treat EGFR inhibitor-resistant NSCLC tumours. Nat. Commun. 11, 3157 (2020). 32572029 10.1038/s41467-020-16952-9 PMC7308397

[52] R. J. Sullivan, A. Hollebecque, K. T. Flaherty, G. I. Shapiro, J. Rodon Ahnert, M. J. Millward, W. Zhang, L. Gao, A. Sykes, M. D. Willard, D. Yu, A. E. Schade, K. A. Crowe, D. L. Flynn, M. D. Kaufman, J. R. Henry, S. B. Peng, K. A. Benhadji, I. Conti, M. S. Gordon, R. V. Tiu, D. S. Hong, A phase I study of LY3009120, a pan-RAF inhibitor, in patients with advanced or metastatic cancer. Mol. Cancer Ther. 19, 460–467 (2020). 31645440 10.1158/1535-7163.MCT-19-0681

[53] I. Yen, F. Shanahan, J. Lee, Y. S. Hong, S. J. Shin, A. R. Moore, J. Sudhamsu, M. T. Chang, I. Bae, D. dela Cruz, T. Hunsaker, C. Klijn, N. P. D. Liau, E. Lin, S. E. Martin, Z. Modrusan, R. Piskol, E. Segal, A. Venkatanarayan, X. Ye, J. Yin, L. Zhang, J. S. Kim, H. S. Lim, K. P. Kim, Y. J. Kim, H. S. Han, S. J. Lee, S. T. Kim, M. Jung, Y. H. Hong, Y. S. Noh, M. Choi, O. Han, M. Nowicka, S. Srinivasan, Y. Yan, T. W. Kim, S. Malek, ARAF mutations confer resistance to the RAF inhibitor belvarafenib in melanoma. Nature 594, 418–423 (2021). 33953400 10.1038/s41586-021-03515-1

[54] K. A. Monaco, S. Delach, J. Yuan, Y. Mishina, P. Fordjour, E. Labrot, D. McKay, R. Guo, S. Higgins, H. Q. Wang, J. Liang, K. Bui, J. Green, P. Aspesi, J. Ambrose, F. Mapa, L. Griner, M. Jaskelioff, J. Fuller, K. Crawford, G. Pardee, S. Widger, P. S. Hammerman, J. A. Engelman, D. D. Stuart, V. G. Cooke, G. Caponigro, LXH254, a potent and selective ARAF-sparing inhibitor of BRAF and CRAF for the treatment of MAPK-driven tumors. Clin. Cancer Res. 27, 2061–2073 (2021). 33355204 10.1158/1078-0432.CCR-20-2563

[55] R. Herr, S. Halbach, M. Heizmann, H. Busch, M. Boerries, T. Brummer, BRAF inhibition upregulates a variety of receptor tyrosine kinases and their downstream effector Gab2 in colorectal cancer cell lines. Oncogene 37, 1576–1593 (2018). 29326440 10.1038/s41388-017-0063-5

[56] C. A. Pratilas, B. S. Taylor, Q. Ye, A. Viale, C. Sander, D. B. Solit, N. Rosen, (V600E)BRAF is associated with disabled feedback inhibition of RAF-MEK signaling and elevated transcriptional output of the pathway. Proc. Natl. Acad. Sci. U.S.A. 106, 4519–4524 (2009). 19251651 10.1073/pnas.0900780106 PMC2649208

[57] J. Phuchareon, F. McCormick, D. W. Eisele, O. Tetsu, EGFR inhibition evokes innate drug resistance in lung cancer cells by preventing Akt activity and thus inactivating Ets-1 function. Proc. Natl. Acad. Sci. U.S.A. 112, E3855–E3863 (2015). 26150526 10.1073/pnas.1510733112 PMC4517222

[58] N. Gutierrez-Prat, H. L. Zuberer, L. Mangano, Z. Karimaddini, L. Wolf, S. Tyanova, L. C. Wellinger, D. Marbach, V. Griesser, P. Pettazzoni, J. R. Bischoff, D. Rohle, C. Palladino, I. Vivanco, DUSP4 protects BRAF- and NRAS-mutant melanoma from oncogene overdose through modulation of MITF. Life Sci. Alliance 5, e202101235 (2022). 35580987 10.26508/lsa.202101235 PMC9113946

[59] D. M. Molina, R. Jafari, M. Ignatushchenko, T. Seki, E. A. Larsson, C. Dan, L. Sreekumar, Y. Cao, P. Nordlund, Monitoring drug target engagement in cells and tissues using the cellular thermal shift assay. Science 341, 84–87 (2013). 23828940 10.1126/science.1233606

[60] M. Holderfield, M. M. Deuker, F. McCormick, M. McMahon, Targeting RAF kinases for cancer therapy: BRAF-mutated melanoma and beyond. Nat. Rev. Cancer 14, 455–467 (2014). 24957944 10.1038/nrc3760 PMC4250230

[61] P. I. Poulikakos, C. Zhang, G. Bollag, K. M. Shokat, N. Rosen, RAF inhibitors transactivate RAF dimers and ERK signalling in cells with wild-type BRAF. Nature 464, 427–430 (2010). 20179705 10.1038/nature08902 PMC3178447

[62] D. N. Meijles, J. J. Cull, S. T. E. Cooper, T. Markou, M. A. Hardyman, S. J. Fuller, H. O. Alharbi, Z. H. R. Haines, V. Alcantara-Alonso, P. E. Glennon, M. N. Sheppard, P. H. Sugden, A. Clerk, The anti-cancer drug dabrafenib is not cardiotoxic and inhibits cardiac remodelling and fibrosis in a murine model of hypertension. Clin. Sci. (Lond.) 135, 1631–1647 (2021). 34296750 10.1042/CS20210192 PMC8302807

[63] T. R. Rheault, J. C. Stellwagen, G. M. Adjabeng, K. R. Hornberger, K. G. Petrov, A. G. Waterson, S. H. Dickerson, R. A. Mook Jr., S. G. Laquerre, A. J. King, O. W. Rossanese, M. R. Arnone, K. N. Smitheman, L. S. Kane-Carson, C. Han, G. S. Moorthy, K. G. Moss, D. E. Uehling, Discovery of dabrafenib: A selective inhibitor of raf kinases with antitumor activity against B-Raf-driven tumors. ACS Med. Chem. Lett. 4, 358–362 (2013). 24900673 10.1021/ml4000063 PMC4027516

[64] P. Koelblinger, O. Thuerigen, R. Dummer, Development of encorafenib for BRAF-mutated advanced melanoma. Curr. Opin. Oncol. 30, 125–133 (2018). 29356698 10.1097/CCO.0000000000000426 PMC5815646

[65] X. M. Cotto-Rios, B. Agianian, N. Gitego, E. Zacharioudakis, O. Giricz, Y. Wu, Y. Zou, A. Verma, P. I. Poulikakos, E. Gavathiotis, Inhibitors of BRAF dimers using an allosteric site. Nat. Commun. 11, 4370 (2020). 32873792 10.1038/s41467-020-18123-2 PMC7462985

[66] J. Yap, R. N. V. K. Deepak, Z. Tian, W. H. Ng, K. C. Goh, A. Foo, Z. H. Tee, M. P. Mohanam, Y. R. M. Sim, U. Degirmenci, P. Lam, Z. Chen, H. Fan, J. Hu, The stability of R-spine defines RAF inhibitor resistance: A comprehensive analysis of oncogenic BRAF mutants with in-frame insertion of αC-β4 loop. Sci. Adv. 7, eabg0390 (2021). 34108213 10.1126/sciadv.abg0390 PMC8189578

[67] D. M. Hyman, I. Puzanov, V. Subbiah, J. E. Faris, I. Chau, J. Y. Blay, J. Wolf, N. S. Raje, E. L. Diamond, A. Hollebecque, R. Gervais, M. E. Elez-Fernandez, A. Italiano, R. D. Hofheinz, M. Hidalgo, E. Chan, M. Schuler, S. F. Lasserre, M. Makrutzki, F. Sirzen, M. L. Veronese, J. Tabernero, J. Baselga, Vemurafenib in multiple nonmelanoma cancers with BRAF V600 mutations. N. Engl. J. Med. 373, 726–736 (2015). 26287849 10.1056/NEJMoa1502309 PMC4971773

[68] M. A. Gouda, V. Subbiah, Precision oncology for BRAF-mutant cancers with BRAF and MEK inhibitors: From melanoma to tissue-agnostic therapy. ESMO Open 8, 100788 (2023). 36842301 10.1016/j.esmoop.2023.100788 PMC9984800

[69] M. H. Tan, N. J. Nowak, R. Loor, H. Ochi, A. A. Sandberg, C. Lopez, J. W. Pickren, R. Berjian, H. O. Douglass, T. M. Chu, Characterization of a new primary human pancreatic tumor line. Cancer Invest. 4, 15–23 (1986). 3754176 10.3109/07357908609039823

[70] L. G. Ahronian, E. M. Sennott, E. M. van Allen, N. Wagle, E. L. Kwak, J. E. Faris, J. T. Godfrey, K. Nishimura, K. D. Lynch, C. H. Mermel, E. L. Lockerman, A. Kalsy, J. M. Gurski Jr., S. Bahl, K. Anderka, L. M. Green, N. J. Lennon, T. G. Huynh, M. Mino-Kenudson, G. Getz, D. Dias-Santagata, A. J. Iafrate, J. A. Engelman, L. A. Garraway, R. B. Corcoran, Clinical acquired resistance to RAF inhibitor combinations in BRAF-mutant colorectal cancer through MAPK pathway alterations. Cancer Discov. 5, 358–367 (2015). 25673644 10.1158/2159-8290.CD-14-1518 PMC4390490

[71] K. S. M. Smalley, M. Xiao, J. Villanueva, T. K. Nguyen, K. T. Flaherty, R. Letrero, P. Van Belle, D. E. Elder, Y. Wang, K. L. Nathanson, M. Herlyn, CRAF inhibition induces apoptosis in melanoma cells with non-V600E BRAF mutations. Oncogene 28, 85–94 (2009). 18794803 10.1038/onc.2008.362 PMC2898184

[72] S. M. Wilhelm, C. Carter, L. Y. Tang, D. Wilkie, A. McNabola, H. Rong, C. Chen, X. Zhang, P. Vincent, M. McHugh, Y. Cao, J. Shujath, S. Gawlak, D. Eveleigh, B. Rowley, L. Liu, L. Adnane, M. Lynch, D. Auclair, I. Taylor, R. Gedrich, A. Voznesensky, B. Riedl, L. E. Post, G. Bollag, P. A. Trail, BAY 43-9006 exhibits broad spectrum oral antitumor activity and targets the RAF/MEK/ERK pathway and receptor tyrosine kinases involved in tumor progression and angiogenesis. Cancer Res. 64, 7099–7109 (2004). 15466206 10.1158/0008-5472.CAN-04-1443

[73] D. Strumberg, H. Richly, R. A. Hilger, N. Schleucher, S. Korfee, M. Tewes, M. Faghih, E. Brendel, D. Voliotis, C. G. Haase, B. Schwartz, A. Awada, R. Voigtmann, M. E. Scheulen, S. Seeber, Phase I clinical and pharmacokinetic study of the Novel Raf kinase and vascular endothelial growth factor receptor inhibitor BAY 43-9006 in patients with advanced refractory solid tumors. J. Clin. Oncol. 23, 965–972 (2005). 15613696 10.1200/JCO.2005.06.124

[74] A. Awada, A. Hendlisz, T. Gil, S. Bartholomeus, M. Mano, D. de Valeriola, D. Strumberg, E. Brendel, C. G. Haase, B. Schwartz, M. Piccart, Phase I safety and pharmacokinetics of BAY 43-9006 administered for 21 days on/7 days off in patients with advanced, refractory solid tumours. Br. J. Cancer 92, 1855–1861 (2005). 15870716 10.1038/sj.bjc.6602584 PMC2361774

[75] R. J. Sullivan, J. R. Infante, F. Janku, D. J. L. Wong, J. A. Sosman, V. Keedy, M. R. Patel, G. I. Shapiro, J. W. Mier, A. W. Tolcher, A. Wang-Gillam, M. Sznol, K. Flaherty, E. Buchbinder, R. D. Carvajal, A. M. Varghese, M. E. Lacouture, A. Ribas, S. P. Patel, G. A. DeCrescenzo, C. M. Emery, A. L. Groover, S. Saha, M. Varterasian, D. J. Welsch, D. M. Hyman, B. T. Li, First-in-class ERK1/2 inhibitor ulixertinib (BVD-523) in patients with MAPK mutant advanced solid tumors: Results of a phase I dose-escalation and expansion study. Cancer Discov. 8, 184–195 (2018). 29247021 10.1158/2159-8290.CD-17-1119

[76] R. Sigaud, L. Rösch, C. Gatzweiler, J. Benzel, L. von Soosten, H. Peterziel, F. Selt, S. Najafi, S. Ayhan, X. F. Gerloff, N. Hofmann, I. Büdenbender, L. Schmitt, K. I. Foerster, J. Burhenne, W. E. Haefeli, A. Korshunov, F. Sahm, C. M. van Tilburg, D. T. W. Jones, S. M. Pfister, D. Knoerzer, B. L. Kreider, M. Sauter, K. W. Pajtler, M. Zuckermann, I. Oehme, O. Witt, T. Milde, The first-in-class ERK inhibitor ulixertinib shows promising activity in mitogen-activated protein kinase (MAPK)-driven pediatric low-grade glioma models. Neuro Oncol. 25, 566–579 (2023). 35882450 10.1093/neuonc/noac183 PMC10013652

[77] M. Ghasemi, T. Turnbull, S. Sebastian, I. Kempson, The MTT assay: Utility, limitations, pitfalls, and interpretation in bulk and single-cell analysis. Int. J. Mol. Sci. 22, 12827 (2021). 34884632 10.3390/ijms222312827 PMC8657538

[78] A. Prahallad, C. Sun, S. Huang, F. di Nicolantonio, R. Salazar, D. Zecchin, R. L. Beijersbergen, A. Bardelli, R. Bernards, Unresponsiveness of colon cancer to BRAF(V600E) inhibition through feedback activation of EGFR. Nature 483, 100–103 (2012). 22281684 10.1038/nature10868

[79] R. B. Corcoran, H. Ebi, A. B. Turke, E. M. Coffee, M. Nishino, A. P. Cogdill, R. D. Brown, P. Della Pelle, D. Dias-Santagata, K. E. Hung, K. T. Flaherty, A. Piris, J. A. Wargo, J. Settleman, M. Mino-Kenudson, J. A. Engelman, EGFR-mediated re-activation of MAPK signaling contributes to insensitivity of BRAF mutant colorectal cancers to RAF inhibition with vemurafenib. Cancer Discov. 2, 227–235 (2012). 22448344 10.1158/2159-8290.CD-11-0341 PMC3308191

[80] K. Zmajkovicova, V. Jesenberger, F. Catalanotti, C. Baumgartner, G. Reyes, M. Baccarini, MEK1 is required for PTEN membrane recruitment, AKT regulation, and the maintenance of peripheral tolerance. Mol. Cell 50, 43–55 (2013). 23453810 10.1016/j.molcel.2013.01.037 PMC3625979

[81] B. A. Hemmings, D. F. Restuccia, PI3K-PKB/Akt pathway. Cold Spring Harb. Perspect. Biol. 4, a011189 (2012). 22952397 10.1101/cshperspect.a011189 PMC3428770

[82] D. Brauswetter, B. Gurbi, A. Varga, E. Várkondi, R. Schwab, G. Bánhegyi, O. Fábián, G. Kéri, I. Vályi-Nagy, I. Peták, Molecular subtype specific efficacy of MEK inhibitors in pancreatic cancers. PLOS ONE 12, e0185687 (2017). 28957417 10.1371/journal.pone.0185687 PMC5619833

[83] R. Hoefflin, A. L. Geißler, R. Fritsch, R. Claus, J. Wehrle, P. Metzger, M. Reiser, L. Mehmed, L. Fauth, D. H. Heiland, T. Erbes, F. Stock, A. Csanadi, C. Miething, B. Weddeling, F. Meiss, D. von Bubnoff, C. Dierks, I. Ge, V. Brass, S. Heeg, H. Schäfer, M. Boeker, J. Rawluk, E. M. Botzenhart, G. Kayser, S. Hettmer, H. Busch, C. Peters, M. Werner, J. Duyster, T. Brummer, M. Boerries, S. Lassmann, N. von Bubnoff, Personalized clinical decision making through implementation of a molecular tumor board: A german single-center experience. JCO Precis. Oncol. 2, 1–16 (2018). 10.1200/PO.18.00105 PMC7446498 32913998

[84] R. Kim, E. Tan, E. Wang, A. Mahipal, D. T. Chen, B. Cao, F. Masawi, C. Machado, J. Yu, D. W. Kim, A phase I trial of trametinib in combination with sorafenib in patients with advanced hepatocellular cancer. Oncologist 25, e1893–e1899 (2020). 32776632 10.1634/theoncologist.2020-0759 PMC8186409

[85] F. de Braud, C. Dooms, R. S. Heist, C. Lebbe, M. Wermke, A. Gazzah, D. Schadendorf, P. Rutkowski, J. Wolf, P. A. Ascierto, I. Gil-Bazo, S. Kato, M. Wolodarski, M. McKean, E. Muñoz Couselo, M. Sebastian, A. Santoro, V. Cooke, L. Manganelli, K. Wan, A. Gaur, J. Kim, G. Caponigro, X. M. Couillebault, H. Evans, C. D. Campbell, S. Basu, M. Moschetta, A. Daud, Initial evidence for the efficacy of naporafenib in combination with trametinib in NRAS-mutant melanoma: Results from the expansion arm of a phase Ib, open-label study. J. Clin. Oncol. 41, 2651–2660 (2023). 36947734 10.1200/JCO.22.02018

[86] M. Phadke, G. T. Gibney, C. J. Rich, I. V. Fedorenko, Y. A. Chen, R. R. Kudchadkar, V. K. Sondak, J. Weber, J. L. Messina, K. S. M. Smalley, XL888 limits vemurafenib-induced proliferative skin events by suppressing paradoxical MAPK activation. J. Invest. Dermatol. 135, 2542–2544 (2015). 26039542 10.1038/jid.2015.205 PMC4567904

[87] S. A. K. Rasheed, L. V. Subramanyan, W. K. Lim, U. K. Udayappan, M. Wang, P. J. Casey, The emerging roles of Gα12/13 proteins on the hallmarks of cancer in solid tumors. Oncogene 41, 147–158 (2022). 34689178 10.1038/s41388-021-02069-w PMC8732267

[88] J. X. Zhang, M. Yun, Y. Xu, J. W. Chen, H. W. Weng, Z. S. Zheng, C. Chen, D. Xie, S. Ye, GNA13 as a prognostic factor and mediator of gastric cancer progression. Oncotarget 7, 4414–4427 (2016). 26735177 10.18632/oncotarget.6780 PMC4826215

[89] K. O. Wrzeszczynski, S. Rahman, M. O. Frank, K. Arora, M. Shah, H. Geiger, V. Felice, D. Manaa, E. Dikoglu, D. Khaira, A. R. Chimpiri, V. V. Michelini, V. Jobanputra, R. B. Darnell, S. Powers, M. Choi, Identification of targetable BRAF ΔN486_P490 variant by whole-genome sequencing leading to dabrafenib-induced remission of a BRAF-mutant pancreatic adenocarcinoma. Cold Spring Harb. Mol. Case Stud. 5, a004424 (2019). 31519698 10.1101/mcs.a004424 PMC6913137

[90] A. D. Singhi, B. George, J. R. Greenbowe, J. Chung, J. Suh, A. Maitra, S. J. Klempner, A. Hendifar, J. M. Milind, T. Golan, R. E. Brand, A. H. Zureikat, S. Roy, A. B. Schrock, V. A. Miller, J. S. Ross, S. M. Ali, N. Bahary, Real-time targeted genome profile analysis of pancreatic ductal adenocarcinomas identifies genetic alterations that might be targeted with existing drugs or used as biomarkers. Gastroenterology 156, 2242–2253.e4 (2019). 30836094 10.1053/j.gastro.2019.02.037

[91] F. Bray, J. Ferlay, I. Soerjomataram, R. L. Siegel, L. A. Torre, A. Jemal, Global cancer statistics 2018: GLOBOCAN estimates of incidence and mortality worldwide for 36 cancers in 185 countries. CA Cancer J. Clin. 68, 394–424 (2018). 30207593 10.3322/caac.21492

[92] R. Thomas, C. A. Wiley, E. L. Droste, J. Robertson, B. A. Inman, M. Breen, Whole exome sequencing analysis of canine urothelial carcinomas without BRAF V595E mutation: Short in-frame deletions in BRAF and MAP2K1 suggest alternative mechanisms for MAPK pathway disruption. PLOS Genet. 19, e1010575 (2023). 37079639 10.1371/journal.pgen.1010575 PMC10153751

[93] A. S. Shaw, A. P. Kornev, J. Hu, L. G. Ahuja, S. S. Taylor, Kinases and pseudokinases: Lessons from RAF. Mol. Cell. Biol. 34, 1538–1546 (2014). 24567368 10.1128/MCB.00057-14 PMC3993607

[94] E. L. Diamond, B. H. Durham, G. A. Ulaner, E. Drill, J. Buthorn, M. Ki, L. Bitner, H. Cho, R. J. Young, J. H. Francis, R. Rampal, M. Lacouture, L. A. Brody, N. Ozkaya, A. Dogan, N. Rosen, A. Iasonos, O. Abdel-Wahab, D. M. Hyman, Efficacy of MEK inhibition in patients with histiocytic neoplasms. Nature 567, 521–524 (2019). 30867592 10.1038/s41586-019-1012-y PMC6438729

[95] Z. Eroglu, A. Ribas, Combination therapy with BRAF and MEK inhibitors for melanoma: Latest evidence and place in therapy. Ther. Adv. Med. Oncol. 8, 48–56 (2016). 26753005 10.1177/1758834015616934 PMC4699264

[96] I. Ozkan-Dagliyan, J. N. Diehl, S. D. George, A. Schaefer, B. Papke, K. Klotz-Noack, A. M. Waters, C. M. Goodwin, P. Gautam, M. Pierobon, S. Peng, T. S. K. Gilbert, K. H. Lin, O. Dagliyan, K. Wennerberg, E. F. Petricoin III, N. L. Tran, S. V. Bhagwat, R. V. Tiu, S. B. Peng, L. E. Herring, L. M. Graves, C. Sers, K. C. Wood, A. D. Cox, C. J. Der, Low-dose vertical inhibition of the RAF-MEK-ERK cascade causes apoptotic death of KRAS mutant cancers. Cell Rep. 31, 107764 (2020). 32553168 10.1016/j.celrep.2020.107764 PMC7393480

[97] J. E. Shin, H. J. An, H. S. Park, H. Kim, B. Y. Shim, Efficacy of dabrafenib/trametinib in pancreatic ductal adenocarcinoma with BRAF NVTAP deletion: A case report. Front. Oncol. 12, 976450 (2022). 36505826 10.3389/fonc.2022.976450 PMC9731151

[98] R. Renier, P. De Haes, F. Bosisio, I. V. Bempt, A. J. F. Woei, Vulvar Langerhans cell histiocytosis: Clinicopathologic characteristics, mutational profile, and treatment of 4 patients in a single-center cohort. JAAD Case Rep. 36, 78–81 (2023). 37250013 10.1016/j.jdcr.2023.03.024 PMC10220460

[99] S. Zhang, Z. Yang, Y. Cheng, X. Guo, C. Liu, S. Wang, L. Zhang, BRAF L485-P490 deletion mutant metastatic melanoma sensitive to BRAF and MEK inhibition: A case report and literature review. Front. Pharmacol. 13, 1019217 (2022). 36686670 10.3389/fphar.2022.1019217 PMC9853440

[100] J. Oberoi, X. A. Guiu, E. A. Outwin, P. Schellenberger, T. I. Roumeliotis, J. S. Choudhary, L. H. Pearl, HSP90-CDC37-PP5 forms a structural platform for kinase dephosphorylation. Nat. Commun. 13, 7343 (2022). 36446791 10.1038/s41467-022-35143-2 PMC9709061

[101] S. García-Alonso, P. Mesa, L. de la Puente Ovejero, G. Aizpurua, C. G. Lechuga, E. Zarzuela, C. M. Santiveri, M. Sanclemente, J. Muñoz, M. Musteanu, R. Campos-Olivas, J. Martínez-Torrecuadrada, M. Barbacid, G. Montoya, Structure of the RAF1-HSP90-CDC37 complex reveals the basis of RAF1 regulation. Mol. Cell 82, 3438–3452.e8 (2022). 36055235 10.1016/j.molcel.2022.08.012

[102] D. Keramisanou, M. V. Vasantha Kumar, N. Boose, R. R. Abzalimov, I. Gelis, Assembly mechanism of early Hsp90-Cdc37-kinase complexes. Sci. Adv. 8, eabm9294 (2022). 35294247 10.1126/sciadv.abm9294 PMC8926337

[103] D. M. Bjorklund, R. M. L. Morgan, J. Oberoi, K. L. I. M. Day, P. A. Galliou, C. Prodromou, Recognition of BRAF by CDC37 and reevaluation of the activation mechanism for the class 2 BRAF-L597R mutant. Biomolecules 12, 905 (2022). 35883461 10.3390/biom12070905 PMC9313131

[104] P. Horak, B. Klink, C. Heining, S. Gröschel, B. Hutter, M. Fröhlich, S. Uhrig, D. Hübschmann, M. Schlesner, R. Eils, D. Richter, K. Pfütze, C. Geörg, B. Meißburger, S. Wolf, A. Schulz, R. Penzel, E. Herpel, M. Kirchner, A. Lier, V. Endris, S. Singer, P. Schirmacher, W. Weichert, A. Stenzinger, R. F. Schlenk, E. Schröck, B. Brors, C. von Kalle, H. Glimm, S. Fröhling, Precision oncology based on omics data: The NCT Heidelberg experience. Int. J. Cancer 141, 877–886 (2017). 28597939 10.1002/ijc.30828

[105] G. M. O’Kane, B. T. Grünwald, G.-H. Jang, M. Masoomian, S. Picardo, R. C. Grant, R. E. Denroche, A. Zhang, Y. Wang, B. Lam, P. M. Krzyzanowski, I. M. Lungu, J. M. S. Bartlett, M. Peralta, F. Vyas, R. Khokha, J. Biagi, D. Chadwick, S. Ramotar, S. Hutchinson, A. Dodd, J. M. Wilson, F. Notta, G. Zogopoulos, S. Gallinger, J. J. Knox, S. E. Fischer, GATA6 expression distinguishes classical and basal-like subtypes in advanced pancreatic cancer. Clin. Cancer Res. 26, 4901–4910 (2020). 32156747 10.1158/1078-0432.CCR-19-3724

[106] K. L. Aung, S. E. Fischer, R. E. Denroche, G. H. Jang, A. Dodd, S. Creighton, B. Southwood, S. B. Liang, D. Chadwick, A. Zhang, G. M. O'Kane, H. Albaba, S. Moura, R. C. Grant, J. K. Miller, F. Mbabaali, D. Pasternack, I. M. Lungu, J. M. S. Bartlett, S. Ghai, M. Lemire, S. Holter, A. A. Connor, R. A. Moffitt, J. J. Yeh, L. Timms, P. M. Krzyzanowski, N. Dhani, D. Hedley, F. Notta, J. M. Wilson, M. J. Moore, S. Gallinger, J. J. Knox, Genomics-driven precision medicine for advanced pancreatic cancer: Early results from the COMPASS trial. Clin. Cancer Res. 24, 1344–1354 (2018). 29288237 10.1158/1078-0432.CCR-17-2994 PMC5968824

[107] N. A. Pham, N. Radulovich, E. Ibrahimov, S. N. Martins-Filho, Q. Li, M. Pintilie, J. Weiss, V. Raghavan, M. Cabanero, R. E. Denroche, J. M. Wilson, C. Metran-Nascente, A. Borgida, S. Hutchinson, A. Dodd, M. Begora, D. Chadwick, S. Serra, J. J. Knox, S. Gallinger, D. W. Hedley, L. Muthuswamy, M. S. Tsao, Patient-derived tumor xenograft and organoid models established from resected pancreatic, duodenal and biliary cancers. Sci. Rep. 11, 10619 (2021). 34011980 10.1038/s41598-021-90049-1 PMC8134568

[108] L. A. Baker, H. Tiriac, D. A. Tuveson, Generation and culture of human pancreatic ductal adenocarcinoma organoids from resected tumor specimens. Methods Mol. Biol. 1882, 97–115 (2019). 30378047 10.1007/978-1-4939-8879-2_9

[109] S. F. Boj, C. I. Hwang, L. A. Baker, D. D. Engle, D. A. Tuveson, H. Clevers, Model organoids provide new research opportunities for ductal pancreatic cancer. Mol. Cell. Oncol. 3, e1014757 (2016). 27308531 10.1080/23723556.2015.1014757 PMC4845167

[110] G. Galabova-Kovacs, D. Matzen, D. Piazzolla, K. Meissl, T. Plyushch, A. P. Chen, A. Silva, M. Baccarini, Essential role of B-Raf in ERK activation during extraembryonic development. Proc. Natl. Acad. Sci. U.S.A. 103, 1325–1330 (2006). 16432225 10.1073/pnas.0507399103 PMC1360532

[111] J. Albers, C. Danzer, M. Rechsteiner, H. Lehmann, L. P. Brandt, T. Hejhal, A. Catalano, P. Busenhart, A. F. Gonçalves, S. Brandt, P. K. Bode, B. Bode-Lesniewska, P. J. Wild, I. J. Frew, A versatile modular vector system for rapid combinatorial mammalian genetics. J. Clin. Invest. 125, 1603–1619 (2015). 25751063 10.1172/JCI79743 PMC4396471

[112] J. Cox, M. Mann, MaxQuant enables high peptide identification rates, individualized p.p.b.-range mass accuracies and proteome-wide protein quantification. Nat. Biotechnol. 26, 1367–1372 (2008). 19029910 10.1038/nbt.1511

[113] N. P. D. Liau, A. Venkatanarayan, J. G. Quinn, W. Phung, S. Malek, S. G. Hymowitz, J. Sudhamsu, Dimerization induced by C-terminal 14-3-3 binding is sufficient for BRAF kinase activation. Biochemistry 59, 3982–3992 (2020). 32970425 10.1021/acs.biochem.0c00517

[114] B. Zhang, Y. Chen, P. Dai, H. Yu, J. Ma, C. Chen, Y. Zhang, Y. Guan, R. Chen, T. Liu, J. Wang, L. Yang, X. Yi, X. Xia, H. Ma, Oncogenic mutations within the beta3-alphaC loop of EGFR/ERBB2/BRAF/MAP2K1 predict response to therapies. Mol. Genet. Genomic Med. 8, e1395 (2020). 32757330 10.1002/mgg3.1395 PMC7549570

[115] B. S. White, I. Lanc, J. O’Neal, H. Gupta, R. S. Fulton, H. Schmidt, C. Fronick, E. A. Belter Jr., M. Fiala, J. King, G. J. Ahmann, M. DeRome, E. R. Mardis, R. Vij, J. F. DiPersio, J. Levy, D. Auclair, M. H. Tomasson, A multiple myeloma-specific capture sequencing platform discovers novel translocations and frequent, risk-associated point mutations in IGLL5. Blood Cancer J. 8, 35 (2018). 29563506 10.1038/s41408-018-0062-y PMC5862875

[116] M. Mirdita, K. Schütze, Y. Moriwaki, L. Heo, S. Ovchinnikov, M. Steinegger, ColabFold: Making protein folding accessible to all. Nat. Methods 19, 679–682 (2022). 35637307 10.1038/s41592-022-01488-1 PMC9184281

