# Supplementary material for: BRAFΔβ3-αC in-frame deletion mutants differ in their dimerization propensity, HSP90 dependence, and druggability
Source: Sci Adv. 2023 Sep 1;9(35):eade7486. doi: 10.1126/sciadv.ade7486 (PMC11804575; doi:10.1126/sciadv.ade7486)
Supplement: Supplementary file 1 — Figs. S1 to S16 Table S1 References [file sciadv.ade7486_sm.pdf]

Science Advances
AAAS

Supplementary Materials for

BRAF$^{\Delta\beta3\text{-}\alpha C}$ in-frame deletion mutants differ in their dimerization propensity, HSP90 dependence, and druggability

Manuel Lauinger *et al.*

Corresponding author: Tilman Brummer, tilman.brummer@mol-med.uni-freiburg.de

Sci. Adv. **9**, eade7486 (2023)
DOI: 10.1126/sciadv.ade7486

This PDF file includes:

Figs. S1 to S16
Table S1
References

Supplementary Figure S1

A

β3 αC<br>480 485 490 495 500 505<br>**VAVKMLNVTAPTPQQLQAFKNEVGVLRKTR**	Net Deletion	Abbreviation In this study	Cell Line
VAVK---VTAPTPQQLQAFKNEVGVLRKTR [c]	3		
VAVKMLK---PTPQQLQAFKNEVGVLRKTR	3		
VAVKMF---APTPQQLQAFKNEVGVLRKTR	3	ΔLNVT>F	
VAVKMFS----TPQQLQAFKNEVGVLRKTR	4	delinsFS	
VAVKMF-----TPQQLQAFKNEVGVLRKTR	5	ΔLNVTAP>F	
VAVKMY-----TPQQLQAFKNEVGVLRKTR [a,b]	5	ΔLNVTAP>Y	NCI-H2405 [a,b]
VAVKML-----TPQQLQAFKNEVGVLRKTR [a,b,c,d]	5	ΔNVTAP	OV-90 [a,b]
VAVKMLK-----PQQLQAFKNEVGVLRKTR	5		
VAVKMLN-----PQQLQAFKNEVGVLRKTR	5		
VAVKMLNA-----QQLQAFKNEVGVLRKTR [b]	5	ΔVTAPTP>A	BxPC3 [b]
VAVKMLNV-----QQLQAFKNEVGVLRKTR [a]	5		
VAVKMLNVK-----QLQAFKNEVGVLRKTR	5		
VAVKMLNVTA-----LQAFKNEVGVLRKTR [a]	5		
VAVKM------TPQQLQAFKNEVGVLRKTR [b]	6		
VAVKML------PQQLQAFKNEVGVLRKTR [c]	6		

B

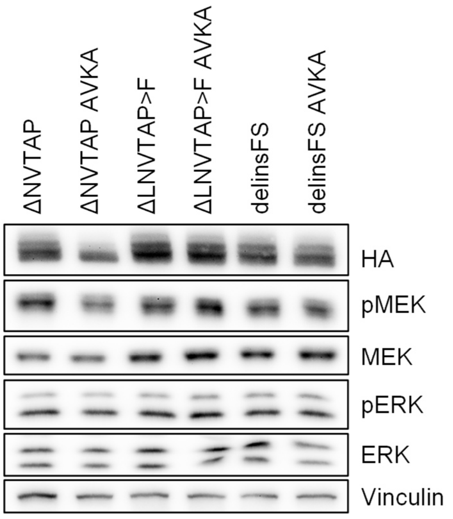

C

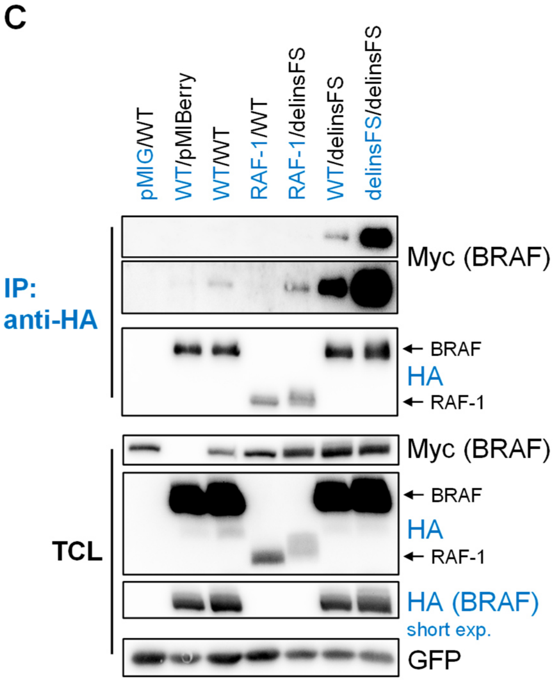

Fig. S1. (A) Overview on BRAF$^{\Delta\beta3\text{-}\alpha C}$ mutants. The table, which is based on a summary (*24*) compiled by Yuan et al. (2018) was extended by recently described variants (*39, 114, 115*) and the two mutants first characterized in this study. The mutants analyzed in this study are highlighted by blue shading. Mutants and corresponding cell lines that were previously characterized elsewhere are indicated as follows: [a] Foster et al. (*36*), [b] Chen et al. (*35*), [c] Yuan et al. (*24*), [d] Yap et al. (*66*). (**B**) HEK293T cells transiently expressing BRAF$^{\Delta\beta3\text{-}\alpha C}$ and the corresponding AVKA mutants were lysed 48 h post transfection. Total cellular lysates (TCLs) were analysed by Western blotting using the indicated antibodies. Images are representative for two independent experiments. (**C**) BRAF^{delinsFS} dimerizes with RAF-1, BRAF^{WT} or with itself (accompanies **Fig. 1**). HEK293T cells were co-transfected with pMIG/HA-BRAF (blue) or pMIBERRY/Myc-BRAF or -RAF1 (black) constructs, which encode the indicated HA- or Myc-tagged proteins, respectively. BRAF complexes were precipitated with HA antibodies. Immunoprecipitates and to TCLs (as input control) were analyzed via immunoblot using the indicated antibodies. Images are representative for two independent experiments.

Supplementary Figure S2

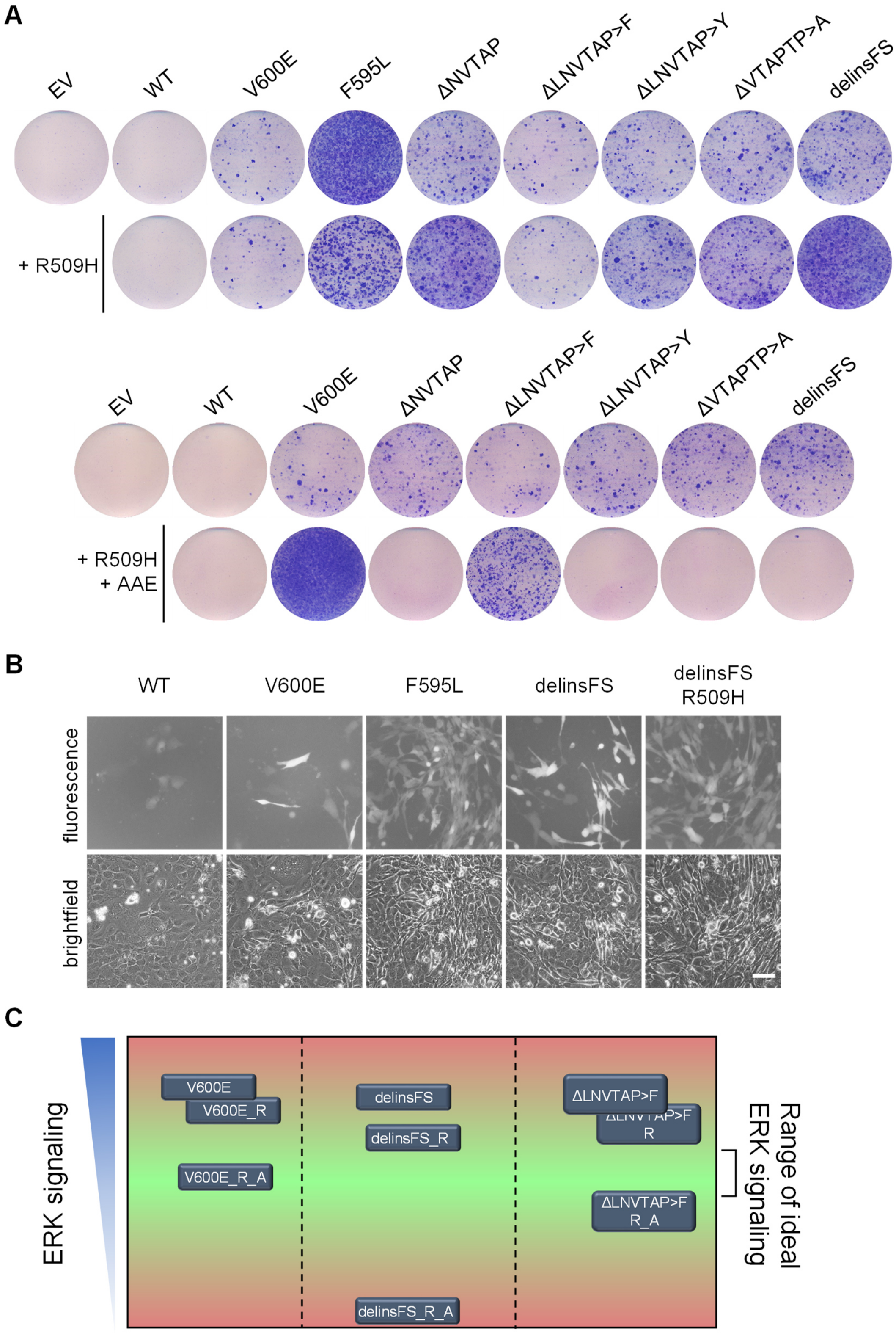

Fig. S2 (accompanies Fig. 2). The oncogenic potential of BRAF$^{\Delta\beta3\text{-}\alpha C}$ mutants is strongly modulated by dimerization. For focus formation assays, immortalized MEFs were infected with retroviral vectors encoding the indicated BRAF proteins, cultured for 14 days and stained with Giemsa reagent. Images are representative for at least three independent

experiments. The high activity $BRAF^{V600E}$ and intermediate activity $BRAF^{F595L}$ mutants serve as positive controls for MEF transformation (*44*). Of note, the number of foci produced by $BRAF^{F595L}$ was higher than for $BRAF^{V600E}$ and agrees with our previous observations showing that $BRAF^{F595L}$ with its moderate MEK/ERK phosphorylation potential delivers a signal more compatible with proliferation than the high activity $BRAF^{V600E}$ mutant known to trigger cell cycle deceleration or even arrest in certain settings (*44*). The observation that intermediate activity BRAF mutants hit the "sweet spot" of proliferation in a contact-inhibited monolayer needs to be considered when assessing the effects of the R509H and AAE mutations on the transformation potential of the $BRAF^{V600E}$ and $BRAF^{\Delta\beta3\text{-}\alpha C}$ mutants. (**B**) Micrographs of MEFs transduced with the indicated BRAF proteins. Photos were taken seven days after transduction. The IRES-coupled co-expression of HA-BRAF and GFP allows the identification of transduced fibroblasts (*9*). Scale bar: 100 µM. (**C**) Cartoon illustrating how the focus formation potential of BRAF variants is related to their signaling output. $BRAF^{V600E}$, $BRAF^{delinsFS}$, $BRAF^{\Delta LNVTAP>F}$, and their corresponding dimerization-impaired mutants (R = R509H; A = AAE) are ordered from bottom to top based on their signaling output (low to high). The range of ERK signaling ideal for proliferation is indicated by green shading.

Supplementary Figure S3

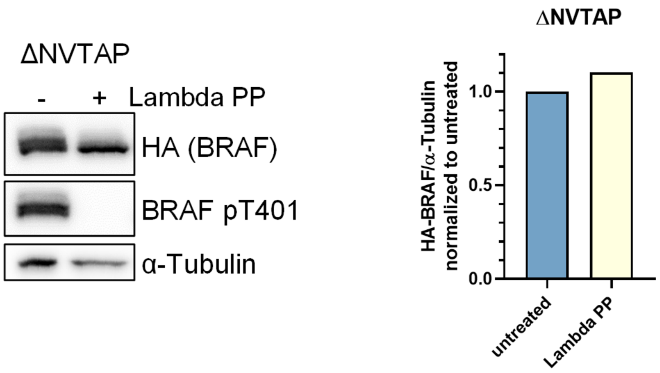

Fig. S3 (accompanies Fig. 2). Varying band shapes caused by electrophoretic mobility shifts do not confound the quantification of Western blot signals. Lysates of HEK293T cells transiently expressing HA-BRAF$^{\Delta NVTAP}$ (**Fig. 2A**) were dephosphorylated using Lambda protein phosphatase (NEB, manufacturer's protocol). Subsequently, lysates were analyzed by Western blot and quantified. Membranes were additionally probed against BRAF phosphorylated threonine 401 (pT401) to monitor successful dephosphorylation.

Supplementary Figure S4

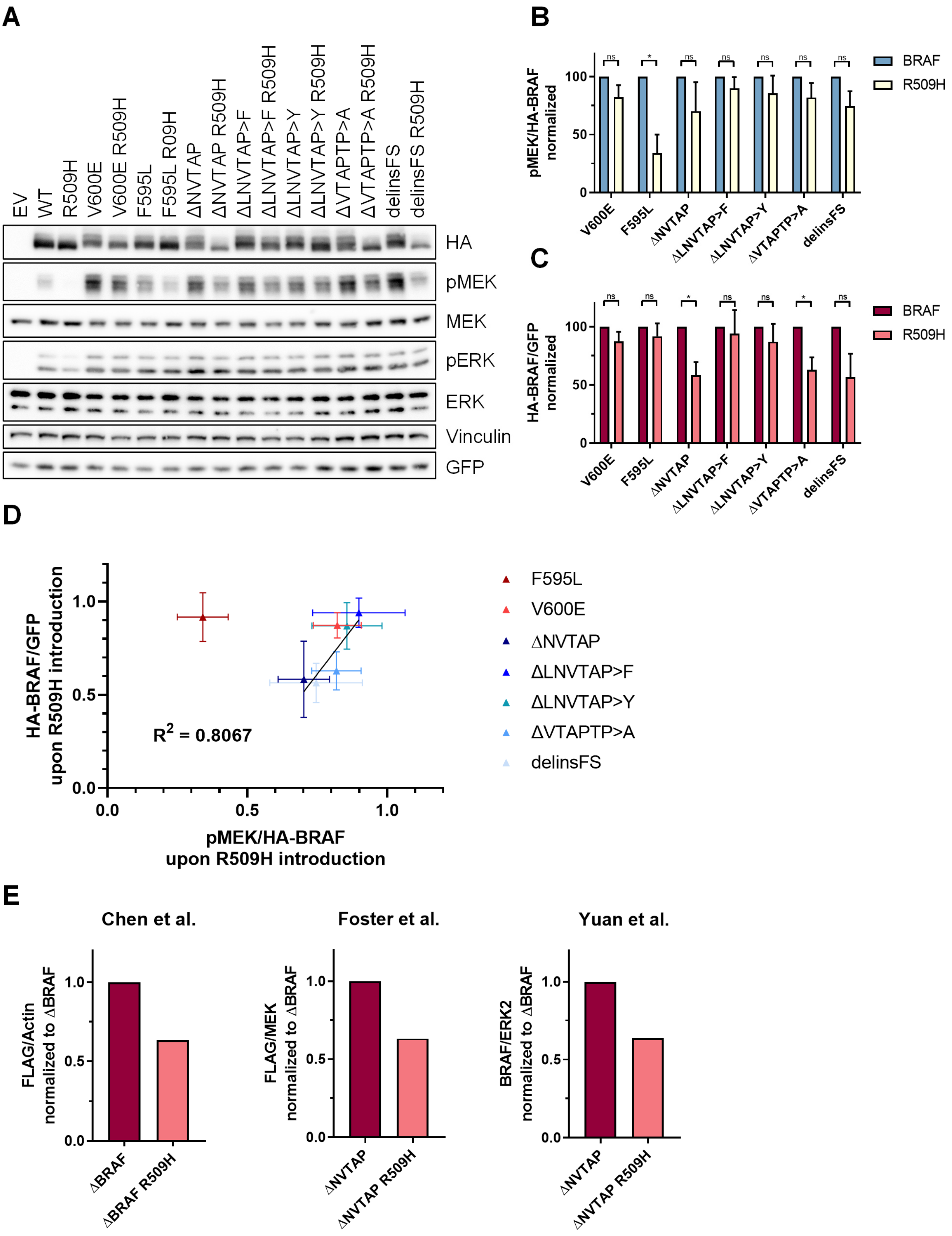

Fig. S4 (accompanies Fig. 2). In contrast to the DIF dependent class II BRAF^{F595L} mutant, the activity and stability of BRAF$^{\Delta\beta3\text{-}\alpha C}$ mutants are correlated through their dimerization dependency. (**A**) HEK293T cells were transiently transfected with the indicated HA-BRAF expression vectors. TCLs were analyzed by immunoblot using the indicated antibodies.

Images are representative for three independent experiments. (**B-C**) Immunoblots were quantified using ImageJ. Data were normalized to the respective BRAF protein without R509H mutation. Statistical analysis: mean + SD, n = 3, unpaired t-tests with Holm-Šídák correction for multiple comparisons, * $P \leq 0.05$, ** $P \leq 0.01$, *** $P \leq 0.001$, **** $P \leq 0.0001$. (**D**) Linear regression of activity (pMEK/HA-BRAF) and stability (HA-BRAF/GFP) of $BRAF^{\Delta\beta3\text{-}\alpha C}$ mutants upon R509H introduction. Calculated R^2 is indicated. $BRAF^{V600E}$ was included as a reference point. (**E**) Densitometric analysis of published Western blot data regarding the effect of the R509H substitution on BRAF expression levels (Chen *et al.:* Fig. 2C; Foster *et al.*: Fig. 2B; Yuan *et al.*: Fig. 4C). Of note, images for quantification were extracted from PDF files. (*24, 35, 36*)

Supplementary Figure S5

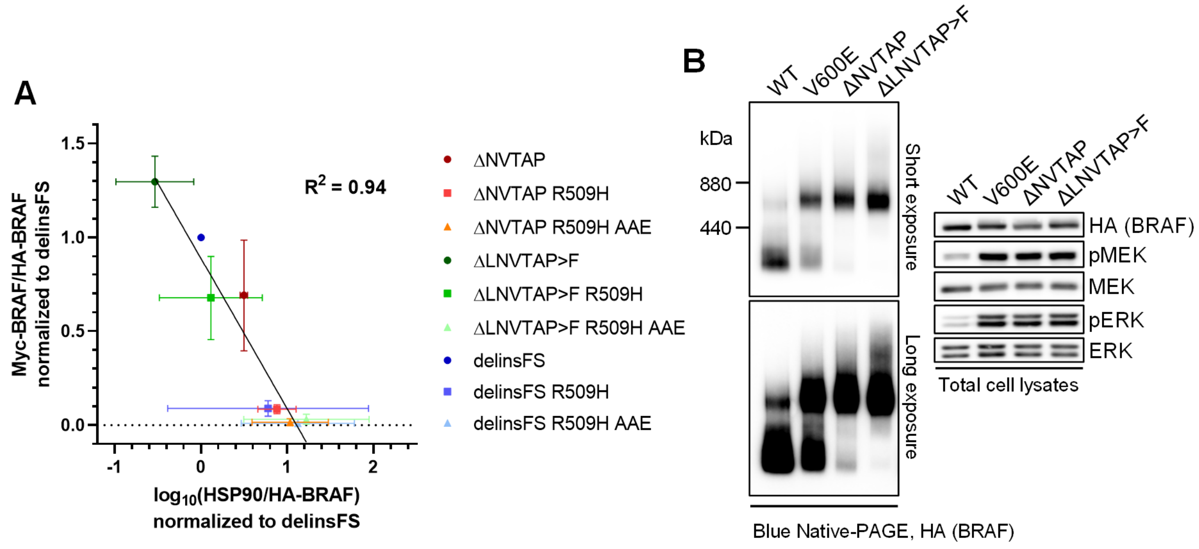

Fig. S5 (accompanies Fig. 3). (**A**) Non-linear regression of co-purified Myc-BRAF proteins and HSP90 from **Fig. 3 (A to D)**. Calculated R^2 is indicated. (**B**) Lysates of HEK293T cells expressing the indicated HA-tagged BRAF proteins were analyzed by Western blotting following BN-PAGE (left) and SDS-PAGE (right). TCLs confirm comparable expression levels of the BRAF proteins in question. The signaling output was assessed by detecting MEK and ERK phosphorylation. Representative images for two biological replicates are shown (experiment separate to that shown in **Fig. 3**).

Supplementary Figure S6

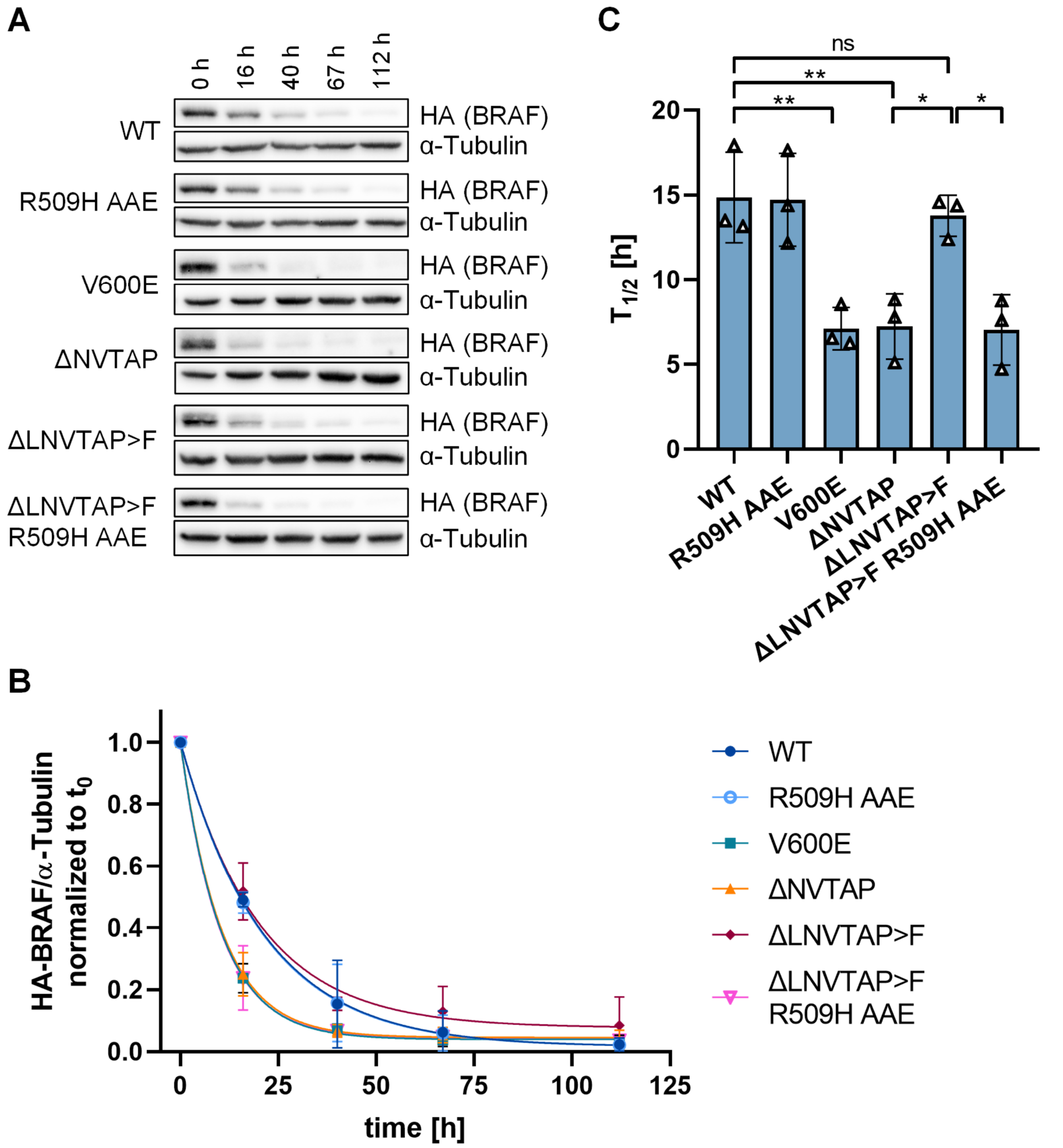

Fig. S6 (accompanies Fig. 4). MEFs transduced with lentiviral constructs for the indicated BRAF proteins were grown in the presence of tetracycline (tet). Following tet washout to stop transcription of the BRAF expression cassette and a waiting time of 26 h to allow for depletion of residual tet and tet-induced mRNA, cells were grown for the indicated times and subject to (**A**) Western blot analysis. (**B**) HA-BRAF levels were normalized to α-Tubulin and plotted over time. Graph shows mean ± SD at each time point. 0 h = 26 h post washout (**C**) Bar graph shows half-lives of BRAF proteins separately determined for each replicate. Statistical analysis: mean ± SD, $n = 3$, one-way ANOVA with Tukey's test for multiple comparisons, * $P \leq 0.05$, ** $P \leq 0.01$, *** $P \leq 0.001$, **** $P \leq 0.0001$.

Supplementary Figure S7

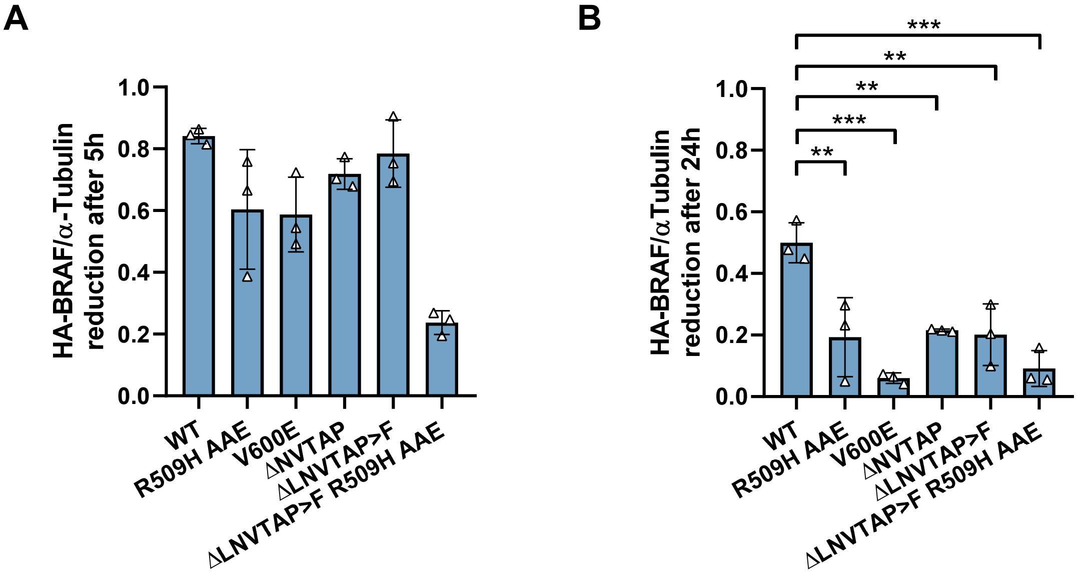

Fig S7 (accompanies Fig. 4). Reduction of BRAF levels after 5h (**A**) or 24h (**B**) of HSP90 inhibition. Data generation and analysis as described for **Fig. 4B**.

Supplementary Figure S8

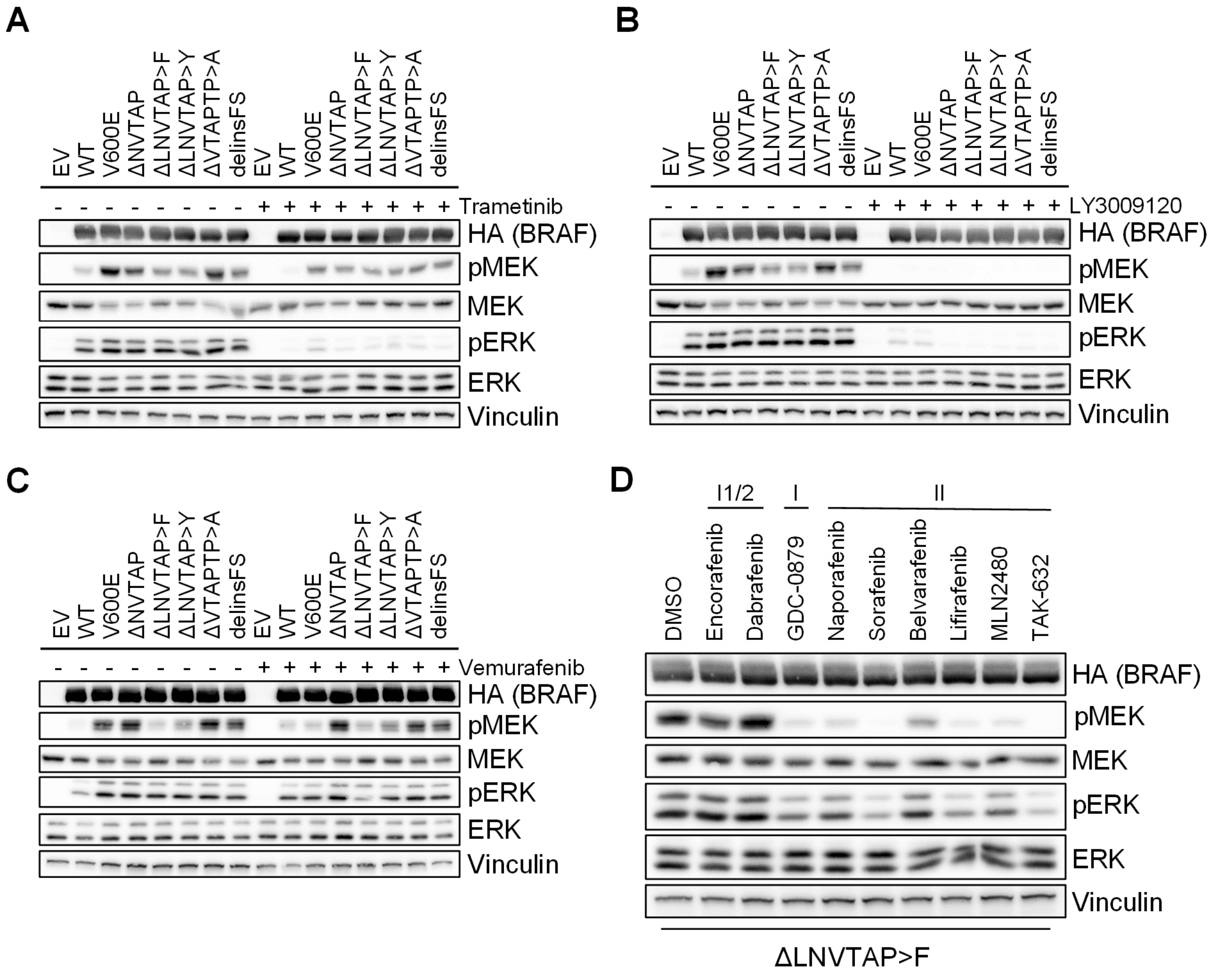

Fig S8 (accompanies Fig. 5). The signaling output of BRAF$^{\Delta\beta3\text{-}\alpha C}$ mutants can be blocked by trametinib and type II inhibitors, while type I$^{1/2}$ inhibitors show little to no activity. The indicated HA-BRAF-proteins were transiently expressed in HEK293T cells. Prior to lysis, cells were treated with (**A**) 1 µM trametinib, (**B**) 1µM LY3009120 and (**C**) 3 µM vemurafenib, or vehicle control (DMSO) for 4h. The lysates were analyzed by immunoblot using the indicated antibodies. (**D**) The Type I$^{1/2}$ RAF inhibitor-insensitive mutant BRAF$^{\Delta LNVTAP>F}$ is successfully inhibited by type I and II (αC-IN) inhibitors. HEK293T cells transiently expressing BRAF$^{\Delta LNVTAP>F}$ were treated with the indicated inhibitors for 4h, followed by lysis and Western blot analysis with the indicated antibodies. Inhibitors were used with a final concentration of 1 µM, except for encorafenib (0.5 µM), sorafenib (10 µM) and MLN2480 (3 µM). RAFi types are indicated. Drug concentrations were chosen based on literature research and previous experience. Images are representative for three independent experiments.

Supplementary Figure S9

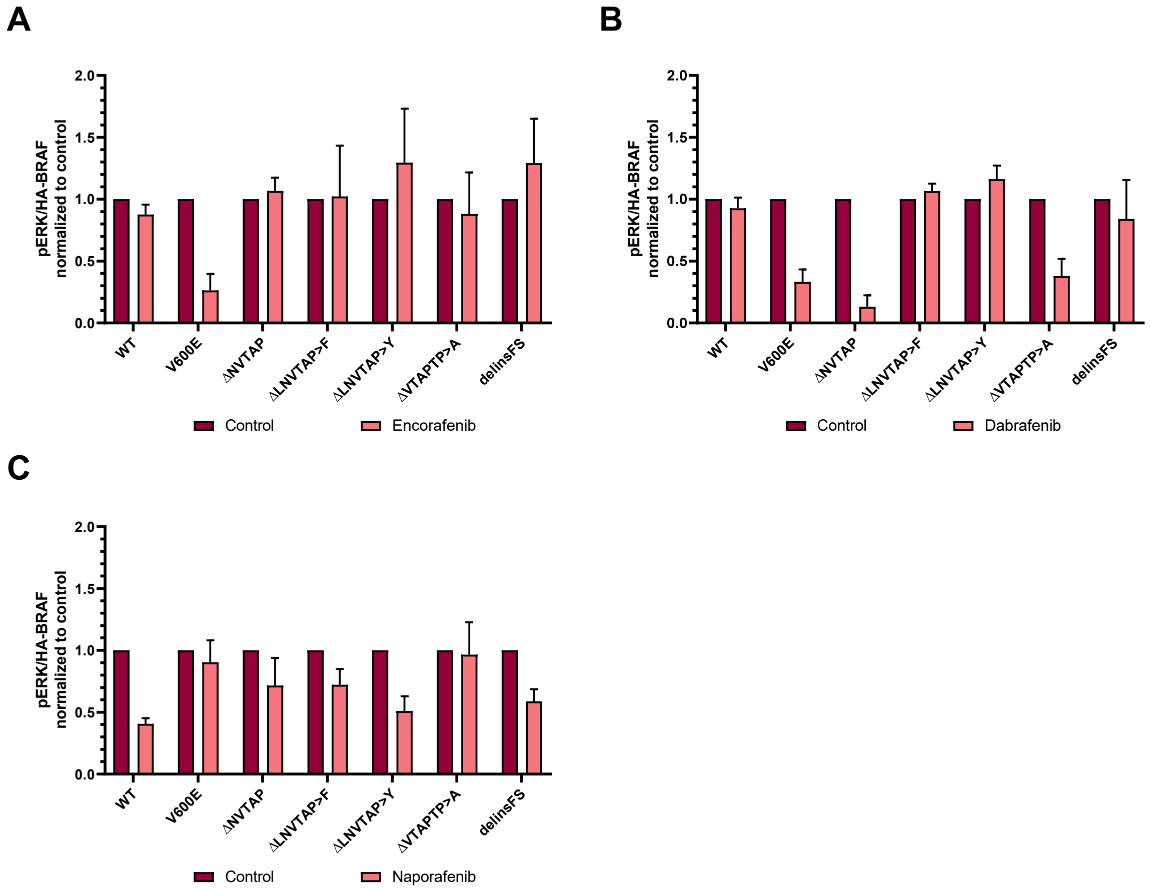

Fig S9 (accompanies Fig. 5). Quantification of phospho-ERK levels (corresponding to Western blots shown in **Fig. 5**).

Supplementary Figure S10

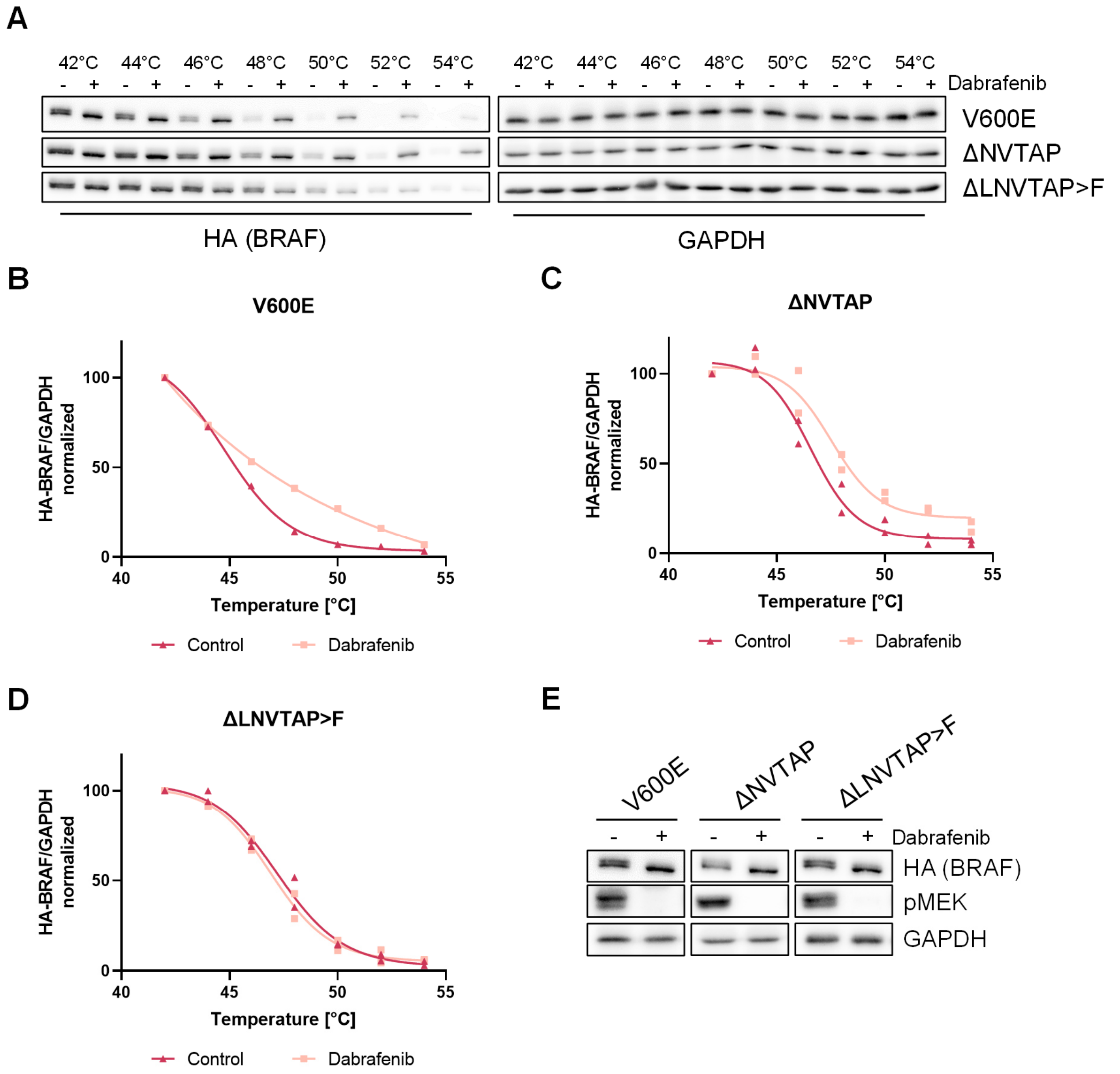

Fig S10. (A) Thermal stability of BRAF mutants in the presence of dabrafenib, assessed by CETSA. HEK293T cells transiently expressing the indicated HA-BRAF proteins were treated with 100 µM dabrafenib or control (DMSO) for 4h. (**A**) Levels of remaining native BRAF protein at increasing temperature (42-54°C for 3 min) were determined by Western blot. Representative images from two independent experiments are shown. (**B-D**) Temperature-dependent BRAF levels were corrected to loading (GAPDH), normalized to levels at 42°C and plotted over temperature with corresponding fitted curves. Graphs show individual values of two independent experiments (V600E: n=1). (**E**) phospho-MEK levels as readout for BRAF activity.

Supplementary Figure S11

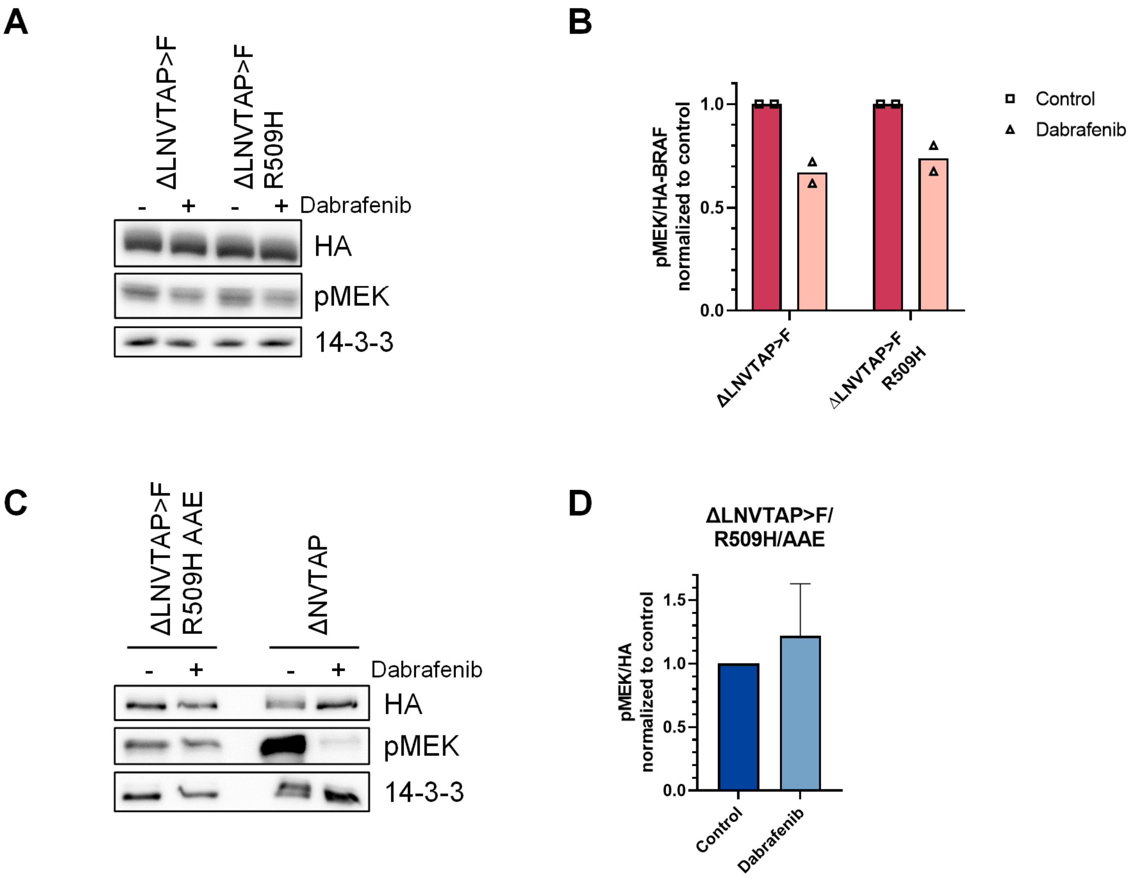

Fig. S11. (**A**) HEK293T cells transiently expressing HA-BRAF$^{\Delta LNVTAP>F}$ with or without reduced dimer affinity (R509H) were cultivated in the presence of dabrafenib (1 µM) or vehicle control (DMSO) for 4h prior to lysis and Western blot analysis. Detection of 14-3-3 proteins served as loading control. (**B**) Immunoblots were quantified using ImageJ. Bar graphs show calculated fold changes (inhibitor/control) of phospho-MEK levels normalized to the respective BRAF protein. Shown are mean and individual values, n = 2. (**C**) Experimental set-up as in (A) except that the R509H/AAE double DIF mutation was introduced into BRAF$^{\Delta LNVTAP>F}$. (**D**) shows the mean + SD of three biological replicates.

Supplementary Figure S12

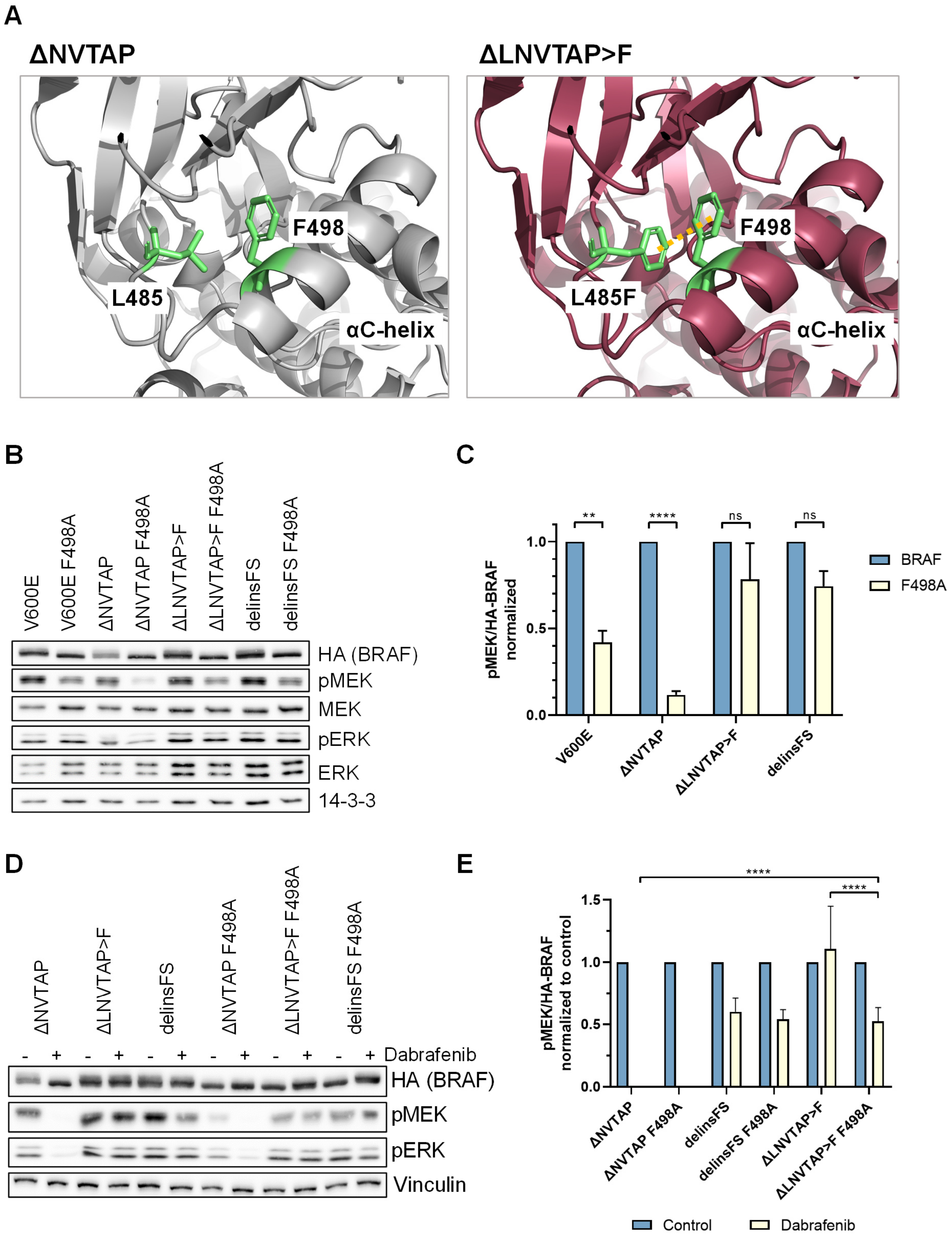

Fig. S12. Effect of F498A introduction on BRAF mutant activity and dabrafenib resistance. (**A**) Ribbon diagrams of BRAF$^{\Delta NVTAP}$ and BRAF$^{\Delta LNVTAP>F}$. The potential aromatic π-π interactions of F485 with F498 are indicated by an orange dotted line. Protein structures were modeled using alphaFold2 (*116*). (**B**) TCLs of HEK293T cells transiently expressing the indicated HA-BRAF proteins were analyzed by immunoblot. Detection of 14-

3-3 proteins served as loading control. (**B**) Immunoblots were quantified using ImageJ. Data were normalized to the respective BRAF protein without F498A mutation. Statistical analysis: mean + SD, n = 3, unpaired t-tests with Holm-Šídák correction for multiple comparisons, * $P \leq 0.05$, ** $P \leq 0.01$, *** $P \leq 0.001$, **** $P \leq 0.0001$. (**C**) HEK293T cells transiently expressing the indicated BRAF-proteins were cultivated in the presence of dabrafenib (1 µM) or vehicle control (DMSO) for 4h prior to lysis and Western blot analysis. (**D**) Quantification of immunoblots. Bar graphs show calculated fold changes (inhibitor/control) of phospho-MEK levels normalized to HA-BRAF. Statistical analysis: mean + SD, n = 3, two-way ANOVA with Holm-Šídák correction for multiple comparisons, * $P \leq 0.05$, ** $P \leq 0.01$, *** $P \leq 0.001$, **** $P \leq 0.0001$.

Supplementary Figure S13

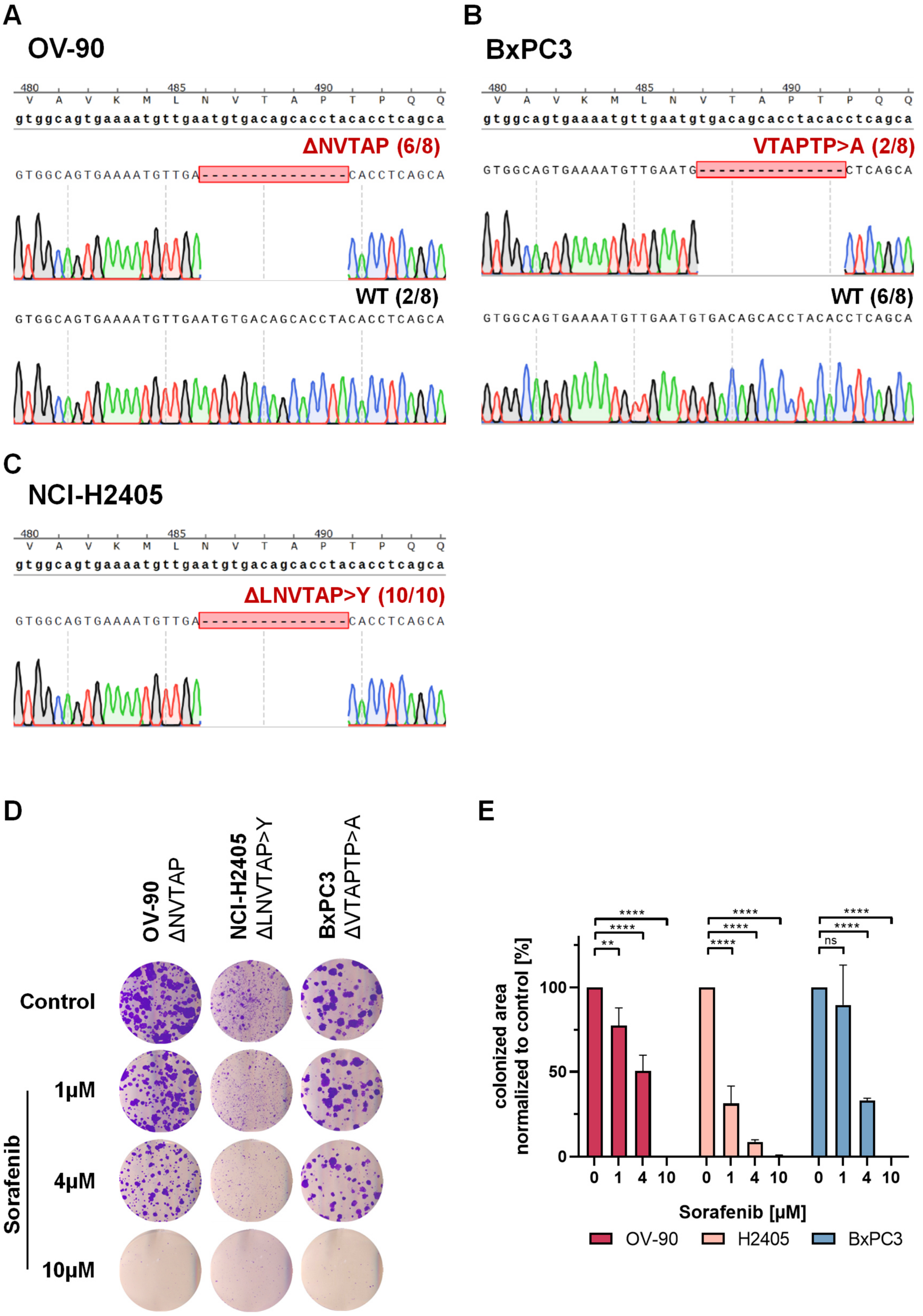

Fig. S13 (accompanies Fig. 6). The growth of cell lines expressing different endogenous BRAF$^{\Delta\beta3\text{-}\alpha C}$ mutants is blocked by sorafenib in colony formation assays. (**A - C**) **Validation of BRAF in-frame deletions in OV-90, BxPC3 and NCI-H2405 cells by Sanger sequencing.** BRAF exon 12 was amplified from extracted gDNA and cloned into the pSC-A vector. Picking single bacteria colonies enabled separate analysis of each allele of

potentially heterozygous BRAF mutants. Homozygous (NCI-H2405) or heterozygous (OV-90, BxPC3) BRAF deletions were confirmed. Sequence counts are indicated in parentheses. (**D**) Cells were cultivated in the presence of sorafenib at indicated concentrations or vehicle control (DMSO). OV-90, NCI-H2405 and BxPC3 cells were fixed and stained with crystal violet after 16, 21 or 18 days, respectively. (**E**) The colonized area was determined using ImageJ. Bar graphs show the colonized area of inhibitor-treated cells normalized to the area of those treated with vehicle control. Statistical analysis: mean + SD, n = 3, two-way ANOVA with Dunnett's test for multiple comparisons, * $P \leq 0.05$, ** $P \leq 0.01$, *** $P \leq 0.001$, **** $P \leq 0.0001$.

Supplementary Figure S14

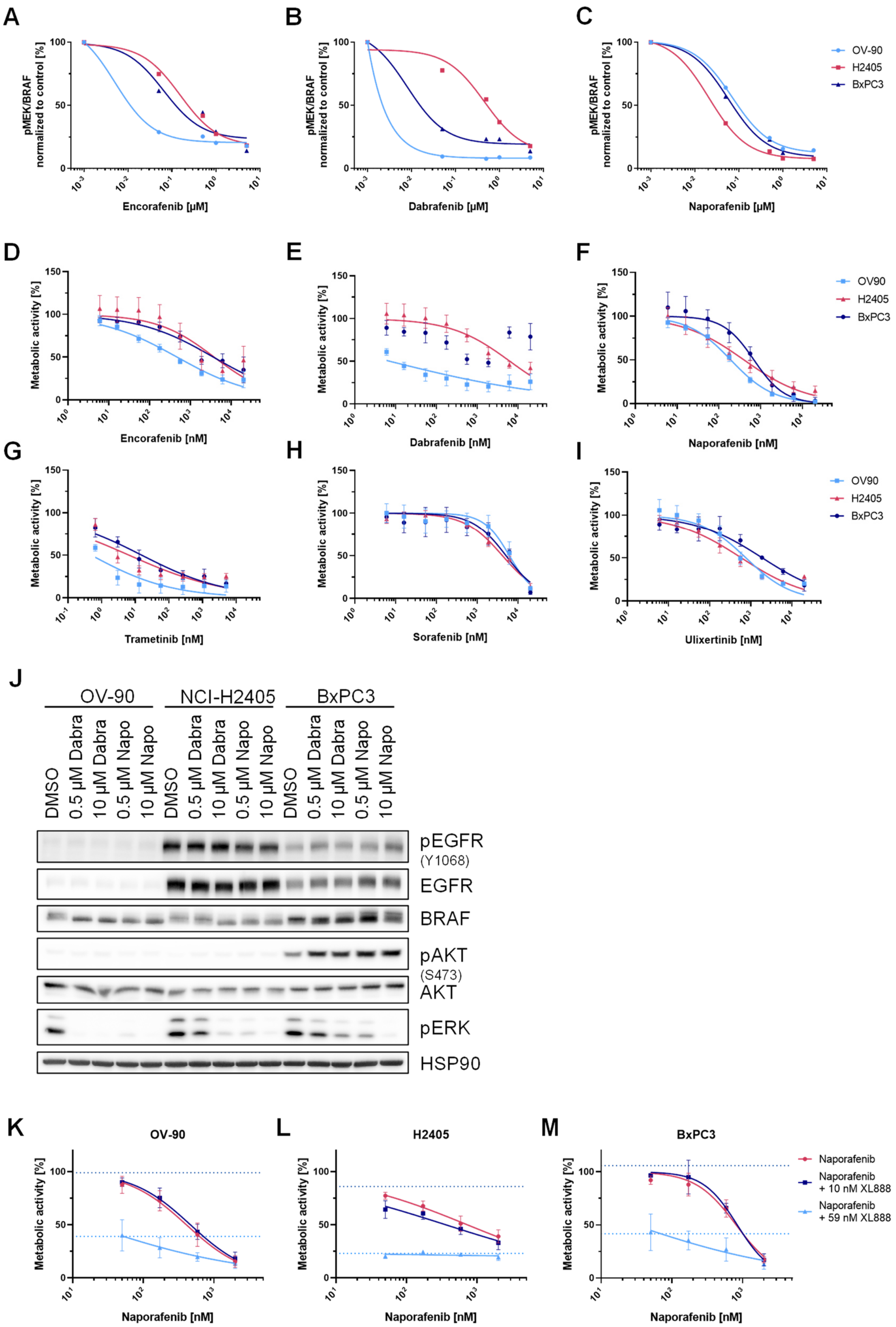

Fig. S14. (**A-C, accompanying Figs. 6 and 7**) Quantitative assessment of the effect on RAFi on MEK phosphorylation (shown in **Fig. 6, F to H**), the most direct readout for BRAF activity. Graphs show the mean of two independent experiments with corresponding fitted curves. (**D-I**) Metabolic activity of BRAF$^{\Delta\beta3\text{-}\alpha C}$ mutant-expressing cancer cell lines incubated

with inhibitor or DMSO (vehicle control) at the indicated concentrations for 96 h, followed by XTT assay (mean + SD, n = 3). Non-linear fitted curves were calculated using GraphPad Prism 9. (**J**) Expression levels and phosphorylation status of EGFR and AKT in RAF inhibitor-treated cancer cell lines. Cells were incubated with DMSO, dabrafenib or naporafenib at the indicated concentrations for 4 h, followed by lysis and Western blot analysis using the indicated antibodies. Images represent three independent experiments. (**K-M, accompanying Fig. 7**) BRAF$^{\Delta\beta3\text{-}\alpha C}$ mutant-expressing cancer cell lines were incubated with naporafenib at the indicated concentrations, alone or combined with 10 or 59 nM XL888. After 96 h, the metabolic activity was measured by XTT assay and normalized to vehicle control. The metabolic activity in the absence of naporafenib is indicated by dotted lines in the colour of the respective XL888 concentration (dark blue: 10 nM XL888, light blue: 59 nM XL888). Graphs show the mean + SD of three independent experiments, Non-linear fitting was calculated using GraphPad Prism 9.

Supplementary Figure S15

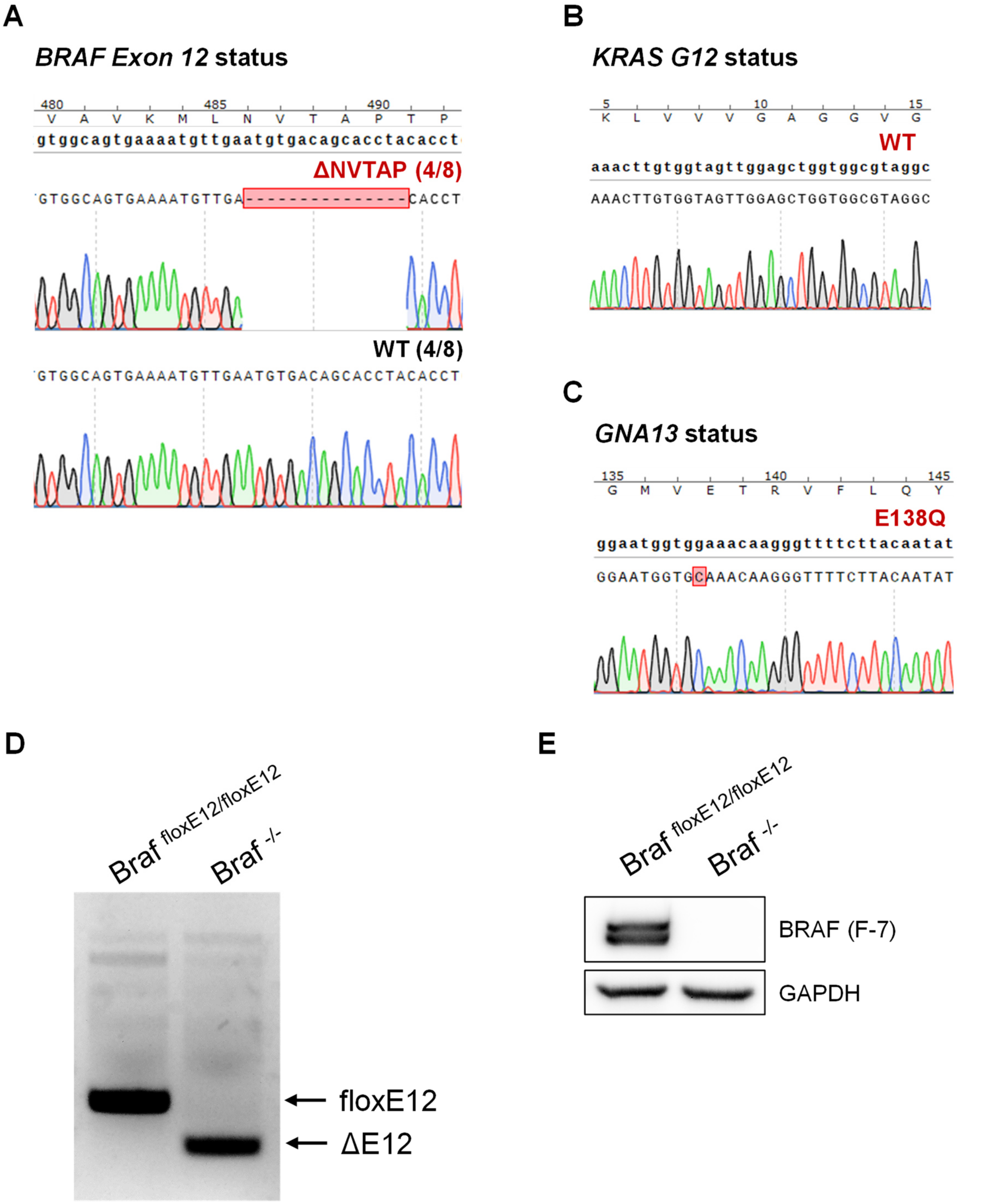

Fig S15. (**A-C, accompanying Fig. 8**) Validation of the *BRAF* in-frame deletion, *GNA13*^{E138Q} mutation and *KRAS* wild-type status of the patient-derived organoid B188 by Sanger sequencing. *KRAS* exon 2, *GNA13* exon 2 and *BRAF* exon 12 were amplified from extracted gDNA and analyzed by sequencing. To separate amplicons derived from wildtype and mutant *BRAF* alleles and to precisely define the in-frame deletion, the *BRAF* amplicons were cloned into pSC-A. The picking of single bacteria colonies and sequencing of their pSC-A plasmids enabled the separate analysis of each allele and confirmed heterozygous ΔNVTAP deletion. Sequence counts from twelve individually analyzed clones are indicated in parentheses. (**D-E**) *Braf* knock-out validation. Cre-mediated recombination was induced by

cultivating pBABE-puro-CreERT2 transduced *Braf*$^{floxE12/floxE12}$ MEFs in the presence of 1 µM 4-hydroxytamoxifen for 24 h (*9*). Successful knock-out of *Braf* was verified by genotyping PCR as described previously (*110*) (**D**). Genomic DNA of CreERT2-negative *Braf*$^{floxE12/floxE12}$ MEFs served as control. BRAF protein levels of *Braf*$^{-/-}$ MEFs were compared to those of CreERT2-negative *Braf*$^{floxE12/floxE12}$ MEFs via Western blot analysis of TCLs using the indicated antibodies (**E**).

Supplementary Figure S16

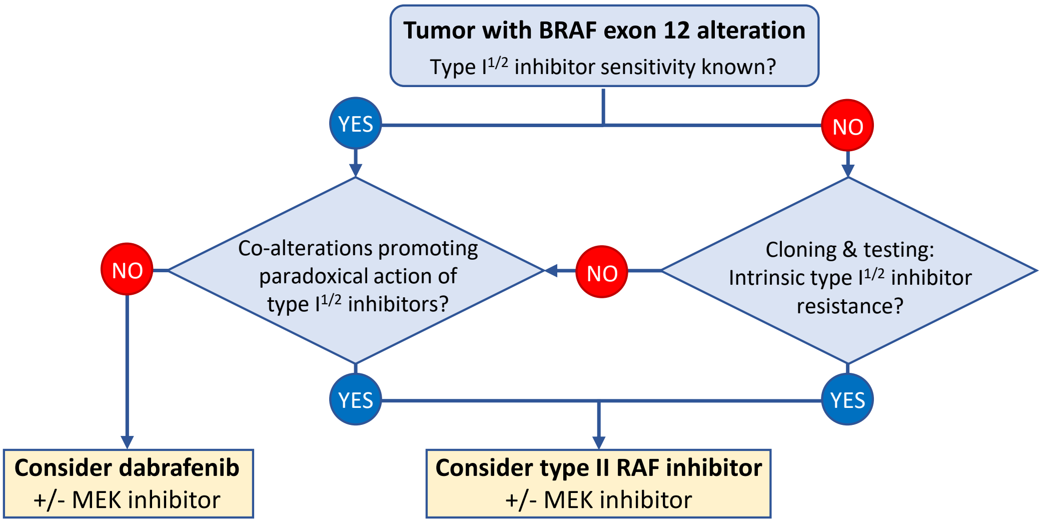

Supplementary Figure S16. Flowchart of our proposed algorithm to stratify BRAF$^{\Delta\beta3\text{-}\alpha C}$ mutant-expressing tumors for dabrafenib or type II RAF inhibitor therapy.

Supplementary Table 1: List of primers used for mutagenesis PCRs.

Primer	5' → 3' sequence
ΔNVTAP_fwd	GGTGATGTGGCAGTGAAAATGTTGACACCTCAGCAGTTACAAGCC
ΔNVTAP_rev	GGCTTGTAACTGCTGAGGTGTCAACATTTTCACTGCCACATCACC
ΔLNVTAP>F_fwd	GGTGATGTGGCAGTGAAAATGTTTACACCTCAGCAGTTACAAGCC
ΔLNVTAP>F_rev	GGCTTGTAACTGCTGAGGTGTAAACATTTTCACTGCCACATCACC
ΔLNVTAP>Y_fwd	GGTGATGTGGCAGTGAAAATGTATACACCTCAGCAGTTACAAGCC
ΔLNVTAP>Y_rev	GGCTTGTAACTGCTGAGGTGTATACATTTTCACTGCCACATCACC
delinsFS_fwd	GGTGATGTGGCAGTGAAAATGTTCTCTACACCTCAGCAGTTACAAGCC
delinsFS_rev	GGCTTGTAACTGCTGAGGTGTAGAGAACATTTTCACTGCCACATCACC
ΔVTAPTP>A_fwd	CAGTGAAAATGTTGAATGCTCAGCAGTTACAAGCCTTC
ΔVTAPTP>A_rev	GAAGGCTTGTAACTGCTGAGCATTCAACATTTTCACTG
ΔLNVT>F_fwd	GATGTGGCAGTGAAAATGTTTGCACCTACACCTCAGCAG
ΔLNVT>F_rev	CTGCTGAGGTGTAGGTGCAAACATTTTCACTGCCACATC
F498A_fwd	GCAGTTACAAGCCGCCAAAAATGAAGTAGG
F498A_rev	CCTACTTCATTTTTGGCGGCTTGTAACTGC
R509H_fwd	GAAGTAGGAGTACTCAGGAAAACACATCATGTGAATATCCTACTCTTCATGGG
R509H_rev	CCCATGAAGAGTAGGATATTCACATGATGTGTTTTCCTGAGTACTCCTACTTC
621APE-AAE_fwd	CATTTTGTGGATGGCAGCAGAAGTCATCAGAATG
621APE-AAE_rev	CATTCTGATGACTTCTGCTGCCATCCACAAAATG

REFERENCES AND NOTES

1. M. L. Turski, S. J. Vidwans, F. Janku, I. Garrido-Laguna, J. Munoz, R. Schwab, V. Subbiah, J. Rodon, R. Kurzrock, Genomically driven tumors and actionability across histologies: BRAF-mutant cancers as a paradigm. *Mol. Cancer Ther.* **15**, 533–547 (2016).

2. F. A. Cook, S. J. Cook, Inhibition of RAF dimers: It takes two to tango. *Biochem. Soc. Trans.* **49**, 237–251 (2021).

3. T. Brummer, C. McInnes, RAF kinase dimerization: Implications for drug discovery and clinical outcomes. *Oncogene* **39**, 4155–4169 (2020).

4. J. A. Martinez Fiesco, D. E. Durrant, D. K. Morrison, P. Zhang, Structural insights into the BRAF monomer-to-dimer transition mediated by RAS binding. *Nat. Commun.* **13**, 486 (2022).

5. Y. Kondo, J. Ognjenović, S. Banerjee, D. Karandur, A. Merk, K. Kulhanek, K. Wong, J. P. Roose, S. Subramaniam, J. Kuriyan, Cryo-EM structure of a dimeric B-Raf:14-3-3 complex reveals asymmetry in the active sites of B-Raf kinases. *Science* **366**, 109–115 (2019).

6. E. Park, S. Rawson, K. Li, B.W. Kim, S. B. Ficarro, G. G.D. Pino, H. Sharif, J. A. Marto, H. Jeon, M. J. Eck, Architecture of autoinhibited and active BRAF–MEK1–14-3-3 complexes. *Nature* **575**, 545–550 (2019).

7. R. Röck, J. E. Mayrhofer, O. Torres-Quesada, F. Enzler, A. Raffeiner, P. Raffeiner, A. Feichtner, R. G. Huber, S. Koide, S. S. Taylor, J. Troppmair, E. Stefan, BRAF inhibitors promote intermediate BRAF(V600E) conformations and binary interactions with activated RAS. *Sci. Adv.* **5**, eaav8463 (2019).

8. T. Rajakulendran, M. Sahmi, M. Lefrancois, F. Sicheri, M. Therrien, A dimerization-dependent mechanism drives RAF catalytic activation. *Nature* **461**, 542–545 (2009).

9. M. Röring, R. Herr, G. J. Fiala, K. Heilmann, S. Braun, A. E. Eisenhardt, S. Halbach, D. Capper, A. von Deimling, W. W. Schamel, D. N. Saunders, T. Brummer, Distinct requirement for an intact dimer interface in wild-type, V600E and kinase-dead B-Raf signalling. *EMBO J.* **31**, 2629–2647 (2012).

10. H. Lavoie, M. Therrien, Regulation of RAF protein kinases in ERK signalling. *Nat. Rev. Mol. Cell Biol.* **16**, 281–298 (2015).

11. A. K. Freeman, D. A. Ritt, D. K. Morrison, Effects of Raf dimerization and its inhibition on normal and disease-associated Raf signaling. *Mol. Cell* **49**, 751–758 (2013).

12. J. Hu, E. C. Stites, H. Yu, E. A. Germino, H. S. Meharena, P. J. S. Stork, A. P. Kornev, S. S. Taylor, A. S. Shaw, Allosteric activation of functionally asymmetric RAF kinase dimers. *Cell* **154**, 1036–1046 (2013).

13. Y. Kondo, J. W. Paul III, S. Subramaniam, J. Kuriyan, New insights into Raf regulation from structural analyses. *Curr. Opin. Struct. Biol.* **71**, 223–231 (2021).

14. H. R. Mott, D. Owen, SHOCing RAF into action. *Nat. Struct. Mol. Biol.* **29**, 958–960 (2022).

15. M. Zhang, R. Maloney, H. Jang, R. Nussinov, The mechanism of Raf activation through dimerization. *Chem. Sci.* **12**, 15609–15619 (2021).

16. B. H. Zhang, K. L. Guan, Activation of B-Raf kinase requires phosphorylation of the conserved residues Thr598 and Ser601. *EMBO J.* **19**, 5429–5439 (2000).

17. N. Thevakumaran, H. Lavoie, D. A. Critton, A. Tebben, A. Marinier, F. Sicheri, M. Therrien, Crystal structure of a BRAF kinase domain monomer explains basis for allosteric regulation. *Nat. Struct. Mol. Biol.* **22**, 37–43 (2015).

18. M. Köhler, M. Röring, B. Schorch, K. Heilmann, N. Stickel, G. J. Fiala, L. C. Schmitt, S. Braun, S. Ehrenfeld, F. M. Uhl, T. Kaltenbacher, F. Weinberg, S. Herzog, R. Zeiser, W. W. Schamel, H. Jumaa, T. Brummer, Activation loop phosphorylation regulates B-Raf in vivo and transformation by B-Raf mutants. *EMBO J.* **35**, 143–161 (2016).

19. B. Agianian, E. Gavathiotis, Current insights of BRAF inhibitors in cancer. *J. Med. Chem.* **61**, 5775–5793 (2018).

20. Z. Karoulia, E. Gavathiotis, P. I. Poulikakos, New perspectives for targeting RAF kinase in human cancer. *Nat. Rev. Cancer* **17**, 676–691 (2017).

21. Z. Yao, N. M. Torres, A. Tao, Y. Gao, L. Luo, Q. Li, E. de Stanchina, O. Abdel-Wahab, D. B. Solit, P. I. Poulikakos, N. Rosen, BRAF Mutants Evade ERK-dependent feedback by different mechanisms that determine their sensitivity to pharmacologic inhibition. *Cancer Cell* **28**, 370–383 (2015).

22. O. S. Rukhlenko, F. Khorsand, A. Krstic, J. Rozanc, L. G. Alexopoulos, N. Rauch, K. E. Erickson, W. S. Hlavacek, R. G. Posner, S. Gómez-Coca, E. Rosta, C. Fitzgibbon, D. Matallanas, J. Rauch, W. Kolch, B. N. Kholodenko, Dissecting RAF inhibitor resistance by structure-based modeling reveals ways to overcome oncogenic RAS signaling. *Cell Syst.* **7**, 161–179.e14 (2018).

23. B. Diedrich, K. T.G. Rigbolt, M. Röring, R. Herr, S. Kaeser-Pebernard, C. Gretzmeier, R. F. Murphy, T. Brummer, J. Dengjel, Discrete cytosolic macromolecular BRAF complexes exhibit distinct activities and composition. *EMBO J.* **36**, 646–663 (2017).

24. J. Yuan, W. H. Ng, P. Y. P. Lam, Y. Wang, H. Xia, J. Yap, S. P. Guan, A. S. G. Lee, M. Wang, M. Baccarini, J. Hu, The dimer-dependent catalytic activity of RAF family kinases is revealed through characterizing their oncogenic mutants. *Oncogene* **37**, 5719–5734 (2018).

25. C. Adamopoulos, T. A. Ahmed, M. R. Tucker, P. M. U. Ung, M. Xiao, Z. Karoulia, A. Amabile, X. Wu, S. A. Aaronson, C. Ang, V. W. Rebecca, B. D. Brown, A. Schlessinger, M. Herlyn, Q. Wang, D. E. Shaw, P. I. Poulikakos, Exploiting allosteric properties of RAF and MEK inhibitors to target therapy-resistant tumors driven by oncogenic BRAF signaling. *Cancer Discov.* **11**, 1716–1735 (2021).

26. M. Dankner, A. A. N. Rose, S. Rajkumar, P. M. Siegel, I. R. Watson, Classifying BRAF alterations in cancer: New rational therapeutic strategies for actionable mutations. *Oncogene* **37**, 3183–3199 (2018).

27. P. T. C. Wan, M. J. Garnett, S Mark Roe, S. Lee, D. Niculescu-Duvaz, V. M. Good, C. M. Jones, C. J. Marshall, C. J. Springer, D. Barford, R. Marais; Cancer Genome Project, Mechanism of activation of the RAF-ERK signaling pathway by oncogenic mutations of B-RAF. *Cell* **116**, 855–867 (2004).

28. S. J. Heidorn, C. Milagre, S. Whittaker, A. Nourry, I. Niculescu-Duvas, N. Dhomen, J. Hussain, J. S. Reis-Filho, C. J. Springer, C. Pritchard, R. Marais, Kinase-dead BRAF and oncogenic RAS cooperate to drive tumor progression through CRAF. *Cell* **140**, 209–221 (2010).

29. P. Nieto, C. Ambrogio, L. Esteban-Burgos, G. Gómez-López, M. T. Blasco, Z. Yao, R. Marais, N. Rosen, R. Chiarle, D. G. Pisano, M. Barbacid, D. Santamaría, A Braf kinase-inactive mutant induces lung adenocarcinoma. *Nature* **548**, 239–243 (2017).

30. M. Dankner, M. Lajoie, D. Moldoveanu, T. T. Nguyen, P. Savage, S. Rajkumar, X. Huang, M. Lvova, A. Protopopov, D. Vuzman, D. Hogg, M. Park, M.C. Guiot, K. Petrecca, C. Mihalcioiu, I. R. Watson, P.

M. Siegel, A. A. N. Rose, Dual MAPK inhibition is an effective therapeutic strategy for a subset of class II BRAF mutant melanomas. *Clin. Cancer Res.* **24**, 6483–6494 (2018).

31. A. J. Aguirre, J. A. Nowak, N. D. Camarda, R. A. Moffitt, A. A. Ghazani, M. Hazar-Rethinam, S. Raghavan, J. Kim, L. K. Brais, D. Ragon, M. W. Welch, E. Reilly, D. McCabe, L. Marini, K. Anderka, K. Helvie, N. Oliver, A. Babic, A. da Silva, B. Nadres, E. E. van Seventer, H. A. Shahzade, J. P. St. Pierre, K. P. Burke, T. Clancy, J. M. Cleary, L. A. Doyle, K. Jajoo, N. J. McCleary, J. A. Meyerhardt, J. E. Murphy, K. Ng, A. K. Patel, K. Perez, M. H. Rosenthal, D. A. Rubinson, M. Ryou, G. I. Shapiro, E. Sicinska, S. G. Silverman, R. J. Nagy, R. B. Lanman, D. Knoerzer, D. J. Welsch, M. B. Yurgelun, C. S. Fuchs, L. A. Garraway, G. Getz, J. L. Hornick, B. E. Johnson, M. H. Kulke, R. J. Mayer, J. W. Miller, P. B. Shyn, D. A. Tuveson, N. Wagle, J. J. Yeh, W. C. Hahn, R. B. Corcoran, S. L. Carter, B. M. Wolpin, Real-time genomic characterization of advanced pancreatic cancer to enable precision medicine. *Cancer Discov.* **8**, 1096–1111 (2018).

32. P. A. Philip, I. Azar, J. Xiu, M. J. Hall, A. E. Hendifar, E. Lou, J. J. Hwang, J. Gong, R. Feldman, M. Ellis, P. Stafford, D. Spetzler, M. M. Khushman, D. Sohal, A. C. Lockhart, B. A. Weinberg, W. S. el-Deiry, J. Marshall, A. F. Shields, W. M. Korn, Molecular characterization of KRAS wild-type tumors in patients with pancreatic adenocarcinoma. *Clin. Cancer Res.* **28**, 2704–2714 (2022).

33. R. Ren, S. G. Krishna, W. Chen, W. L. Frankel, R. Shen, W. Zhao, M. R. Avenarius, J. Garee, S. Caruthers, D. Jones, Activation of the RAS pathway through uncommon BRAF mutations in mucinous pancreatic cysts without KRAS mutation. *Mod. Pathol.* **34**, 438–444 (2021).

34. A. Hendifar, E. M. Blais, B. Wolpin, V. Subbiah, E. Collisson, I. Singh, T. Cannon, K. Shaw, E. F. Petricoin III, S. Klempner, E. Lyons, A. Wang-Gillam, M. J. Pishvaian, E. M. O'Reilly, Retrospective case series analysis of *RAF* family alterations in pancreatic cancer: Real-world outcomes from targeted and standard therapies. *JCO Precis. Oncol.* **5**, PO.20.00494 (2021).

35. S. H. Chen, Y. Zhang, R. D. van Horn, T. Yin, S. Buchanan, V. Yadav, I. Mochalkin, S. S. Wong, Y. G. Yue, L. Huber, I. Conti, J. R. Henry, J. J. Starling, G. D. Plowman, S.B. Peng, Oncogenic braf deletions that function as homodimers and are sensitive to inhibition by RAF dimer inhibitor LY3009120. *Cancer Discov.* **6**, 300–315 (2016).

36. S. A. Foster, D. M. Whalen, A. Özen, M. J. Wongchenko, J.P. Yin, I. Yen, G. Schaefer, J. D. Mayfield, J. Chmielecki, P. J. Stephens, L. A. Albacker, Y. Yan, K. Song, G. Hatzivassiliou, C. Eigenbrot, C. Yu,

A. S. Shaw, G. Manning, N. J. Skelton, S. G. Hymowitz, S. Malek, Activation mechanism of oncogenic deletion mutations in BRAF, EGFR, and HER2. *Cancer Cell* **29**, 477–493 (2016).

37. D. M. Freed, J. H. Park, R. Radhakrishnan, M. A. Lemmon, Deletion mutations keep kinase inhibitors in the loop. *Cancer Cell* **29**, 423–425 (2016).

38. P. Horak, C. Heining, S. Kreutzfeldt, B. Hutter, A. Mock, J. Hüllein, M. Fröhlich, S. Uhrig, A. Jahn, A. Rump, L. Gieldon, L. Möhrmann, D. Hanf, V. Teleanu, C. E. Heilig, D. B. Lipka, M. Allgäuer, L. Ruhnke, A. Laßmann, V. Endris, O. Neumann, R. Penzel, K. Beck, D. Richter, U. Winter, S. Wolf, K. Pfütze, C. Geörg, B. Meißburger, I. Buchhalter, M. Augustin, W. E. Aulitzky, P. Hohenberger, M. Kroiss, P. Schirmacher, R. F. Schlenk, U. Keilholz, F. Klauschen, G. Folprecht, S. Bauer, J. T. Siveke, C. H. Brandts, T. Kindler, M. Boerries, A. L. Illert, N. von Bubnoff, P. J. Jost, K. Spiekermann, M. Bitzer, K. Schulze-Osthoff, C. von Kalle, B. Klink, B. Brors, A. Stenzinger, E. Schröck, D. Hübschmann, W. Weichert, H. Glimm, S. Fröhling, Comprehensive genomic and transcriptomic analysis for guiding therapeutic decisions in patients with rare cancers. *Cancer Discov.* **11**, 2780–2795 (2021).

39. D. Pratt, S. Camelo-Piragua, K. McFadden, D. Leung, R. Mody, A. Chinnaiyan, C. Koschmann, S. Venneti, BRAF activating mutations involving the β3-αC loop in V600E-negative anaplastic pleomorphic xanthoastrocytoma. *Acta Neuropathol. Commun.* **6**, 24 (2018).

40. F. Weinberg, R. Griffin, M. Fröhlich, C. Heining, S. Braun, C. Spohr, M. Iconomou, V. Hollek, M. Röring, P. Horak, S. Kreutzfeldt, G. Warsow, B. Hutter, S. Uhrig, O. Neumann, D. Reuss, D. H. Heiland, C. von Kalle, W. Weichert, A. Stenzinger, B. Brors, H. Glimm, S. Fröhling, T. Brummer, Identification and characterization of a BRAF fusion oncoprotein with retained autoinhibitory domains. *Oncogene* **39**, 814–832 (2020).

41. J. Hu, L. G. Ahuja, H. S. Meharena, N. Kannan, A. P. Kornev, S. S. Taylor, A. S. Shaw, Kinase regulation by hydrophobic spine assembly in cancer. *Mol. Cell. Biol.* **35**, 264–276 (2015).

42. P. I. Poulikakos, Y. Persaud, M. Janakiraman, X. Kong, C. Ng, G. Moriceau, H. Shi, M. Atefi, B. Titz, M. T. Gabay, M. Salton, K. B. Dahlman, M. Tadi, J. A. Wargo, K. T. Flaherty, M. C. Kelley, T. Misteli, P. B. Chapman, J. A. Sosman, T. G. Graeber, A. Ribas, R. S. Lo, N. Rosen, D. B. Solit, RAF inhibitor resistance is mediated by dimerization of aberrantly spliced BRAF(V600E). *Nature* **480**, 387–390 (2011).

43. T. Ikenoue, Y. Hikiba, F. Kanai, Y. Tanaka, J. Imamura, T. Imamura, M. Ohta, H. Ijichi, K. Tateishi, T. Kawakami, J. Aragaki, M. Matsumura, T. Kawabe, M. Omata, Functional analysis of mutations within the kinase activation segment of *B-Raf* in human colorectal tumors. *Cancer Res.* **63**, 8132–8137 (2003).

44. M. Kordes, M. Röring, C. Heining, S. Braun, B. Hutter, D. Richter, C. Geörg, C. Scholl, S. Gröschel, W. Roth, A. Rosenwald, E. Geissinger, C. von Kalle, D. Jäger, B. Brors, W. Weichert, C. Grüllich, H. Glimm, T. Brummer, S. Fröhling, Cooperation of BRAF(F595L) and mutant HRAS in histiocytic sarcoma provides new insights into oncogenic BRAF signaling. *Leukemia* **30**, 937–946 (2016).

45. A. E. Eisenhardt, A. Sprenger, M. Röring, R. Herr, F. Weinberg, M. Köhler, S. Braun, J. Orth, B. Diedrich, U. Lanner, N. Tscherwinski, S. Schuster, N. Dumaz, E. Schmidt, R. Baumeister, A. Schlosser, J. Dengjel, T. Brummer, Phospho-proteomic analyses of B-Raf protein complexes reveal new regulatory principles. *Oncotarget* **7**, 26628–26652 (2016).

46. K. Miyamoto, M. Sawa, Development of highly sensitive biosensors of RAF dimerization in cells. *Sci. Rep.* **9**, 636 (2019).

47. S. da Rocha Dias, F. Friedlos, Y. Light, C. Springer, P. Workman, R. Marais, Activated B-RAF is an Hsp90 client protein that is targeted by the anticancer drug 17-allylamino-17-demethoxygeldanamycin. *Cancer Res.* **65**, 10686–10691 (2005).

48. O. M. Grbovic, A. D. Basso, A. Sawai, Q. Ye, P. Friedlander, D. Solit, N. Rosen, V600E B-Raf requires the Hsp90 chaperone for stability and is degraded in response to Hsp90 inhibitors. *Proc. Natl. Acad. Sci. U.S.A.* **103**, 57–62 (2006).

49. Z. Eroglu, Y. A. Chen, G. T. Gibney, J. S. Weber, R. R. Kudchadkar, N. I. Khushalani, J. Markowitz, A. S. Brohl, L. F. Tetteh, H. Ramadan, G. Arnone, J. Li, X. Zhao, R. Sharma, L. N.F. Darville, B. Fang, I. Smalley, J. L. Messina, J. M. Koomen, V. K. Sondak, K. S.M. Smalley, Combined BRAF and HSP90 inhibition in patients with unresectable BRAF (V600E)-mutant melanoma. *Clin. Cancer Res.* **24**, 5516–5524 (2018).

50. M. A. Hernandez, B. Patel, F. Hey, S. Giblett, H. Davis, C. Pritchard, Regulation of BRAF protein stability by a negative feedback loop involving the MEK-ERK pathway but not the FBXW7 tumour suppressor. *Cell. Signal.* **28**, 561–571 (2016).

51. J. M. Fernandes Neto, E. Nadal, E. Bosdriesz, S. N. Ooft, L. Farre, C. McLean, S. Klarenbeek, A. Jurgens, H. Hagen, L. Wang, E. Felip, A. Martinez-Marti, A. Vidal, E. Voest, L. F. A. Wessels, O. van Tellingen, A. Villanueva, R. Bernards, Multiple low dose therapy as an effective strategy to treat EGFR inhibitor-resistant NSCLC tumours. *Nat. Commun.* **11**, 3157 (2020).

52. R. J. Sullivan, A. Hollebecque, K. T. Flaherty, G. I. Shapiro, J. Rodon Ahnert, M. J. Millward, W. Zhang, L. Gao, A. Sykes, M. D. Willard, D. Yu, A. E. Schade, K. A. Crowe, D. L. Flynn, M. D. Kaufman, J. R. Henry, S.B. Peng, K. A. Benhadji, I. Conti, M. S. Gordon, R. V. Tiu, D. S. Hong, A phase I study of LY3009120, a pan-RAF inhibitor, in patients with advanced or metastatic cancer. *Mol. Cancer Ther.* **19**, 460–467 (2020).

53. I. Yen, F. Shanahan, J. Lee, Y. S. Hong, S. J. Shin, A. R. Moore, J. Sudhamsu, M. T. Chang, I. Bae, D. dela Cruz, T. Hunsaker, C. Klijn, N. P. D. Liau, E. Lin, S. E. Martin, Z. Modrusan, R. Piskol, E. Segal, A. Venkatanarayan, X. Ye, J. Yin, L. Zhang, J.S. Kim, H.S. Lim, K.P. Kim, Y. J. Kim, H. S. Han, S. J. Lee, S. T. Kim, M. Jung, Y.H. Hong, Y. S. Noh, M. Choi, O. Han, M. Nowicka, S. Srinivasan, Y. Yan, T. W. Kim, S. Malek, ARAF mutations confer resistance to the RAF inhibitor belvarafenib in melanoma. *Nature* **594**, 418–423 (2021).

54. K. A. Monaco, S. Delach, J. Yuan, Y. Mishina, P. Fordjour, E. Labrot, D. McKay, R. Guo, S. Higgins, H. Q. Wang, J. Liang, K. Bui, J. Green, P. Aspesi, J. Ambrose, F. Mapa, L. Griner, M. Jaskelioff, J. Fuller, K. Crawford, G. Pardee, S. Widger, P. S. Hammerman, J. A. Engelman, D. D. Stuart, V. G. Cooke, G. Caponigro, LXH254, a potent and selective ARAF-sparing inhibitor of BRAF and CRAF for the treatment of MAPK-driven tumors. *Clin. Cancer Res.* **27**, 2061–2073 (2021).

55. R. Herr, S. Halbach, M. Heizmann, H. Busch, M. Boerries, T. Brummer, BRAF inhibition upregulates a variety of receptor tyrosine kinases and their downstream effector Gab2 in colorectal cancer cell lines. *Oncogene* **37**, 1576–1593 (2018).

56. C. A. Pratilas, B. S. Taylor, Q. Ye, A. Viale, C. Sander, D. B. Solit, N. Rosen, (V600E)BRAF is associated with disabled feedback inhibition of RAF-MEK signaling and elevated transcriptional output of the pathway. *Proc. Natl. Acad. Sci. U.S.A.* **106**, 4519–4524 (2009).

57. J. Phuchareon, F. McCormick, D. W. Eisele, O. Tetsu, EGFR inhibition evokes innate drug resistance in lung cancer cells by preventing Akt activity and thus inactivating Ets-1 function. *Proc. Natl. Acad. Sci. U.S.A.* **112**, E3855–E3863 (2015).

58. N. Gutierrez-Prat, H. L. Zuberer, L. Mangano, Z. Karimaddini, L. Wolf, S. Tyanova, L. C. Wellinger, D. Marbach, V. Griesser, P. Pettazzoni, J. R. Bischoff, D. Rohle, C. Palladino, I. Vivanco, DUSP4 protects BRAF- and NRAS-mutant melanoma from oncogene overdose through modulation of MITF. *Life Sci. Alliance* **5**, e202101235 (2022).

59. D. M. Molina, R. Jafari, M. Ignatushchenko, T. Seki, E. A. Larsson, C. Dan, L. Sreekumar, Y. Cao, P. Nordlund, Monitoring drug target engagement in cells and tissues using the cellular thermal shift assay. *Science* **341**, 84–87 (2013).

60. M. Holderfield, M. M. Deuker, F. McCormick, M. McMahon, Targeting RAF kinases for cancer therapy: BRAF-mutated melanoma and beyond. *Nat. Rev. Cancer* **14**, 455–467 (2014).

61. P. I. Poulikakos, C. Zhang, G. Bollag, K. M. Shokat, N. Rosen, RAF inhibitors transactivate RAF dimers and ERK signalling in cells with wild-type BRAF. *Nature* **464**, 427–430 (2010).

62. D. N. Meijles, J. J. Cull, S. T.E. Cooper, T. Markou, M. A. Hardyman, S. J. Fuller, H. O. Alharbi, Z. H.R. Haines, V. Alcantara-Alonso, P. E. Glennon, M. N. Sheppard, P. H. Sugden, A. Clerk, The anti-cancer drug dabrafenib is not cardiotoxic and inhibits cardiac remodelling and fibrosis in a murine model of hypertension. *Clin. Sci. (Lond.)* **135**, 1631–1647 (2021).

63. T. R. Rheault, J. C. Stellwagen, G. M. Adjabeng, K. R. Hornberger, K. G. Petrov, A. G. Waterson, S. H. Dickerson, R. A. Mook Jr, S. G. Laquerre, A. J. King, O. W. Rossanese, M. R. Arnone, K. N. Smitheman, L. S. Kane-Carson, C. Han, G. S. Moorthy, K. G. Moss, D. E. Uehling, Discovery of dabrafenib: A selective inhibitor of raf kinases with antitumor activity against B-Raf-driven tumors. *ACS Med. Chem. Lett.* **4**, 358–362 (2013).

64. P. Koelblinger, O. Thuerigen, R. Dummer, Development of encorafenib for BRAF-mutated advanced melanoma. *Curr. Opin. Oncol.* **30**, 125–133 (2018).

65. X. M. Cotto-Rios, B. Agianian, N. Gitego, E. Zacharioudakis, O. Giricz, Y. Wu, Y. Zou, A. Verma, P. I. Poulikakos, E. Gavathiotis, Inhibitors of BRAF dimers using an allosteric site. *Nat. Commun.* **11**, 4370 (2020).

66. J. Yap, R. N. V. K. Deepak, Z. Tian, W. H. Ng, K. C. Goh, A. Foo, Z. H. Tee, M. P. Mohanam, Y. R. M. Sim, U. Degirmenci, P. Lam, Z. Chen, H. Fan, J. Hu, The stability of R-spine defines RAF inhibitor

resistance: A comprehensive analysis of oncogenic BRAF mutants with in-frame insertion of αC-β4 loop. *Sci. Adv.* **7**, eabg0390 (2021).

67. D. M. Hyman, I. Puzanov, V. Subbiah, J. E. Faris, I. Chau, J.Y. Blay, J. Wolf, N. S. Raje, E. L. Diamond, A. Hollebecque, R. Gervais, M. E. Elez-Fernandez, A. Italiano, R.D. Hofheinz, M. Hidalgo, E. Chan, M. Schuler, S. F. Lasserre, M. Makrutzki, F. Sirzen, M. L. Veronese, J. Tabernero, J. Baselga, Vemurafenib in multiple nonmelanoma cancers with BRAF V600 mutations. *N. Engl. J. Med.* **373**, 726–736 (2015).

68. M. A. Gouda, V. Subbiah, Precision oncology for BRAF-mutant cancers with BRAF and MEK inhibitors: From melanoma to tissue-agnostic therapy. *ESMO Open* **8**, 100788 (2023).

69. M. H. Tan, N. J. Nowak, R. Loor, H. Ochi, A. A. Sandberg, C. Lopez, J. W. Pickren, R. Berjian, H. O. Douglass, T. M. Chu, Characterization of a new primary human pancreatic tumor line. *Cancer Invest.* **4**, 15–23 (1986).

70. L. G. Ahronian, E. M. Sennott, E. M. van Allen, N. Wagle, E. L. Kwak, J. E. Faris, J. T. Godfrey, K. Nishimura, K. D. Lynch, C. H. Mermel, E. L. Lockerman, A. Kalsy, J. M. Gurski Jr, S. Bahl, K. Anderka, L. M. Green, N. J. Lennon, T. G. Huynh, M. Mino-Kenudson, G. Getz, D. Dias-Santagata, A. J. Iafrate, J. A. Engelman, L. A. Garraway, R. B. Corcoran, Clinical acquired resistance to RAF inhibitor combinations in BRAF-mutant colorectal cancer through MAPK pathway alterations. *Cancer Discov.* **5**, 358–367 (2015).

71. K. S. M. Smalley, M. Xiao, J. Villanueva, T. K. Nguyen, K. T. Flaherty, R. Letrero, P. Van Belle, D. E. Elder, Y. Wang, K. L. Nathanson, M. Herlyn, CRAF inhibition induces apoptosis in melanoma cells with non-V600E BRAF mutations. *Oncogene* **28**, 85–94 (2009).

72. S. M. Wilhelm, C. Carter, L.Y. Tang, D. Wilkie, A. McNabola, H. Rong, C. Chen, X. Zhang, P. Vincent, M. McHugh, Y. Cao, J. Shujath, S. Gawlak, D. Eveleigh, B. Rowley, L. Liu, L. Adnane, M. Lynch, D. Auclair, I. Taylor, R. Gedrich, A. Voznesensky, B. Riedl, L. E. Post, G. Bollag, P. A. Trail, BAY 43-9006 exhibits broad spectrum oral antitumor activity and targets the RAF/MEK/ERK pathway and receptor tyrosine kinases involved in tumor progression and angiogenesis. *Cancer Res.* **64**, 7099–7109 (2004).

73. D. Strumberg, H. Richly, R. A. Hilger, N. Schleucher, S. Korfee, M. Tewes, M. Faghih, E. Brendel, D. Voliotis, C. G. Haase, B. Schwartz, A. Awada, R. Voigtmann, M. E. Scheulen, S. Seeber, Phase I

clinical and pharmacokinetic study of the Novel Raf kinase and vascular endothelial growth factor receptor inhibitor BAY 43-9006 in patients with advanced refractory solid tumors. *J. Clin. Oncol.* **23**, 965–972 (2005).

74. A. Awada, A. Hendlisz, T. Gil, S. Bartholomeus, M. Mano, D. de Valeriola, D. Strumberg, E. Brendel, C. G. Haase, B. Schwartz, M. Piccart, Phase I safety and pharmacokinetics of BAY 43-9006 administered for 21 days on/7 days off in patients with advanced, refractory solid tumours. *Br. J. Cancer* **92**, 1855–1861 (2005).

75. R. J. Sullivan, J. R. Infante, F. Janku, D. J. L. Wong, J. A. Sosman, V. Keedy, M. R. Patel, G. I. Shapiro, J. W. Mier, A. W. Tolcher, A. Wang-Gillam, M. Sznol, K. Flaherty, E. Buchbinder, R. D. Carvajal, A. M. Varghese, M. E. Lacouture, A. Ribas, S. P. Patel, G. A. DeCrescenzo, C. M. Emery, A. L. Groover, S. Saha, M. Varterasian, D. J. Welsch, D. M. Hyman, B. T. Li, First-in-class ERK1/2 inhibitor ulixertinib (BVD-523) in patients with MAPK mutant advanced solid tumors: Results of a phase I dose-escalation and expansion study. *Cancer Discov.* **8**, 184–195 (2018).

76. R. Sigaud, L. Rösch, C. Gatzweiler, J. Benzel, L. von Soosten, H. Peterziel, F. Selt, S. Najafi, S. Ayhan, X. F. Gerloff, N. Hofmann, I. Büdenbender, L. Schmitt, K. I. Foerster, J. Burhenne, W. E. Haefeli, A. Korshunov, F. Sahm, C. M. van Tilburg, D. T. W. Jones, S. M. Pfister, D. Knoerzer, B. L. Kreider, M. Sauter, K. W. Pajtler, M. Zuckermann, I. Oehme, O. Witt, T. Milde, The first-in-class ERK inhibitor ulixertinib shows promising activity in mitogen-activated protein kinase (MAPK)-driven pediatric low-grade glioma models. *Neuro Oncol.* **25**, 566–579 (2023).

77. M. Ghasemi, T. Turnbull, S. Sebastian, I. Kempson, The MTT assay: Utility, limitations, pitfalls, and interpretation in bulk and single-cell analysis. *Int. J. Mol. Sci.* **22**, 12827 (2021).

78. A. Prahallad, C. Sun, S. Huang, F. di Nicolantonio, R. Salazar, D. Zecchin, R. L. Beijersbergen, A. Bardelli, R. Bernards, Unresponsiveness of colon cancer to BRAF(V600E) inhibition through feedback activation of EGFR. *Nature* **483**, 100–103 (2012).

79. R. B. Corcoran, H. Ebi, A. B. Turke, E. M. Coffee, M. Nishino, A. P. Cogdill, R. D. Brown, P. Della Pelle, D. Dias-Santagata, K. E. Hung, K. T. Flaherty, A. Piris, J. A. Wargo, J. Settleman, M. Mino-Kenudson, J. A. Engelman, EGFR-mediated re-activation of MAPK signaling contributes to insensitivity of BRAF mutant colorectal cancers to RAF inhibition with vemurafenib. *Cancer Discov.* **2**, 227–235 (2012).

80. K. Zmajkovicova, V. Jesenberger, F. Catalanotti, C. Baumgartner, G. Reyes, M. Baccarini, MEK1 is required for PTEN membrane recruitment, AKT regulation, and the maintenance of peripheral tolerance. *Mol. Cell* **50**, 43–55 (2013).

81. B. A. Hemmings, D. F. Restuccia, PI3K-PKB/Akt pathway. *Cold Spring Harb. Perspect. Biol.* **4**, a011189 (2012).

82. D. Brauswetter, B. Gurbi, A. Varga, E. Várkondi, R. Schwab, G. Bánhegyi, O. Fábián, G. Kéri, I. Vályi-Nagy, I. Peták, Molecular subtype specific efficacy of MEK inhibitors in pancreatic cancers. *PLOS ONE* **12**, e0185687 (2017).

83. R. Hoefflin, A.L. Geißler, R. Fritsch, R. Claus, J. Wehrle, P. Metzger, M. Reiser, L. Mehmed, L. Fauth, D. H. Heiland, T. Erbes, F. Stock, A. Csanadi, C. Miething, B. Weddeling, F. Meiss, D. von Bubnoff, C. Dierks, I. Ge, V. Brass, S. Heeg, H. Schäfer, M. Boeker, J. Rawluk, E. M. Botzenhart, G. Kayser, S. Hettmer, H. Busch, C. Peters, M. Werner, J. Duyster, T. Brummer, M. Boerries, S. Lassmann, N. von Bubnoff, Personalized clinical decision making through implementation of a molecular tumor board: A german single-center experience. *JCO Precis. Oncol.* **2**, 1–16 (2018).

84. R. Kim, E. Tan, E. Wang, A. Mahipal, D.T. Chen, B. Cao, F. Masawi, C. Machado, J. Yu, D. W. Kim, A phase I trial of trametinib in combination with sorafenib in patients with advanced hepatocellular cancer. *Oncologist* **25**, e1893–e1899 (2020).

85. F. de Braud, C. Dooms, R. S. Heist, C. Lebbe, M. Wermke, A. Gazzah, D. Schadendorf, P. Rutkowski, J. Wolf, P. A. Ascierto, I. Gil-Bazo, S. Kato, M. Wolodarski, M. McKean, E. Muñoz Couselo, M. Sebastian, A. Santoro, V. Cooke, L. Manganelli, K. Wan, A. Gaur, J. Kim, G. Caponigro, X. M. Couillebault, H. Evans, C. D. Campbell, S. Basu, M. Moschetta, A. Daud, Initial evidence for the efficacy of naporafenib in combination with trametinib in NRAS-mutant melanoma: Results from the expansion arm of a phase Ib, open-label study. *J. Clin. Oncol.* **41**, 2651–2660 (2023).

86. M. Phadke, G. T. Gibney, C. J. Rich, I. V. Fedorenko, Y. A. Chen, R. R. Kudchadkar, V. K. Sondak, J. Weber, J. L. Messina, K. S. M. Smalley, XL888 limits vemurafenib-induced proliferative skin events by suppressing paradoxical MAPK activation. *J. Invest. Dermatol.* **135**, 2542–2544 (2015).

87. S. A. K. Rasheed, L. V. Subramanyan, W. K. Lim, U. K. Udayappan, M. Wang, P. J. Casey, The emerging roles of Gα12/13 proteins on the hallmarks of cancer in solid tumors. *Oncogene* **41**, 147–158 (2022).

88. J. X. Zhang, M. Yun, Y. Xu, J.W. Chen, H.W. Weng, Z.S. Zheng, C. Chen, D. Xie, S. Ye, GNA13 as a prognostic factor and mediator of gastric cancer progression. *Oncotarget* **7**, 4414–4427 (2016).

89. K. O. Wrzeszczynski, S. Rahman, M. O. Frank, K. Arora, M. Shah, H. Geiger, V. Felice, D. Manaa, E. Dikoglu, D. Khaira, A. R. Chimpiri, V. V. Michelini, V. Jobanputra, R. B. Darnell, S. Powers, M. Choi, Identification of targetable BRAF ΔN486_P490 variant by whole-genome sequencing leading to dabrafenib-induced remission of a BRAF-mutant pancreatic adenocarcinoma. *Cold Spring Harb. Mol. Case Stud.* **5**, a004424 (2019).

90. A. D. Singhi, B. George, J. R. Greenbowe, J. Chung, J. Suh, A. Maitra, S. J. Klempner, A. Hendifar, J. M. Milind, T. Golan, R. E. Brand, A. H. Zureikat, S. Roy, A. B. Schrock, V. A. Miller, J. S. Ross, S. M. Ali, N. Bahary, Real-time targeted genome profile analysis of pancreatic ductal adenocarcinomas identifies genetic alterations that might be targeted with existing drugs or used as biomarkers. *Gastroenterology* **156**, 2242–2253.e4 (2019).

91. F. Bray, J. Ferlay, I. Soerjomataram, R.L. Siegel, L.A. Torre, A. Jemal, Global cancer statistics 2018: GLOBOCAN estimates of incidence and mortality worldwide for 36 cancers in 185 countries. *CA Cancer J. Clin.* **68**, 394–424 (2018).

92. R. Thomas, C. A. Wiley, E. L. Droste, J. Robertson, B. A. Inman, M. Breen, Whole exome sequencing analysis of canine urothelial carcinomas without BRAF V595E mutation: Short in-frame deletions in BRAF and MAP2K1 suggest alternative mechanisms for MAPK pathway disruption. *PLOS Genet.* **19**, e1010575 (2023).

93. A. S. Shaw, A. P. Kornev, J. Hu, L. G. Ahuja, S. S. Taylor, Kinases and pseudokinases: Lessons from RAF. *Mol. Cell. Biol.* **34**, 1538–1546 (2014).

94. E. L. Diamond, B. H. Durham, G. A. Ulaner, E. Drill, J. Buthorn, M. Ki, L. Bitner, H. Cho, R. J. Young, J. H. Francis, R. Rampal, M. Lacouture, L. A. Brody, N. Ozkaya, A. Dogan, N. Rosen, A. Iasonos, O. Abdel-Wahab, D. M. Hyman, Efficacy of MEK inhibition in patients with histiocytic neoplasms. *Nature* **567**, 521–524 (2019).

95. Z. Eroglu, A. Ribas, Combination therapy with BRAF and MEK inhibitors for melanoma: Latest evidence and place in therapy. *Ther. Adv. Med. Oncol.* **8**, 48–56 (2016).

96. I. Ozkan-Dagliyan, J. N. Diehl, S. D. George, A. Schaefer, B. Papke, K. Klotz-Noack, A. M. Waters, C. M. Goodwin, P. Gautam, M. Pierobon, S. Peng, T. S.K. Gilbert, K. H. Lin, O. Dagliyan, K. Wennerberg, E. F. Petricoin III, N. L. Tran, S. V. Bhagwat, R. V. Tiu, S.B. Peng, L. E. Herring, L. M. Graves, C. Sers, K. C. Wood, A. D. Cox, C. J. der, Low-dose vertical inhibition of the RAF-MEK-ERK cascade causes apoptotic death of KRAS mutant cancers. *Cell Rep.* **31**, 107764 (2020).

97. J. E. Shin, H. J. An, H. S. Park, H. Kim, B. Y. Shim, Efficacy of dabrafenib/trametinib in pancreatic ductal adenocarcinoma with BRAF NVTAP deletion: A case report. *Front. Oncol.* **12**, 976450 (2022).

98. R. Renier, P. De Haes, F. Bosisio, I. V. Bempt, A. J. F. Woei, Vulvar Langerhans cell histiocytosis: Clinicopathologic characteristics, mutational profile, and treatment of 4 patients in a single-center cohort. *JAAD Case Rep.* **36**, 78–81 (2023).

99. S. Zhang, Z. Yang, Y. Cheng, X. Guo, C. Liu, S. Wang, L. Zhang, BRAF L485-P490 deletion mutant metastatic melanoma sensitive to BRAF and MEK inhibition: A case report and literature review. *Front. Pharmacol.* **13**, 1019217 (2022).

100. J. Oberoi, X. A. Guiu, E. A. Outwin, P. Schellenberger, T. I. Roumeliotis, J. S. Choudhary, L. H. Pearl, HSP90-CDC37-PP5 forms a structural platform for kinase dephosphorylation. *Nat. Commun.* **13**, 7343 (2022).

101. S. García-Alonso, P. Mesa, L. de la Puente Ovejero, G. Aizpurua, C. G. Lechuga, E. Zarzuela, C. M. Santiveri, M. Sanclemente, J. Muñoz, M. Musteanu, R. Campos-Olivas, J. Martínez-Torrecuadrada, M. Barbacid, G. Montoya, Structure of the RAF1-HSP90-CDC37 complex reveals the basis of RAF1 regulation. *Mol. Cell* **82**, 3438–3452.e8 (2022).

102. D. Keramisanou, M. V. Vasantha Kumar, N. Boose, R. R. Abzalimov, I. Gelis, Assembly mechanism of early Hsp90-Cdc37-kinase complexes. *Sci. Adv.* **8**, eabm9294 (2022).

103. D. M. Bjorklund, R. M. L. Morgan, J. Oberoi, K. L. I. M. Day, P. A. Galliou, C. Prodromou, Recognition of BRAF by CDC37 and reevaluation of the activation mechanism for the class 2 BRAF-L597R mutant. *Biomolecules* **12**, 905 (2022).

104. P. Horak, B. Klink, C. Heining, S. Gröschel, B. Hutter, M. Fröhlich, S. Uhrig, D. Hübschmann, M. Schlesner, R. Eils, D. Richter, K. Pfütze, C. Geörg, B. Meißburger, S. Wolf, A. Schulz, R. Penzel, E. Herpel, M. Kirchner, A. Lier, V. Endris, S. Singer, P. Schirmacher, W. Weichert, A. Stenzinger, R. F.

Schlenk, E. Schröck, B. Brors, C. von Kalle, H. Glimm, S. Fröhling, Precision oncology based on omics data: The NCT Heidelberg experience. *Int. J. Cancer* **141**, 877–886 (2017).

105. G. M. O'Kane, B. T. Grünwald, G.-H. Jang, M. Masoomian, S. Picardo, R. C. Grant, R. E. Denroche, A. Zhang, Y. Wang, B. Lam, P. M. Krzyzanowski, I. M. Lungu, J. M. S. Bartlett, M. Peralta, F. Vyas, R. Khokha, J. Biagi, D. Chadwick, S. Ramotar, S. Hutchinson, A. Dodd, J. M. Wilson, F. Notta, G. Zogopoulos, S. Gallinger, J. J. Knox, S. E. Fischer, GATA6 expression distinguishes classical and basal-like subtypes in advanced pancreatic cancer. *Clin. Cancer Res.* **26**, 4901–4910 (2020).

106. K. L. Aung, S. E. Fischer, R. E. Denroche, G.H. Jang, A. Dodd, S. Creighton, B. Southwood, S.B. Liang, D. Chadwick, A. Zhang, G. M. O'Kane, H. Albaba, S. Moura, R. C. Grant, J. K. Miller, F. Mbabaali, D. Pasternack, I. M. Lungu, J. M.S. Bartlett, S. Ghai, M. Lemire, S. Holter, A. A. Connor, R. A. Moffitt, J. J. Yeh, L. Timms, P. M. Krzyzanowski, N. Dhani, D. Hedley, F. Notta, J. M. Wilson, M. J. Moore, S. Gallinger, J. J. Knox, Genomics-driven precision medicine for advanced pancreatic cancer: Early results from the COMPASS trial. *Clin. Cancer Res.* **24**, 1344–1354 (2018).

107. N. A. Pham, N. Radulovich, E. Ibrahimov, S. N. Martins-Filho, Q. Li, M. Pintilie, J. Weiss, V. Raghavan, M. Cabanero, R. E. Denroche, J. M. Wilson, C. Metran-Nascente, A. Borgida, S. Hutchinson, A. Dodd, M. Begora, D. Chadwick, S. Serra, J. J. Knox, S. Gallinger, D. W. Hedley, L. Muthuswamy, M. S. Tsao, Patient-derived tumor xenograft and organoid models established from resected pancreatic, duodenal and biliary cancers. *Sci. Rep.* **11**, 10619 (2021).

108. L. A. Baker, H. Tiriac, D. A. Tuveson, Generation and culture of human pancreatic ductal adenocarcinoma organoids from resected tumor specimens. *Methods Mol. Biol.* **1882**, 97–115 (2019).

109. S. F. Boj, C.I. Hwang, L. A. Baker, D. D. Engle, D. A. Tuveson, H. Clevers, Model organoids provide new research opportunities for ductal pancreatic cancer. *Mol. Cell. Oncol.* **3**, e1014757 (2016).

110. G. Galabova-Kovacs, D. Matzen, D. Piazzolla, K. Meissl, T. Plyushch, A. P. Chen, A. Silva, M. Baccarini, Essential role of B-Raf in ERK activation during extraembryonic development. *Proc. Natl. Acad. Sci. U.S.A.* **103**, 1325–1330 (2006).

111. J. Albers, C. Danzer, M. Rechsteiner, H. Lehmann, L. P. Brandt, T. Hejhal, A. Catalano, P. Busenhart, A. F. Gonçalves, S. Brandt, P. K. Bode, B. Bode-Lesniewska, P. J. Wild, I. J. Frew, A versatile modular vector system for rapid combinatorial mammalian genetics. *J. Clin. Invest.* **125**, 1603–1619 (2015).

112. J. Cox, M. Mann, MaxQuant enables high peptide identification rates, individualized p.p.b.-range mass accuracies and proteome-wide protein quantification. *Nat. Biotechnol.* **26**, 1367–1372 (2008).

113. N. P. D. Liau, A. Venkatanarayan, J. G. Quinn, W. Phung, S. Malek, S. G. Hymowitz, J. Sudhamsu, Dimerization induced by C-terminal 14-3-3 binding is sufficient for BRAF kinase activation. *Biochemistry* **59**, 3982–3992 (2020).

114. B. Zhang, Y. Chen, P. Dai, H. Yu, J. Ma, C. Chen, Y. Zhang, Y. Guan, R. Chen, T. Liu, J. Wang, L. Yang, X. Yi, X. Xia, H. Ma, Oncogenic mutations within the beta3-alphaC loop of EGFR/ERBB2/BRAF/MAP2K1 predict response to therapies. *Mol. Genet. Genomic Med.* **8**, e1395 (2020).

115. B. S. White, I. Lanc, J. O'Neal, H. Gupta, R. S. Fulton, H. Schmidt, C. Fronick, E. A. Belter Jr., M. Fiala, J. King, G. J. Ahmann, M. DeRome, E. R. Mardis, R. Vij, J. F. DiPersio, J. Levy, D. Auclair, M. H. Tomasson, A multiple myeloma-specific capture sequencing platform discovers novel translocations and frequent, risk-associated point mutations in IGLL5. *Blood Cancer J.* **8**, 35 (2018).

116. M. Mirdita, K. Schütze, Y. Moriwaki, L. Heo, S. Ovchinnikov, M. Steinegger, ColabFold: Making protein folding accessible to all. *Nat. Methods* **19**, 679–682 (2022).
